# Functional Model of Compound II of Cytochrome P450: Spectroscopic Characterization and Reactivity Studies of a Fe^IV^–OH Complex

**DOI:** 10.1021/jacsau.3c00844

**Published:** 2024-03-11

**Authors:** Kritika Keshari, Aakash Santra, Lucía Velasco, Maxime Sauvan, Simarjeet Kaur, Ashok D. Ugale, Sandip Munshi, J. F. Marco, Dooshaye Moonshiram, Sayantan Paria

**Affiliations:** † Department of Chemistry, Indian Institute of Technology Delhi, Hauz Khas, New Delhi 110016, India; ‡ Instituto de Ciencia de Materiales de Madrid, Consejo Superior de Investigaciones Científicas, Sor Juana Inés de la Cruz, 3, Madrid 28049, Spain; § School of Chemical Science, Indian Association for the Cultivation of Science, Raja S C Mulliick Road, Kolkata 700032, India; ∥ Instituto de Quimica Fisica Blas Cabrera, Consejo Superior de Investigaciones Científicas, C. de Serrano, 119, Serrano, Madrid 28006, Spain

**Keywords:** iron(IV) hydroxide, iron(IV) methoxide, compound II mimic, hydroxide rebound, PCET, oxygen atom transfer

## Abstract

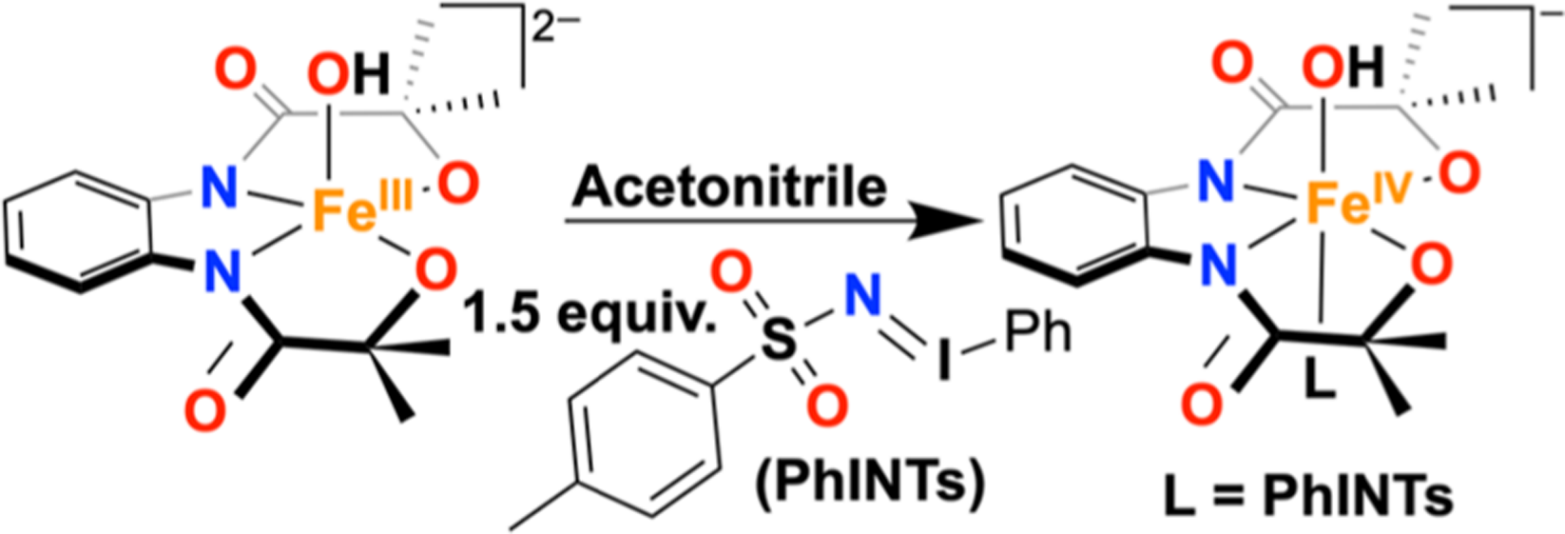

Herein, we show that the reaction of a mononuclear Fe^III^(OH) complex (**1**) with *N*-tosyliminobenzyliodinane (PhINTs) resulted in the formation of a Fe^IV^(OH) species (**3**). The obtained complex **3** was characterized by an array of spectroscopic techniques and represented a rare example of a synthetic Fe^IV^(OH) complex. The reaction of **1** with the one-electron oxidizing agent was reported to form a ligand-oxidized Fe^III^(OH) complex (**2**). **3** revealed a one-electron reduction potential of −0.22 V vs Fc^+^/Fc at −15 °C, which was 150 mV anodically shifted than **2** (*E*_red_ = −0.37 V vs Fc^+^/Fc at −15 °C), inferring **3** to be more oxidizing than **2**. **3** reacted spontaneously with (4-OMe-C_6_H_4_)_3_C^•^ to form (4-OMe-C_6_H_4_)_3_C(OH) through rebound of the OH group and displayed significantly faster reactivity than **2**. Further, activation of the hydrocarbon C–H and the phenolic O–H bond by **2** and **3** was compared and showed that **3** is a stronger oxidant than **2**. A detailed kinetic study established the occurrence of a concerted proton–electron transfer/hydrogen atom transfer reaction of **3**. Studying one-electron reduction of **2** and **3** using decamethylferrocene (Fc*) revealed a higher *k*_et_ of **3** than **2**. The study established that the primary coordination sphere around Fe and the redox state of the metal center is very crucial in controlling the reactivity of high-valent Fe–OH complexes. Further, a Fe^III^(OMe) complex (**4**) was synthesized and thoroughly characterized, including X-ray structure determination. The reaction of **4** with PhINTs resulted in the formation of a Fe^IV^(OMe) species (**5**), revealing the presence of two Fe^IV^ species with isomer shifts of −0.11 mm/s and = 0.17 mm/s in the Mössbauer spectrum and showed Fe^IV^/Fe^III^ potential at −0.36 V vs Fc^+^/Fc couple in acetonitrile at −15 °C. The reactivity studies of **5** were investigated and compared with the Fe^IV^(OH) complex (**3**).

## Introduction

**Scheme 1:**
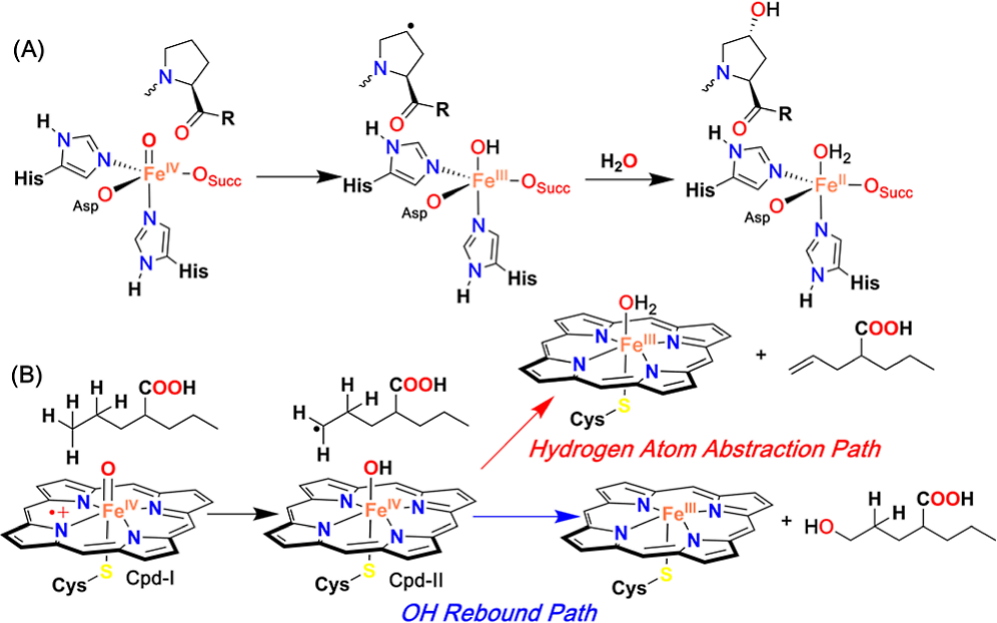
(A) Alkane Hydroxylation Reactions Catalyzed by Prolyl-4-hydroxylases (α-KG-Dependent Oxygenases); (B) Hydroxylation versus Hydrogen Atom Abstraction Pathway in the Metabolism of Valproic Acid Catalyzed by CYP

Regioselective hydroxylation of alkanes in biological systems is catalyzed by several heme and nonheme enzymes.^1−3^ A large family of α-ketoglutarate (α-KG)-dependent oxygenases, containing a 2-His-1-carboxylate facial coordination motif around the Fe^II^ active site, generates Fe^IV^=O species using O_2_ as the oxidant and α-KG as the sacrificial substrate,^4,5^ which then abstracts the hydrogen atom from the substrate to form Fe^III^(OH) and a carbon-centered substrate radical. The subsequent rebound of the OH group forms the hydroxylated product and Fe(II). An example of this family of enzymes is prolyl-4-hydroxylases, whose function is described in Scheme 1A. However, in a large family of cytochrome P450 (CYP) enzymes, Fe^IV^=O porphyrin π-cation radical species, commonly known as Compound I (Cpd-I), cleaves the hydrocarbon C–H bond at the rate-determining step, thus ensuing fast rebound of the OH group from the formed Fe^IV^(OH) (Compound-II or Cpd-II) to the substrate radical to generate the C–OH bond and a Fe(III)–porphyrin complex (Scheme 1B).^2^ Since the rebound step is very fast, direct observation of the C–OH bond formation step is challenging. The C–H activation studies of several Fe^*n*+^(O) (*n* = IV or V) model compounds revealed the formation of a hydroxylated product and Fe(II) or Fe(III) complexes without spectroscopic detection of Fe^*n*+^(OH) (*n* = III or IV) species.^6−9^ Nonetheless, Cpd-II has been characterized in the enzymatic system in a couple of cases.^10−12^

In addition to the archetypal OH rebound reaction of Cpd-II in CYP monooxygenases, the involvement of the intermediate in the direct hydrogen atom abstraction (HAA) or proton-coupled oxidation reaction to form a H_2_O-coordinated [Fe^III^(porphyrin)(cystine)] complex has been described.

Examples of such reactions are the C–C bond cleavage of fatty acids by OleT (bacterial CYP),^13−15^ the third step of oxidation of androgens to estrogens by a steroid aromatase CYP 19A1,^16^ desaturation of valproic acid to 2-propyl-4-pentenoic acid by liver CYP,^17^ etc. Thus, considerable importance was given to elucidating the reaction mechanism of Cpd-II for HAA reactions.^18,19^ In a recent investigation by Green and Mittra, the bond dissociation free energy (BDFE) of the O–H bond of [Fe^III^(H_2_O)(porphyrin)(cystine)] generated from Cpd-II was experimentally determined to be 90 kcal/mol.^20^

Thus, exploring the reactivity of biomimetic Fe(OH) species has attracted considerable attention. The focus has been given to independently synthesizing Fe^*n*+^(OH) complexes and studying their reactivities to gain an insight into the OH rebound and HAA reaction mechanisms. Goldberg et al. reported the spectroscopic characterization and OH rebound studies of a Fe^IV^(OH) complex of a corrole ligand.^21,22^ Further, it was shown that the species could participate in the hydrogen atom transfer (HAT) reactions.^23^ They also investigated the rebound mechanism studies of Fe^III^(X) (X = –OH, –OMe, –N_3_, and –NCS) species with triaryl methyl radical species.^24−27^ Fout et al. reported the characterization and OH rebound studies of Fe^III^(OH) complexes.^28^ Further, the reactivity studies of a couple of synthetic Fe^III^(OH) toward the activation of alkane C–H and phenolic O–H bonds have been studied. Borovik et al. described a thorough spectroscopic characterization of a protonated Fe^IV^(O) species.^29^ In this study, they suggested that the protonation most likely occurred at the ligand backbone, which made intramolecular hydrogen bonds to stabilize the Fe=O moiety. Further, inspired by the mechanistic cycle of CYP, Nam et al. reported the spectroscopic characterization and nitrogen group rebound studies of a Fe(IV)–amido complex (Fe^IV^–NHR), which was synthesized from a Fe(V)–imido (Fe^V^=NR) species via a HAT reaction.^30^

Recent studies by Green et al. showed that the basicity of the coordinated OH group of Cpd-II plays a key role in controlling the reactivity, and the p*K*_a_ of the OH group was determined by the axial ligand present trans to the Fe–OH bond in Cpd-II.^11^ The coordination of the thiolate ligand at the axial position was suggested to decrease the reduction potential and increase the p*K*_a_ of Fe^IV^(OH). Very high p*K*_a_ (>10) of a couple of Cpd-II intermediates has been determined experimentally.^11,31,32^ However, the examples describing the structure and function relationship are lacking for the artificial analogues Cpd-II. Further, limited information is available about the redox properties and reactivities of Fe^IV^(OH) complexes toward OH rebound, proton-coupled electron transfer (PCET), and oxygen atom transfer (OAT) reactions. Compared to the large number of examples reported for the biomimetic Fe^*n*+^=O species, only one example is known describing detailed characterization and OH rebound reactivity studies of a synthetic Fe^IV^(OH) complex.^21^ Thus, we sought to explore the coordination chemistry of synthetic Fe^IV^(OH) species. In this study, we report a detailed characterization and reactivity study of a synthetic Fe^IV^OH complex (**3**), which was prepared by reacting Fe^III^(OH) complex (**1**) of a tetraanionic N_2_O_2_ donor ligand HMPAB^4–^ (H_4_HMPAB = 1,2-bis(2-hydroxy-2-methylpropanamido)benzene) with an excess of *N*-tosyliminobenzyliodinane (PhINTs) in acetonitrile at low temperatures. The reactivity of **3** was compared to the ligand radical-coordinated Fe^III^(OH) complex (**2**). Additionally, we describe the preparation of a Fe^III^(OMe) complex of HMPAB ([Fe^III^(HMPAB)(OMe)]^2−^ (**4**)). The reaction of **4** was investigated with PhINTs, which resulted in the generation of a Fe^IV^(OMe) species (**5**) which was characterized and whose reactivity studies were further investigated.

## Results and Discussion

### Synthesis and Characterization of Fe^III^ Complexes

**Figure 1:**
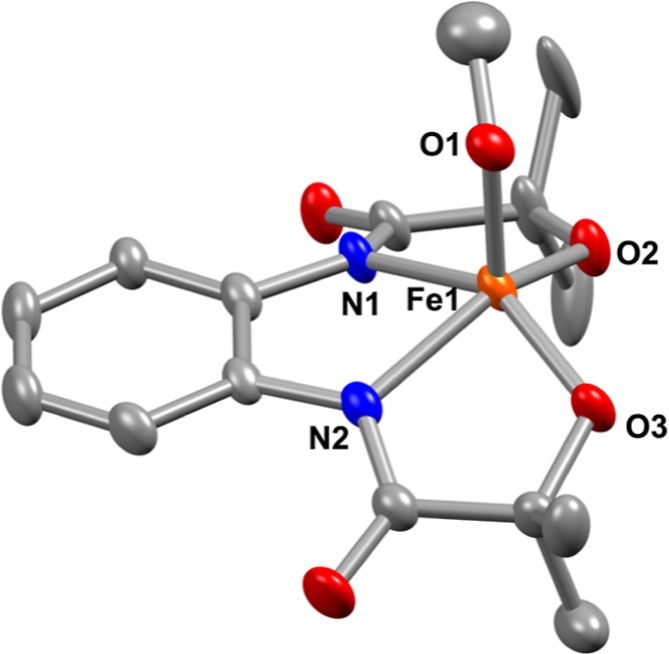
X-ray structure of **4** with 50% ellipsoid probability. The hydrogen atoms of the ligand and countercations are removed for the sake of clarity. CCDC number: 2321489.

The synthesis and characterization of the Fe^III^(OH) complex (**1**) were reported by us recently.^33^ We further prepared the Fe^III^(OMe) complex (**4**), by reacting equimolar amounts of H_4_HMPAB and FeCl_3_ in methanol in the presence of Me_4_NOH as the base under anaerobic conditions (details are described in the Methods). The compound was crystallized by diffusing diethyl ether into an acetonitrile solution of **4**. The X-ray structure of **4** is described in Figure 1. A distorted square-pyramidal geometry around Fe is noted in **4** (τ_5_ = 0.097).^34^ The Fe–N_amide_ (2.059(3) and 2.056(3) Å) and Fe–O_alkoxide_ (1.912(3) and 1.914(3) Å) bond distances are comparable to that of the Fe^III^(OH) analogue (**1**). The Fe–OCH_3_ distance of 1.889(3) Å was noted in **4**, which is slightly shorter than the Fe–OH distance observed in **1** (*d*_Fe–OH_ = 1.9093 (17) Å). The crystallographic parameters and important bond distances of **4** are described in Tables S1 and S2.

The UV–vis spectrum of **4** was measured in acetonitrile, which exhibited broad peaks at 360 and 485 nm (Figure S3). The X-band electron paramagnetic resonance (EPR) spectrum of **4** in frozen tetrahydrofuran/methanol (5:2) at 77 K revealed g values at 5.9 and 2.0 (Figure S4), suggesting the presence of high-spin Fe^III^ (*S* = 5/2) in **4**. Further, we determined the solution magnetic moment of **4** by Evans’ method, which also revealed the existence of *S* = 5/2 Fe in **4** (μ_eff_ = 4.9 μ_B_ in CD_3_OD at 25 °C, Figure S5). The cyclic voltammogram of **4** was measured in acetonitrile, which revealed an oxidation event at −0.24 V vs Fc^+^/Fc couple (Figure S6), which is cathodically shifted compared to **1** (*E*_ox_ = −0.134 V vs Fc^+^/Fc couple).

### Synthesis and Characterization of Fe^IV^ Complexes

Next, we evaluated the reaction of Fe^III^ complexes with different oxidizing agents. We observed that the reaction of **1** with magic blue ((4–Br-C_6_H_4_)_3_NSbCl_6_) formed a ligand radical-coordinated Fe^III^(OH) complex (**2**),^33^ which was characterized by an array of spectroscopic techniques, whose reactivity studies were explored further.^33^ It has been shown before that the reaction of different Fe(II) or Fe(III) complexes with PhINTs resulted in the generation of Fe^IV^=NTs or Fe^V^=NTs complexes, respectively.^35^ Inspired by these studies, we envisioned that the reaction of **1** with PhINTs would also result in the formation of a ligand radical-coordinated Fe^IV^=NTs compound, which we thought based on our early observation of the occurrence of a ligand-derived oxidation event of **1**. Initially, to explore such chemistry, we set out to conduct the reaction of **1** with PhINTs.

**Figure 2:**
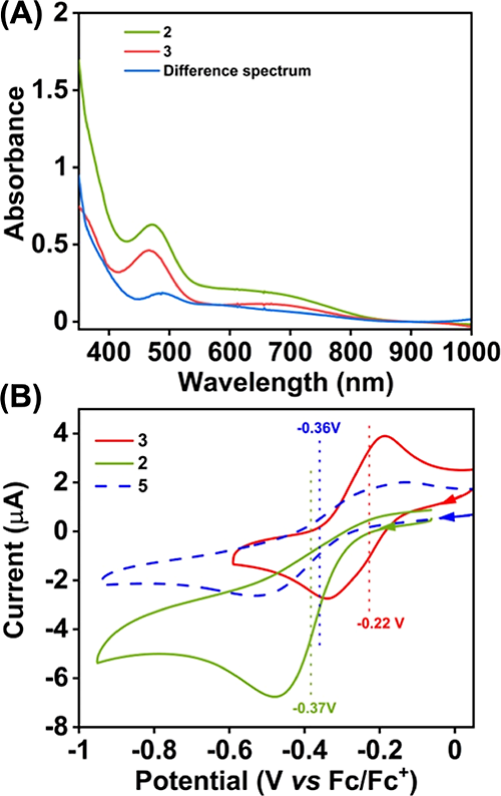
(A) UV–vis spectra of **2** (0.16 mM) and **3** (0.15 mM) measured in acetonitrile at −45 °C. (B) Cyclic voltammograms of **2** (0.5 mM), **3** (0.5 mM), and **5** (0.5 mM) were measured in acetonitrile at −15 °C. A glassy carbon working electrode, a Pt wire counter electrode, and ^*n*^Bu_4_NPF_6_ as the supporting electrolyte were used during the measurements.

**Scheme 2:**
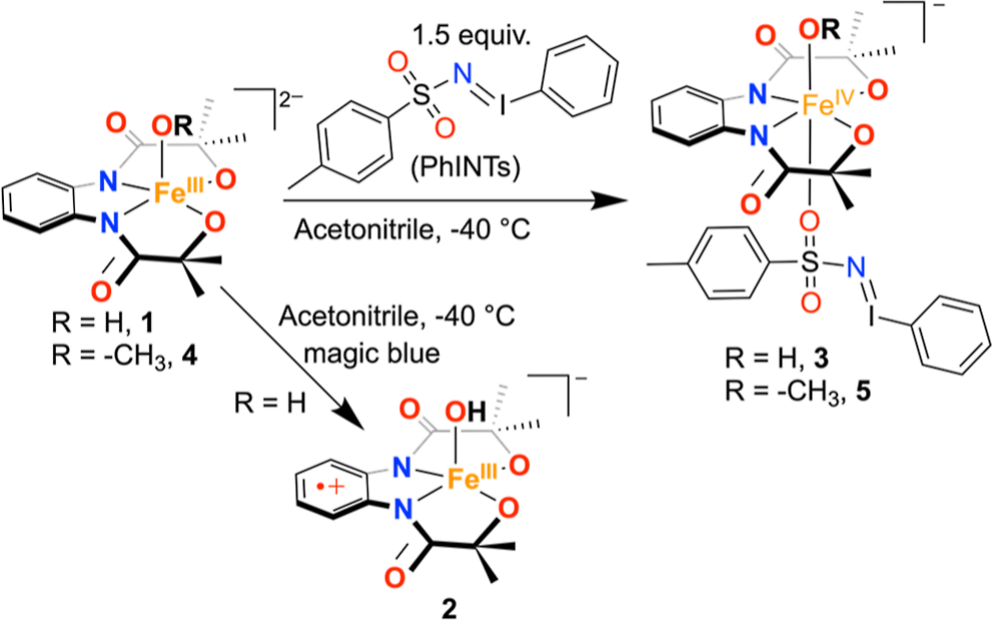
Reaction of **1** with Excess PhINTs and Magic Blue in Acetonitrile at −25 °C

We investigated the reaction of **1** with an excess amount of PhINTs (3 equiv with respect to the Fe complex) in acetonitrile at −25 °C and monitored the reaction by UV–vis spectroscopy. The formation of a new species **3** occurs upon adding PhINTs to **1** (Figures 2A and S7), which revealed absorbance maxima at 365 nm (3360 M^–1^ cm^–1^), 465 nm (3200 M^–1^ cm^–1^), and 680 nm (876 M^–1^ cm^–1^). Strikingly, the UV–vis features of **3** are very similar to species (**2**) obtained by adding magic blue to **1** (Figure 2A). To investigate the origin of the transitions in the oxidized Fe complexes, TD-DFT calculations were performed, as shown in Figure S7B. While **1** shows a featureless optical spectrum, **2** and **3** demonstrate 2 peaks in the visible region, which is consistent with experimental data. The calculated optical spectra of **2** and **3** are additionally very similar. A titration experiment was performed to understand the exact amounts of PhINTs needed to generate the intermediate **3** completely, which revealed no additional increase in absorbance maxima at 365 and 465 nm after the addition of more than ∼0.6 equiv of PhINTs (Figure S8) to **1**. The experiment infers that the one-electron oxidation of the Fe^III^(OH) complex (**1**) requires 0.5 equiv of PhINTs. However, in the presence of an excess of PhINTs in the solution, we speculate the coordination of PhINTs to the Fe center (trans to the Fe–OH, Scheme 2), which we suggest based on spectroscopic measurements and a drastic reactivity difference (vide infra). Further, species **3** remained EPR silent when the X-band EPR spectrum was measured in frozen acetonitrile at 77 K (Figure S9). The ^1^H NMR spectrum of **3** revealed paramagnetically shifted proton resonances (Figure S10). Measurement of solution magnetic moment of **3** by Evans’ method showed a μ_eff_ value of 4.73 μ_B_ (Figure S10), which corresponds to the presence of an *S* = 2 ground state of Fe in **3** and suggests that **3** is a one-electron oxidized species of **1**.

The electrochemical properties of **3** were additionally investigated in acetonitrile at ca. −15 °C in the presence of ^*n*^BuNPF_6_ as the supporting electrolyte. The one-electron reduction potential of **3** was observed at −0.22 V versus the Fc^+^/Fc couple, which is ca. 150 mV anodically shifted compared to the one-electron reduction potential of **2** at −15 °C, which was observed at −0.37 V vs Fc^+^/Fc (Figures 2B and S11). The results imply the presence of different coordination environments around Fe in **2** and **3**. The redox events observed in **2** and **3** can be assigned as ligand vs Fe-centered, respectively (vide infra).

**Figure 3:**
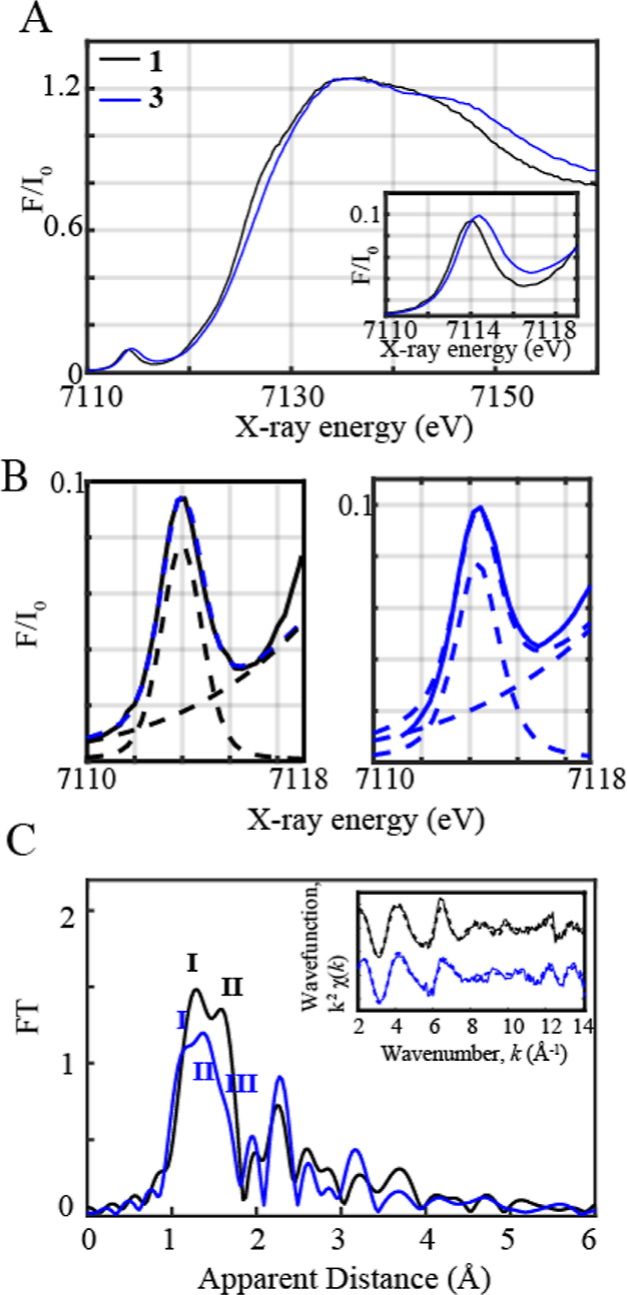
(A) Normalized Fe K-edge XANES spectra recorded at 20 K of **1** shown in black together with **3** (shown in blue). Inset: zoom-in view of the pre-edge regions of **1** and **3**. (B) Zoom-in view of the pre-edge regions together with the respective fits shown in dashed blue. The black and blue dashed lines correspond to the step and pseudovoigt functions used to fit the pre-edge peaks, respectively. (C) Fourier transforms of *k*^2^-weighted Fe EXAFS of **1** (in black) and **3** (blue). Inset: Back Fourier transformed experimental (solid lines) and fitted (dashed lines) *k*^2^χ(*k*) for **1** and **3**. Experimental spectra were calculated for *k* values of 2–14.107 Å^–1^.

Complexes **1** and **3** were subsequently investigated by X-ray absorption near-edge structure (XANES) and extended X-ray absorption fine structure (EXAFS) spectroscopy (Figure 3). Complex **3** generated with PhINTs displays a positive shift of the Fe–K edge energy of 0.95 eV from 7125.13 to 7126.08 eV at a normalized absorption of 0.6, reflecting the higher ionization energy required for ejecting a core 1s electron from a more positively charged Fe^IV^ ion.^36,37^ The observed edge energy of **3** is consistent with the reported Fe(IV) complexes.^38−40^ This is further corroborated by the observed upshift (∼0.35 eV) of the pre-edge energy transition at 7114.32 eV in **3** than 7113.97 eV in **1**. This pre-edge was observed for **2** at 7113.71 eV, which is lower in energy than **1**.^33^ The presence of pre-edge features corresponds to 1s to 3d quadrupole transitions and dipole excitations of the core electrons into the valence 3d states hybridized with ligand p orbitals.^41−43^ A pre-edge area of 19.3 units was obtained for **1** at 7113.97 eV, which is close and consistent with that of previously studied five-coordinated ferric centers, demonstrating pre-edge areas of ∼14.1 units at 7113 eV (Figure 3A inset, 3B, Table S3).^33^ By contrast, complex **3** demonstrates a pre-edge area of 23.5 units at 7114.32 eV (Figure 3A inset, 3B, Table S3) comparable to the reported high-spin Fe(IV) oxo complexes, where an area of ∼25 units has been observed.^38,40,44^ The lesser intense pre-edge feature of complex **3** vs iron(IV) oxo complexes of tetraamido macrocyclic ligands (TAMLs)^36,45,46^ is due to its higher coordination environment and more centrosymmetric geometry in comparison to five-coordinated Fe^IV^ complexes. Indeed, centrosymmetric complexes have been shown to have a decreased intensity in their pre-edge features due to an increase in the metal 4p mixing into the 3d orbitals, contributing toward the electric dipole 1s to 4p character of this transition.^41^ This effect was further corroborated through time-dependent density functional theory (TD-DFT) calculations (Figure S1). TD-DFT calculated five coordinated Fe^IV^=O and Fe^IV^–OH complexes of the HMPAB ligand display higher pre-edge intensities in comparison to the Fe^IV^ hydroxo complex bound to the oxygen atom of the PhINTs ligand in agreement with experimental pre-edge trends (Figures S1 and 3A inset) pointing toward a six-coordinated geometry in **3**. The increased coordination of complex **3** in comparison to the five-coordinated Fe^IV^(O) or Fe^IV^(OH) complexes was further proved by its EXAFS spectral data illustrated in Figure 3C.

The EXAFS spectra of the Fe(III) complex (**1**) display 2 peaks corresponding to the distinctive Fe–N and Fe–O bond distances, whereas the oxidized Fe(IV) complex (**3**) illustrates 2 peaks (**I**, **II**) at comparatively lower apparent distances corresponding to the shortened Fe–O/N bond distances together with a weak shoulder (**III**) arising from the bond between the Fe^IV^ metal center and oxygen of the PhINTs ligand (Scheme 2). EXAFS fits for the first coordination sphere and the entire spectrum for the Fe-based complexes are further shown in Table S4, Figure 3C inset, and Figure S12 in Supporting Information. In our previous study, we reported the EXAFS spectrum of the Fe(III) complex (**1**), which clearly resolves 3 Fe–O distances at 1.88 Å and 2 Fe–N distances at 2.01 Å, in close agreement with obtained XRD data (Table S4, Figure 3C, Supporting Information).^33^ By contrast, EXAFS fits of the Fe(IV) complex (**3**) show 3 shortened Fe–O bond distances at 1.82 Å (fit 9, Table S4), 2 Fe–N distances at 1.97 Å, and an elongated Fe–O bond distance at 2.13 Å. It is important to note here that the ligation of PhINTs to a Co(II) center through coordination of the O/N atom has been demonstrated before.^47^ The EXAFS data further reveals that the Fe–O_OH_ distance in **3** is ∼1.82 Å, which is significantly elongated than the reported Fe=O bond lengths of 1.64 Å in an Fe^IV^(O) complex with a TAML.^36^ Further, the calculated Fe^IV^(O) complex of HMPAB (Table S5, Supporting Information) as well as other examples illustrated Fe=O bond lengths < 1.7 Å.^38,40,48−51^ This excludes the possibility of the presence of a shortened Fe=O bond in **3**. Furthermore, a Fe=N distance of 1.65 ± 0.04 Å was obtained in the [Fe^V^(TAML)(NTs)]^−^ complex,^35^ which is also much shorter than the core bond distances observed in **3**. This comparison also discards the possibility of the formation of a Fe–imido backbone in **3**. Nonetheless, the Fe–O distance of **3** is close to the Fe–O bond length observed in the [Fe^IV^(ttpc)(OH)] complex (1.857(3) Å; H_3_ttpc = tris(2,4,6-triphenyl)phenyl corrole ligand)^21^ and the Fe–N distance reported in the [Fe^IV^(TAML)(NHTs)]^−^ species (1.89 Å).^30^

**Figure 4:**
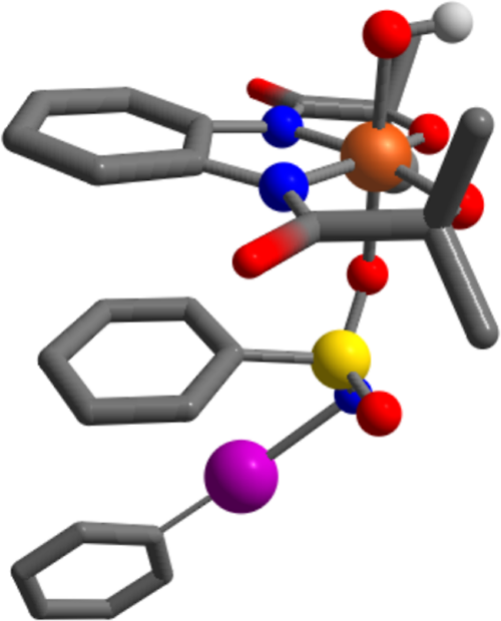
DFT-optimized structure of the Fe^IV^(OH) complex coordinated to PhINTs. During optimization, we excluded the methyl group of PhINTs.

The EXAFS data further agreed well with the DFT-calculated structure of **3** (vide supra). We investigated, in this case, the structure of **3** with *S* = 1 or 2 ground state with a six-coordinated geometry around Fe, where the sixth position is occupied by PhINTs through the O/N donor atoms (Appendix). While the optimized structure of Fe^IV^ coordinated by PhINTs through the N donor atom revealed a short Fe–N bond distance of 1.965 Å and a decreased calculated pre-edge intensity in comparison to **1** (Figure S1), the Fe^IV^–PhINTs complex bound to an O donor atom (*S* = 2 ground state) showed comparable pre-edge intensities (Figure 3A inset) in comparison to **1** as previously discussed. Furthermore, the optimized structure of **3** composed of an Fe^IV^(OH) complex with a coordinated O atom of PhINTs revealed an Fe–O_OH_ distance of 1.847 Å and an elongated Fe–O_PhINTs_ bond length of 2.11 Å (Figure 4) in agreement with experimentally obtained data (Table S5). It is important to remark that the DFT-optimized structure of an Fe^IV^(O) complex of HMPAB^4–^ revealed a Fe–O distance of 1.657 Å (Table S5), which is inconsistent with the EXAFS data of **3**, further showing that **3** is a six-coordinated Fe^IV^(OH) species with a bound PhINTs ligand. Thus, based on the experimental observations, we suggest here that the formation of intermediate **3** requires 1.5 equiv of PhINTs, 0.5 equiv of which is used to oxidize Fe(III) to Fe(IV), while another equiv bounds the Fe complex as an axial ligand to the Fe metal center.

Thus, the tetraanionic ligand scaffold (HMPAB^4–^) used in this study is not capable of forming Fe=NTs intermediates, which is in stark contrast to the [Fe^III^(TAML)]^−^ complex, where the formation of [Fe^V^(TAML)(NTs)]^−^ intermediate was observed.^35^ This sharp reactivity difference between these two ligand systems is noteworthy and reveals that the geometry of the ligand is crucial for the generation of Fe=X (X = NR or O) species. Nonetheless, stabilization of Mn^V^(O) species has been achieved by the use of a HMPAB^4–^ ligand scaffold.^52,53^

Next, we examined the reaction of the Fe^III^(OMe) complex (**4**) with PhINTs, which also resulted in similar spectral features to that of the reaction of **1** with PhINTs (Figure S13). By analogy with the reactivity of **1**, we presume the formation of a PhINTs-coordinated Fe^IV^(OMe) complex (**5**). Further, no peaks were observed in the X-band EPR spectrum of **5** at 77 K (Figure S14). The cyclic voltammogram of species **5** was then measured in acetonitrile at −15 °C using ^n^Bu_4_NPF_6_ as the supporting electrolyte, which revealed a reduction event at a half-wave potential of −0.36 V vs Fc^+^/Fc couple (Figure S15), which is cathodically shifted compared to **3**. Next, we determined the ^57^Fe Mössbauer spectrum of **5**, which is shown in Figure S16 at 77 K, and revealed contributions of two Fe^IV^ complexes, an *S* = 1 species with an isomer shift (δ) of −0.11 mm/s (Δ*E*_q_ = 0.77 mm/s) and an *S* = 2 species with an isomer shift (δ) of 0.17 mm/s (Δ*E*_q_ = 1.43 mm/s). Although we are unable to report here the Mössbauer spectrum of **3**, the data of an analogous compound (**5**) suggest the existence of Fe^IV^ in **3**, which is in corroboration with the XANES and EXAFS data.

Considering the different electronic structures of **3**, we set out to explore the reactivity studies of **3** and compare them with **2**. Additionally, we performed the reactivity studies of **5**.

### Hydroxide Rebound Study of **3**

**Figure 5:**
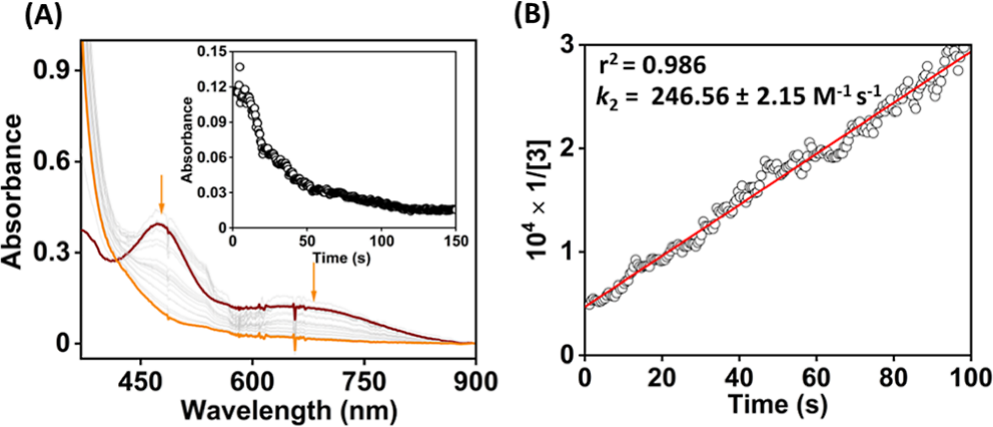
(A) Change in the UV–vis spectrum of **3** (0.2 mM) upon addition of 1 equiv of (4-OMe-C_6_H_4_)_3_C^•^ to an 1:4 acetonitrile/toluene solution (v/v) of **3** at −60 °C. (B) Plot of 1/[**3**] vs time for the determination of *k*_2_ value for the OH rebound reaction.

**Scheme 3:**
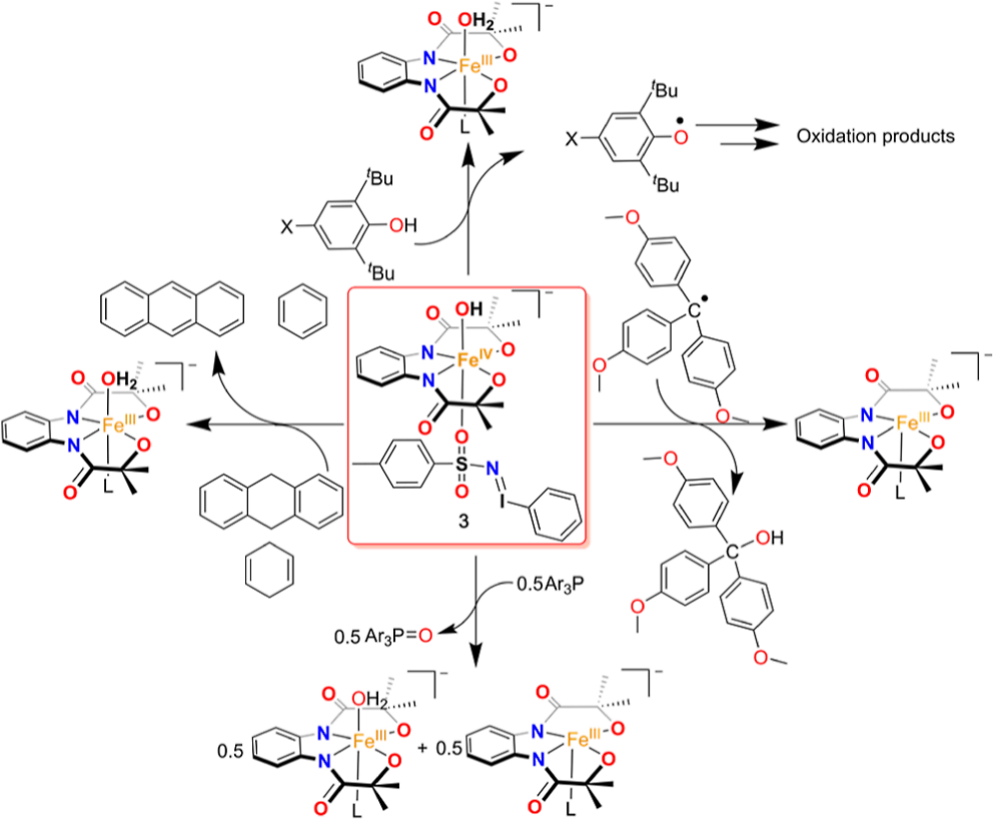
Reactivity Studies of **3**

We investigated the reaction of **3** with (4-OMe-C_6_H_4_)_3_C^•^ in 1:4 acetonitrile/toluene (v/v) at −60 °C (Figure 5, Scheme 3). The reaction was monitored by UV–vis spectroscopy, and a *k*_2_ value of (2.46 ± 0.02) × 10^2^ M^–1^ s^–1^ was estimated from the slope of a plot of 1/[**3**] vs time (s). Analysis of the reaction products by ^1^H NMR spectroscopy revealed the formation of 67% of (4-OMe-C_6_H_4_)_3_COH as the product (Figure S17). Thus, the rebound of the OH group of **3** to the carbon radical occurs spontaneously, which is a functional mimic of compound II of a large family of CYP. As the one-electron oxidation potential of (4-OMe-C_6_H_4_)_3_C^•^ is lower than the one-electron reduction potential of **3**, the initial ET from the radical to Fe^IV^ and subsequent attack by hydroxide is also another possibility for the formation of the C–OH bond, which we do not exclude.

Further, to compare the hydroxide rebound reaction of **3** with **2**, we investigated the reaction of **2** with (4-OMe-C_6_H_4_)_3_C^•^ in 1:4 acetonitrile/toluene (v/v) at −60 °C. Strikingly, no change of UV–vis spectral features was noticed over a period of 200 s (Figure S18). However, **2** reacted spontaneously with (4-OMe-C_6_H_4_)_3_C^•^ at a higher temperature. This reactivity difference between **3** and **2** suggests that the coordinated PhINTs trans to the OH group of Fe^IV^ in **3** enhances the reactivity of **3** than **2**.

Next, we examined the reaction of **5** with (4-OMe-C_6_H_4_)_3_C^•^ in 1:4 acetonitrile/toluene (v/v) at −25 °C. However, the formation of (4-OMe-C_6_H_4_)_3_C(OMe) was not observed in the reaction by GC–mass and ^1^H NMR spectroscopy studies. The experiment suggests that the Fe(IV)–OMe bond cleavage of **5** does not occur during the reaction of **5** with (4-OMe-C_6_H_4_)_3_C^•^.

### One-Electron Reduction Reactions

We subsequently examined the one-electron reduction reaction of **3** using decamethylferrocene (Fc*) as the reducing agent. The addition of 1 equiv of Fc* to an acetonitrile solution of **3** at −25 °C resulted in the decay of the intermediate with a *k*_et_ value of 7.8 × 10^2^ M^–1^ s^–1^ (Figure S19). The reaction resulted in the near quantitative formation of decamethylferrocenium cation (Fc*^+^), which was calculated by UV–vis spectroscopy. Nonetheless, when the reduction reaction of **2** was conducted in the presence of Fc* at −20 °C, a *k*_et_ value of 1.87 × 10^2^ M^–1^ s^–1^ was obtained (Figure S20). The slower reactivity of **2** compared to **3** illustrates the different electronic structure of **3** than **2**.

### Reactivity Studies of Fe Complexes with para-Substituted 2,6-Di-*tert*-butylphenols

**Figure 6:**
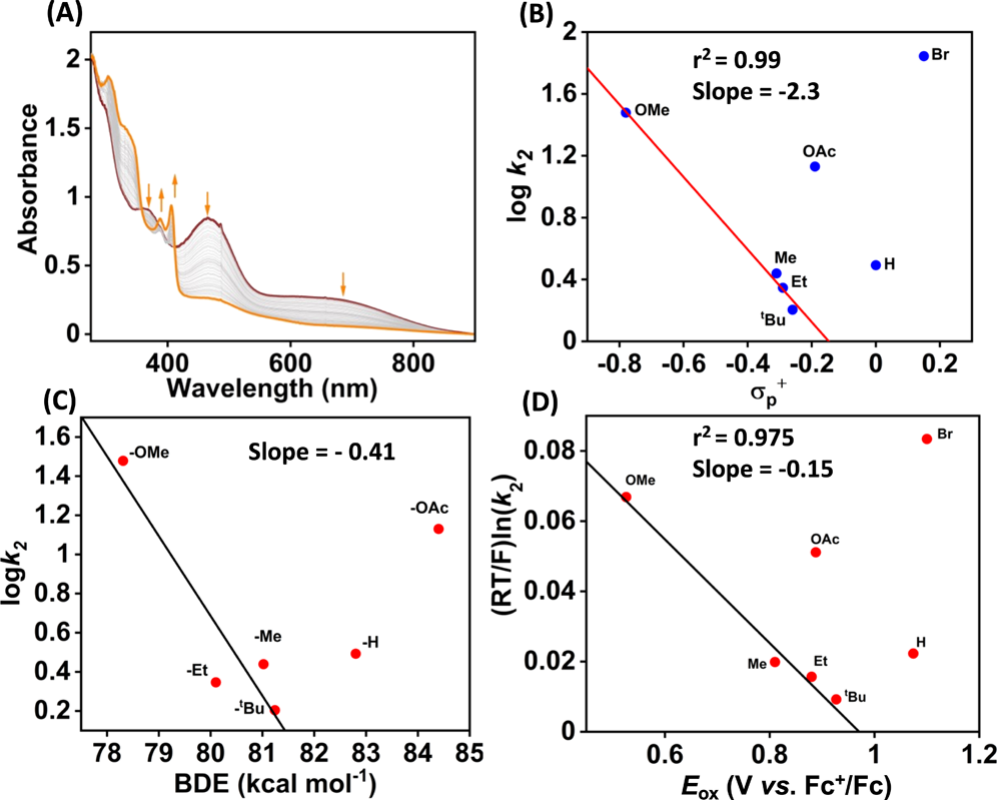
(A) Change in the UV–vis spectrum of **3** (0.25 mM) upon addition of 1 equiv of 4-OMe-DTBP in acetonitrile at −45 °C. (B) Plot of log *k*_2_ vs σ_p_^+^ of 4-X-DTBP substrates at −45 °C. (C) Plot of log *k*_2_ vs O–H BDE of 4-X-DTBP at −45 °C. (D) Plot of (*RT*/*F*) ln *k*_2_ vs *E*_ox_ of 4-X-DTBP substrates. *k*_2_ values were measured at −45 °C.

Next, we explored the reactivity of **3** with different 4-X-2,6-di-*tert*-butyl phenol (4-X-DTBP; X = OMe, Me, Et, ^*t*^Bu, H, Br, and OAc) substrates. The reaction of **3** with 4-X-DTBP substrates was performed in acetonitrile at −45 °C in the presence of substrates, and the reaction was monitored by UV–vis spectroscopy following the decay of the intermediate at 465 nm. The addition of 1 equiv of 4-OMe-DTBP to **3** resulted in the immediate decomposition of the intermediate and formation of the 4-methoxy-2,6-di-*tert*-butyl phenoxy radical at 390 and 406 nm in the UV–vis spectrum (Figure 6A). The formation of the isosbestic point was observed at 412 nm. Analysis of the reaction solution by EPR spectroscopy exhibited the formation of 52% of 4-methoxy-2,6-di-*tert*-butyl phenoxy radical in the reaction solution (Figure S21). Additionally, the analysis of the reaction solution by ^1^H NMR spectroscopy revealed the formation of 24% of 2,6-di-*tert*-butyl-1,4-benzoquinone as the 4-OMe-DTBP-derived reaction product (Figure S22). A *k*_2_ value of 30.1 ± 0.31 M^–1^ s^–1^ was obtained from the slope of a plot of 1/[**3**] vs time(s) for the reaction of **3** with 1 equiv of 4-OMe-DTBP (Figure S23).

To further understand the reaction of **3** toward the activation of the phenolic O–H bond, we performed the reaction of **3** with other 4-X-DTBP substrates (X = Me, Et, ^*t*^Bu, H, Br, and OAc) in acetonitrile at −45 °C in the presence of excess substrates (pseudo-first-order condition). Determination of *k*_2_ values and product analysis data for these substrates is described in Figures S24–S47. Tables S6 and S7 describe the observed *k*_2_ values and the reaction products, respectively. Plots of log *k*_2_ versus σ_p_^+^ (Hammett plot) and the O–H bond dissociation energy (BDE) of phenols of 4-X-DTBP substrates are shown in Figure 6. In each of the plots, a linear relation was observed from R = OMe to ^*t*^Bu. The trend discontinued from 4-H-DTBP, and no trend was observed with electron-deficient phenol substrates. Interestingly, we observed that the reactivity of **2** toward 4-H-DTBP falls in the same line as that of other electron-rich 4-X-DTBP substrates.^33^ A linear relationship in a plot of log*k*_2_ vs BDE is indicative of a rate-limiting O–H bond cleavage pathway, as reported for the phenol oxidation reactions of Mn–oxo or Ru–oxo species.^54,55^ Further, the results described in Figure 6 demonstrate a change in the reaction mechanism upon going from electron-rich to electron-deficient substrates, and such a changeover happens at 4-H-DTBP. The observation of C–C bond formation products in the case of electron-deficient phenol substrates infers the transfer of electron and proton to **3**. From thermodynamic consideration, the electron transfer from phenol to Fe^IV^ is not feasible because of the lower *E*_1/2_ value of **3**. We propose a rate-limiting proton transfer reaction followed by a fast electron transfer reaction that occurs in the case of electron-deficient phenol substrates. Such a reaction mechanism is also anticipated in the case of oxidation of electron-deficient phenols by a Mn(V)–imido complex.^56^

We further correlated the rate constants with the redox potential of 4-X-DTBP substrates. The observed *k*_2_ values increased upon decreasing the redox potential of the substrates, and the trend was disrupted again at 4-H-DTBP. The results imply that the redox-driving force controls the reaction in the electron-rich regime. A plot of (*RT*/*F*) ln *k*_2_ vs *E*_ox_ of the phenol derivatives showed a linear correlation, as displayed in Figure 6D, which revealed a negative slope of −0.15 with electron-rich substrates. No pattern was followed for other 4-X-DTBP derivatives when X = H, OAc, and Br, implying the occurrence of a different reaction mechanism. A slope of 0 in the Marcus plot corresponds to a pure hydrogen atom transfer (HAT) reaction. An example of this type of reaction is the reaction of the cumylperoxyl radical with 4-X-DTBP substrates, where a slope of −0.05 is reported.^57^ However, for rate-limiting electron transfer and fast proton transfer reactions, a slope of −0.5 is expected.^58^ Further, a slope of −1.0 should be obtained for rate-determining proton transfer and equilibrium electron transfer reactions.^58^ If the proton and electron transfer reactions happen at a comparable rate, then the slope value between −1.0 and −0.5 is expected,^59−61^ as reported for the reaction of phenol substrates with (μ–η^2^:η^2^-peroxo)dicopper(II)^62^ and Cu^III^(μ-O)_2_Ni^III^ complexes.^63^ The observed slope value (−0.15) in the present study is thus consistent with the occurrence of hydrogen atom transfer (HAT)/concerted proton–electron transfer (CPET) mechanism. Slopes of −0.19 and −0.12 have been reported for the HAT reaction of Fe^IV^(OH)(ttppc) and Mn^IV^(OH)(ttppc) species with 4-X-DTBP derivatives, respectively.^23^

We further compared the *k*_2_ values of 4-X-DTBP oxidation reactions of **2** with **3** at −25 °C, which are presented in Table 1 and Figures S48–S59. For all the 4-X-DTBP substrates, a much higher reactivity was observed with **3** compared to **2** (Figure 7).

**Table 1:** Comparison of *k*_2_ (M^–1^ s^–1^) Values for 4-X-DTBP Oxidation (−25 °C) with **2** and **3**

substrate	*k*_2_ (M^–1^ s^–1^) using **3**	*k*_2_ (M^–1^ s^–1^) using **2**
4-OMe-DTBP	190.1 ± 2.9	71.15^a^
4-Me-DTBP	69.8 ± 2.3	0.417 ± 0.01^a^
4-Et-DTBP	52.8 ± 1.7	0.416 ± 0.016^a^
4-^*t*^Bu-DTBP	42.2 ± 1.5	0.37 ± 0.009^a^
BNAH^b^	121.6 ± 2.7	65.02 ± 0.55
BNAD^b^	39.56 ± 0.34	31.04 ± 0.27
9,10-DHA^b^	(1.3 ± 0.05) × 10^–1^	
1,4-CHD^b^	(1.3 ± 0.03) × 10^–2^	

a Data was taken from ref (33).

b Data was recorded at −10 °C.

**Figure 7:**
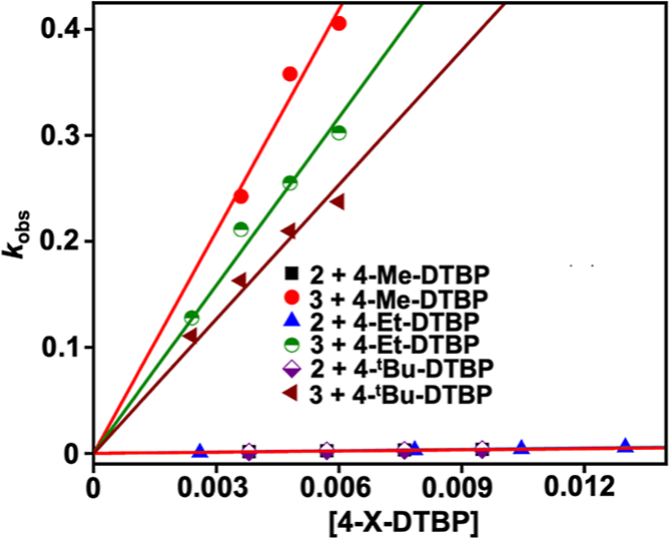
Plot of *k*_obs_ vs [4-X-DTBP] substrates (X = Me, Et, and ^*t*^Bu) for the reaction of **2** or **3** with 4-X-DTBP in acetonitrile at −25 °C. The *k*_obs_ values for the reaction of **2** with 4-X-DTBP were taken from ref (33).

For example, more than 120 times higher *k*_2_ of **3** with 4-Et-DTBP was obtained than **2** under identical reaction conditions. Likewise, 167 and 114 times faster reactions of **3** than **2** were noted when 4-Me-DTBP and 4-^*t*^Bu-DTBP were used as the substrates, respectively. Additionally, we compared the *k*_2_ values of **2** and **3** using 4-Et-DTBP at different temperatures (−25 to −45 °C), as displayed in Table S8. At all temperatures, a faster reactivity of **3** than **2** was noted; however, the ratio of *k*_2_(complex **3**)/*k*_2_(complex **2**) increased upon decreasing the temperature. Further, a very slow reactivity of **2** with 4-Br-DTBP was observed in acetonitrile. However, a very fast reaction was noted when **3** was used as the oxidant. Thus, a comparison of the PCET reactivity study clearly demonstrates that **3** is more oxidizing compared to **2**. Further, a plot of (*RT*/*F*) ln *k*_2_ vs *E*_ox_ of 4-X-DTBP substrates at −25 °C exhibited a slope of −0.076 (Figure S59), which indicates that the substrate redox potential dependence of **3** at higher temperatures is less and a HAT/CPET mechanism is favored. Examination of kinetic isotope effect (KIE) using 4-OMe-DTBP and **3** exhibited a *k*_2_^H^/*k*_2_^D^ value of 1.7. Nonetheless, the KIE value is significantly less for the phenol oxidation reaction following a HAT/CPET pathway. In addition, we also measured the *k*_2_ value of 2.27 M^–1^ s^–1^ for the reaction of **3** with 2,6-di-*tert*-butylphenol-*d* (Figures S38 and S39) and a KIE value of 1.36.

Then, we examined the preliminary reactivity studies of **5** with 4-Me-DTBP and 4-Et-DTBP in acetonitrile at −25 °C under pseudo-first-order reaction conditions. *k*_2_ values of 11.7 and 10.3 M^–1^ s^–1^ were obtained for 4-Me-DTBP and 4-Et-DTBP, respectively (Figures S60–S64), which are lower than the *k*_2_ values obtained for the reaction of **3** with these substrates. The slower reactivity of **5** than **3** can be correlated with the cathodically shifted *E*_red_ value of **5** relative to **3**.

### Reactivity Studies of Fe Complexes with Hydrocarbon Substrates

**Figure 8:**
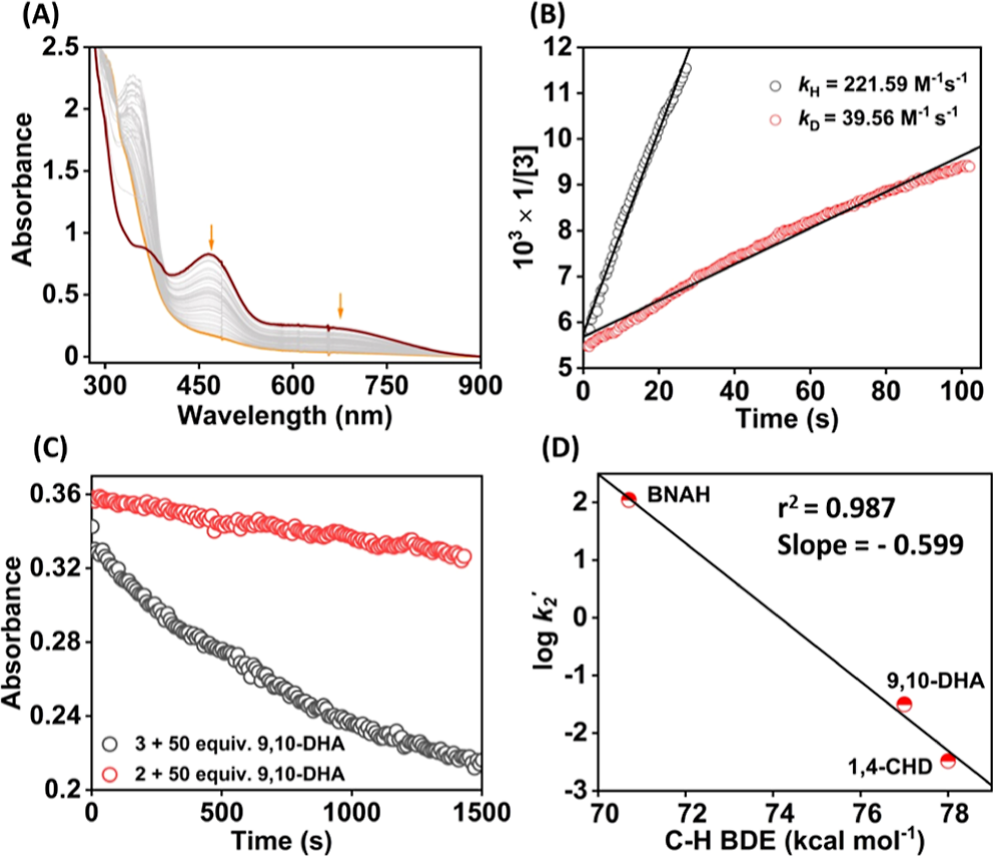
(A) Change in the UV–vis spectrum of **3** (0.2 mM) upon addition of 1 equiv of BNAH in acetonitrile at −10 °C. (B) Plot of 1/[**3**] vs time (s) for the reaction of **3** with BNAH or BNAD for the determination of *k*_2_ values. (C) Change in absorbance at 465 nm of **2**/**3** in the presence of an excess amount of 9,10-DHA at −10 °C. (D) Plot of log *k*_2_′ vs C–H bond dissociation energy of the substrates.

We further explored the hydrocarbon C–H bond activation reactions of **3** in acetonitrile at −10 °C (Scheme 3). An addition of 1 equiv of BNAH (1-benzyl-1,4-dihydronicotinamide) to an acetonitrile solution of **3** resulted in immediate decay of the intermediate, monitored by UV–vis spectroscopy (Figure 8A). The decay of **3** at 465 nm followed a second-order rate equation, and a *k*_2_ value of 221.6 ± 2.7 M^–1^ s^–1^ has been estimated from the slope of a plot of 1/[**3**] vs time (s) (Figure 8B). We observed that the reaction slowed down in the presence of deuterated BNAH, resulting in a *k*_2_^D^ value of 39.6 ± 0.3 M^–1^ s^–1^ and a primary KIE value of 5.6 (Figure 8B). Further, Eyring analysis was carried out to estimate activation parameters, which established Δ*H*^‡^ and Δ*S*^‡^ values of 9.4 kcal mol^–1^ and −11.8 cal K^–1^ mol^–1^, respectively (Figures S65 and S66, Table S9). The observed Δ*S*^‡^ value suggests the occurrence of a HAT/CPET pathway instead of a hydride transfer reaction. A Δ*S*^‡^ of ∼−20 cal K^–1^ mol^–1^ has been reported for the HAT reaction between M–oxo species and BNAH.^53,64^ However, in the case of involvement of the hydride transfer reaction, a more negative Δ*S*^‡^ is expected, as reported for the oxidation of 2-propanol.^65^ Additionally, **3** was found to react with substrates having relatively higher bond dissociation energy (BDE), such as 9,10-dihydroanthracene (9,10-DHA) and 1,4-cyclohexadiene (1,4-CHD) under pseudo-first-order reaction conditions with *k*_2_ values of 1.3 × 10^–1^ M^–1^ s^–1^ and 1.3 × 10^–2^ M^–1^ s^–1^ for 9,10-DHA (Figures S68–S70) and 1,4-CHD (Figures S72–S74) at −10 °C, respectively. However, ethylbenzene having a BDE higher than 80 kcal/mol was found to be unreactive toward **3** at −10 °C. The Brønsted–Evans–Polanyi (BEP) correlation plot revealed a coefficient (α) of −0.6 (Figure 8D, Table S10). Although the substrate scope is limited for the C–H abstraction reaction of **3**, similar plots have been reported for other high-valent metal complexes for HAT/CPET reactions.^6,66−68^ Thus, based on the BEP plot and observed KIE for BNAH, we speculate a CPET/HAT reaction mechanism for the C–H activation reactions by **3**.

Interestingly, we found that 9,10-DHA remained unreactive toward **2** in acetonitrile at −25 °C (Figure 8C), suggesting that **2** is a sluggish oxidant compared to **3**. However, **2** reacted with BNAH under second-order reaction conditions, resulting in a *k*_2_ value of 65.02 ± 0.55 M^–1^ s^–1^ (Figure S75) at −10 °C, considerably less than the *k*_2_ value of **3**. The reaction of **2** with BNAD (1-benzyl-1,4-dihydropyridine-4,4-*d*_2_-3-carboxamide) yielded a *k*_2_ value of 31.04 ± 0.27 M^–1^ s^–1^ (Figure S75). A primary KIE of 2.09 was obtained for the reaction of **2** with BNAH. Thus, a comparison of reactivity studies with BNAH and 9,10-DHA revealed different electronic structures of **2** and **3**, showing that **3** is a better oxidant than **2**.

### Reactivity Studies of Fe Complexes with Triarylphosphine Derivatives

**Figure 9:**
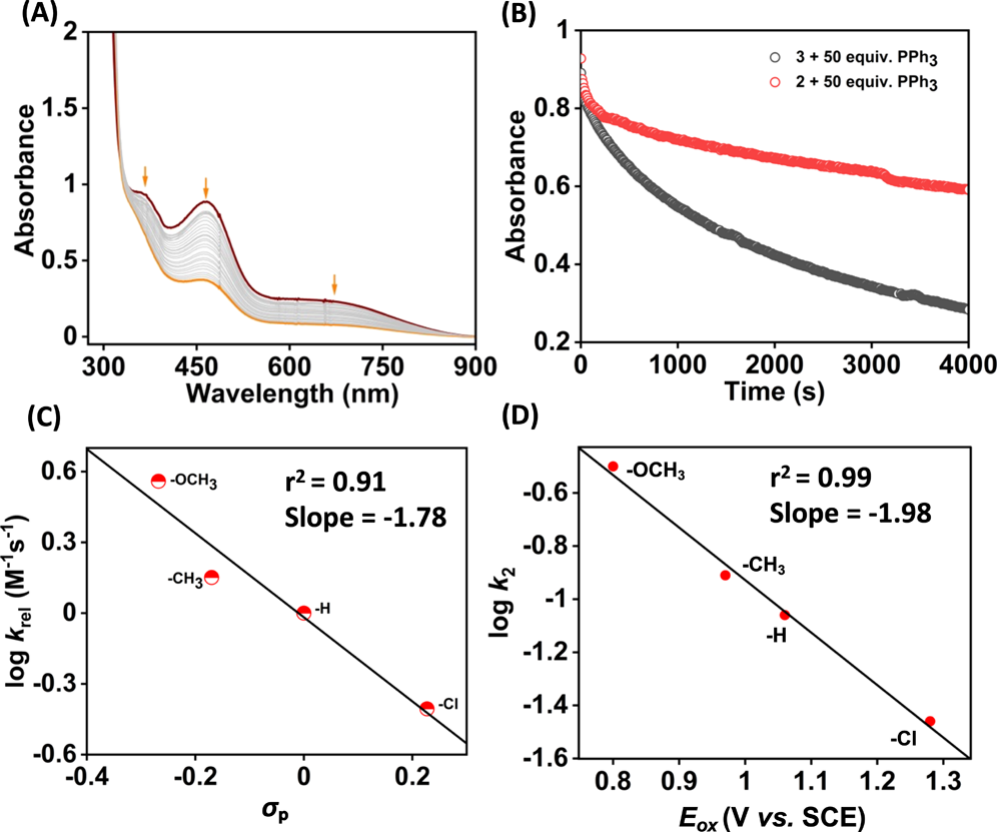
(A) Change in the UV–vis spectrum of **3** upon addition of 50 equiv of PPh_3_ to **3** in acetonitrile at −10 °C. (B) Change in absorbance at 465 nm of **2**/**3** in the presence of 50 equiv of PPh_3_ at −10 °C. Plots of log *k*_rel_ vs σ_p_ (C) and log *k*_2_ vs *E*_ox_ of (4-X-C_6_H_4_)_3_P (D) for the reaction of **3** with (4-X-C_6_H_4_)_3_P at −10 °C.

Reactivity studies of high-valent M–OH/M–OH_2_ species toward OAT reactions are rarely reported in the literature.^69^ Thus, we set out to investigate the reaction of **3** with *tris*(4-X-phenyl)phosphine derivatives (X = OMe, Me, H, and Cl). The addition of a 50-fold excess of Ph_3_P to an acetonitrile solution of **3** at −10 °C resulted in the decay of the features of the intermediate (Figure 9). Analysis of reaction products by ESI-mass spectrometry and ^31^P NMR spectroscopy revealed the formation of 25% of Ph_3_P=O as the product (Figure S76). Likewise, we observed the formation of 44% of (4-OMe-C_6_H_4_)_3_P=O when (4-OMe-C_6_H_4_)_3_P was used as the substrate (Figure S77). The product yields imply that 2 equiv of oxidant is required to convert 1 equiv of Ar_3_P to Ar_3_PO. Analysis of the reaction solution after the reaction of **3** with Ph_3_P by EPR spectroscopy revealed the formation of Fe(III) complexes. The observation of Fe(III) species in the reaction solution demonstrates that 2 equiv of **3** is required to oxidize 1 equiv of PPh_3_. Thus, the reaction of **3** with Ar_3_P substrates showed that a Fe^IV^(OH) complex is capable of participating in the OAT reactions. An ^18^O-labeling experiment was additionally conducted using **3** and (4-OMe-C_6_H_4_)_3_P in the presence of added H_2_O, revealing the incorporation of ∼90% of ^18^O in the formed (4-OMe-C_6_H_4_)_3_PO and inferring that the OH group of **3** is exchangeable (Figure S78). Interestingly, no nitrogen group transfer reaction product to the Ar_3_P substrates was noted in the ^31^P NMR and ESI-mass data, implying that the weakly coordinated PhINTs to Fe^IV^(OH) are not capable of forming Ar_3_P=NTs-type products.

We subsequently explored the kinetics of the reaction of Ar_3_P substrates with **3** in acetonitrile at −10 °C (Figures S79–S89). The *k*_obs_ values were found to increase linearly with increasing concentrations of substrates (Figures S80, S83, S86, and S89). However, a *y*-axis intercept was observed in all of the *k*_obs_ vs [Ar_3_P] plots, suggesting that a side reaction pathway is operating in the presence of Ar_3_P substrates. The *k*_2_ values for all substrates are listed in Table S11. A plot of log *k*_rel_ vs σ_p_^+^ yielded a linear correlation plot with a slope of −1.78 (Figure 9C), corroborating the electrophilic nature of the Fe^IV^(OH) intermediate species. Further, a plot of log *k*_2_ vs *E*_ox_ of the Ar_3_P substrates yielded a linear correlation and revealed a decrease in reactivity with an increase in the oxidation potential of Ar_3_P substrates (Figure 9D), corroborating that **3** is working as an electrophile in the oxidation of Ar_3_P substrates.

However, a very slow reaction was observed when the reaction of **2** was conducted with an excess of PPh_3_ (50-fold excess) at −10 °C (Figure 9B), and we were unable to obtain rate constant values.

The OAT studies further established an enhanced reactivity of **3** than **2** and also showed for the first time that a Fe^IV^(OH) species is capable of participating in the OAT reaction.

## Conclusions

The present study demonstrates the reaction of a Fe^III^(OH) complex (**1**) with excess PhINTs, which resulted in the formation of a Fe^IV^(OH) complex (**3**) with coordinated PhINTs at the axial position. However, the reaction of **1** with one electron-oxidizing agent was shown to generate a ligand radical-coordinated Fe^III^(OH) species (**2**).^33^ We suggest that the coordination of PhINTs to Fe causes the metal-based electron removal rather than ligand oxidation in **1**. The different coordination geometry around Fe in **2** and **3** is supported by cyclic voltammetry studies: the one-electron reduction potential of **3** is 150 mV anodically shifted than **2**, which is because of the presence of an additional ligand (PhINTs) around Fe in **3**. Further, the X-ray absorption spectroscopic investigations of **3** support the + IV oxidation state and an octahedral geometry around Fe in **3**. The Fe–OH distance in **3** was found to be ∼1.84 Å by the EXAFS technique, which is considerably elongated compared to the Fe–O distance expected in a Fe^IV^=O species.^70,71^ It is important to remark here that the Fe–O bond lengths of Cpd-II in chloroperoxidase^10^ and P450 (CYP158-II)^11^ were found to be 1.82 and 1.84 Å, respectively. We also present the synthesis and characterization of a Fe^III^(OMe) complex (**5**). The reaction of this complex with PhINTs resulted in the generation of a Fe^IV^(OMe) complex (**5**), where the coordination of PhINTs to the Fe center has been suggested. Mössbauer data of the latter complex supports the formation of the + IV Fe metal center in **5**. Electrochemical measurements revealed that one-electron reduction potential of **5** is cathodically shifted than **3**.

The reactivity of Fe^IV^(OH) species (**3**) was compared with the ligand radical-coordinated Fe^III^(OH) complex (**2**) toward PCET and OAT reactions. We suggest that the observed higher reactivity of **3** than **2** is because of the coordinated PhINTs at the sixth position in **3**. In addition, the reactivity studies of **5** exhibited a slower reaction compared to **3**. Overall, the study describes the detailed OH rebound, PCET, OAT, and ET reaction studies of high-valent Fe–OH complexes and highlights the importance of the electronic structure of Fe in controlling the reactivity.

## Methods

The chemicals and solvents used in this study were purchased from commercial sources and used as received unless mentioned. The iron(III) complexes used in this study were prepared inside a N_2_-filled glovebox. (4-OMe-C_6_H_4_)_3_C^•^,^21^ 2,6-di-*tert*-butyl-4-methoxyphenol-*d*,^57^ BNAH,^72^ and BNAD^73^ were prepared following literature procedures. We recently described the synthesis and characterization of Fe^III^(OH) (**1**) and ligand-oxidized Fe^III^(OH) (**2**) complexes.^33^^1^H NMR spectra of organic molecules and Fe complexes were recorded on a Bruker 500 MHz (DPX-500) or Bruker 400 MHz (DPX-400) NMR spectrometer. The ESI-mass data reported in this study were measured using a Waters Xevo-G2XQTOF instrument. The IR spectrum of the Fe complexes was measured in KBr pellets using a Nicolet Protégé 460 ESP instrument. CHN analysis of all Fe complexes was recorded in a PerkinElmer 2400 series II CHNS/O instrument. UV–vis spectra of Fe complexes were measured using an Agilent diode array 8454 spectrophotometer connected to a Unisoku cryostat. Mössbauer data of intermediate **5** was recorded using a ^57^Co source in a Rh matrix in an alternating constant acceleration Wissel Mössbauer spectrometer operated in the transmission mode using the Janis Research SuperVariTemp setup. Isomer shifts were reported relative to the iron foil at ambient temperature. Simulation of experimental data was done using Igor Pro 8 software.

*Caution*: Although no problems were encountered during the synthesis of the complex, perchlorate salts are potentially explosive and should be handled with care!^74^

### Synthesis of (NMe_4_)_2_[Fe^III^(HMPAB)(OMe)] (**4**)

A methanolic solution (3 mL) of FeCl_3_ (0.08 g, 0.5 mmol) was added dropwise to a stirring reaction solution of H_4_HMPAB (0.15 g, 0.5 mmol) and Me_4_NOH (0.85 g, 25% solution in methanol; 2.25 mmol, 4.5 equiv) in methanol (2 mL) inside a nitrogen-filled glovebox. The resulting reaction solution was allowed to stir at around 25 °C for 2 h. Then, the solvent was reduced to dryness, and acetonitrile (ca. 3 mL) was added to the residue to dissolve. An excess of diethyl ether was slowly introduced into the reaction solution and was allowed to stir at room temperature. Then, the reaction mixture was placed at −20 °C inside a refrigerator overnight. Precipitation of a yellowish-brown solid occurred. The solid compound was separated and dried under vacuum. Single crystals suitable for X-ray diffraction were obtained upon diffusing diethyl ether into an acetonitrile solution of the complex at room temperature. Yield: 0.11 g (42%). Anal. Calcd for **4**·H_2_O (C_23_H_43_FeN_4_O_5_·H_2_O: 529.48 g/mol): C, 52.17; H, 8.57; N, 10.58. Found: C, 52.46; H, 8.83; N, 10.32. FT-IR (cm^–1^): 560 (m), 602 (m), 653 (m), 772 (w), 950 (s), 1033 (w), 1166 (s), 1242 (w), 1398 (s), 1451 (m), 1484 (s), 1542 (vs), 1591 (s), 1658 (m), 2973 (w), 3017 (m), 3399 (br). UV–vis (in acetonitrile, nm): 485 (br), 360 (br). X-band EPR (in methanol/THF, g values): 5.9 and 2.0.

Approximately 50% of the ^57^Fe-enriched (NMe_4_)_2_[Fe^III^(HMPAB)(OMe)] complex was prepared following a similar procedure as described above. The ^57^Fe isotope-enriched FeCl_3_ was prepared by mixing 1:1 naturally abundant Fe and ^57^Fe metal and refluxed for 24 h with aqueous HCl under air.

### Product Analysis

10 mL of acetonitrile was added to complex **1** (3.7 mg, 0.0072 mmol) in a reaction bath (RB) inside a nitrogen-filled glovebox, and the RB was sealed with a septum. The reaction solution was then placed in an Eyla low-temperature reaction bath precooled to −10 °C. An acetonitrile solution (1 mL) of PhINTs (4 mg, 0.0108 mmol) was slowly introduced into the reaction solution and was allowed to stir at −10 °C for 5–7 min for generation of the intermediate species (**3**). Then, different substrates were introduced into the reaction solution slowly using a syringe maintaining the N_2_ atmosphere and allowed to stir at −10 °C (described below for different substrates).

### Reaction of **3** with (4-OMe-C_6_H_4_)_3_C^•^

(4-OMe-C_6_H_4_)_3_C^•^ (0.0072 mmol, prepared in situ) dissolved in 1 mL of 1:4 acetonitrile/toluene solution (v/v) was slowly introduced into an acetonitrile solution (10 mL) of **3** (0.0072 mmol) at −10 °C. The reaction mixture was allowed to stir for 25–30 min, maintaining the temperature at −10 °C. Then, the reaction mixture was warmed to room temperature, and the solvent was removed under high vacuum. The resulting residue was redissolved in CDCl_3_, and ^1^H NMR data of the crude reaction mixture was recorded without further purification. (4-OMe-C_6_H_4_)_3_C–OH was quantified using trimethoxybenzene as an internal standard.

A blank experiment was also performed in the absence of intermediate **3** under the same experimental conditions, which revealed a trace amount of (4-OMe-C_6_H_4_)_3_C–OH.

### Reaction of **3** with BNAH

An acetonitrile solution of BNAH (0.0072 mmol) was slowly introduced into the reaction solution containing intermediate **3** (0.0072 mmol) and allowed to stir for 10 min at −10 °C. Then, the reaction mixture was quenched with a minimum amount of dilute HCl (in acetonitrile), and all solvent was removed under high vacuum. The resultant residue was dissolved in D_2_O, and the ^1^H NMR and ESI-mass spectra of the crude reaction mixture were recorded without further purification. Quantification of BNA^+^ was performed by ^1^H NMR spectroscopy using 3,5-dinitrobenzoic acid as an internal standard. The formation of BNA^+^ (∼96%) was noted.

A blank experiment was performed under similar experimental conditions in the absence of **3**. The experiment revealed no formation of BNA^+^ as the product.

### Reaction of **3** with (4-X-C_6_H_4_)_3_P Substrates (X = H, OMe, Me, and Cl)

An acetonitrile solution of (4-X-C_6_H_4_)_3_P (0.0072 mmol) in 1 mL of acetonitrile was introduced into the stirring reaction solution containing **3** (0.0072 mmol) and allowed to stir at −10 °C for 5 h. Then, the solvent was removed under reduced pressure, and the resulting crude residue was redissolved in CDCl_3_ and analyzed by ^31^P NMR spectroscopy and ESI-mass spectrometry. The formed product was quantified by NMR spectroscopy by comparing the integration of (4-X-C_6_H_4_)_3_P=O with unreacted (4-X-C_6_H_4_)_3_P.

A blank experiment was also performed under the same reaction conditions in the absence of **3**, which showed no formation of the (4-X-C_6_H_4_)_3_P=O product. The yields of different (4-X-C_6_H_4_)_3_P-derived products are listed in Table S7.

### Reaction of **3** with 4-X-DTBP

An acetonitrile solution (1 mL) of 4-X-2,6-DTBP (0.012 mmol) was slowly introduced into an acetonitrile solution of intermediate **3** (0.012 mmol) at −25 °C under the N_2_ atmosphere. The resulting reaction solution was allowed to stir at −25 °C for 1 h maintaining the N_2_ atmosphere. Once the reaction was complete, the solution was quenched with a minimum amount of dilute HCl (in acetonitrile), and the solvent was removed under reduced pressure. The organic products were extracted with diethyl ether (3× 20 mL), dried over anhydrous sodium sulfate, and evaporated to dryness. The crude product was analyzed by ^1^H NMR spectroscopy, and the 4-X-DTBP-derived products were quantified by comparing their integration value with the unreacted substrate. The yields of different 4-X-DTBP-derived products are listed in Table S7.

A blank experiment was also performed in the absence of **3**, which revealed that no oxidized products were formed.

### Reaction of **3** with 9,10-DHA

An acetonitrile solution (1 mL) of 9,10-dihydroanthracene (0.12 mmol) in 1 mL of acetonitrile was slowly introduced into an acetonitrile solution (10 mL) of **3** (0.012 mmol) at −10 °C under a N_2_ atmosphere. The resulting reaction solution was allowed to stir at −10 °C for 5 h. Once the reaction was complete, the solution was quenched with a minimum amount of dilute HCl (in acetonitrile), and the solvent was removed under reduced pressure. As an internal standard, 1 equiv (0.12 mmol) trimethoxybenzene was added to the reaction mixture. Then, the organic products were extracted with diethyl ether (3× 20 mL) as the solvent, dried over anhydrous sodium sulfate, and evaporated to dryness. The organic products were analyzed and quantified by ^1^H NMR without further purification. The formation of dihydroanthracene (∼50%) was noted.

## Supporting Information Available

Crystallographic data (CIF)
Detailed experimental procedures; NMR, ESI-mass, IR, UV–vis, and kinetic studies; and details of computational studies (PDF)

## Supplementary Material

au3c00844_si_001.cif
