# Supplementary material for: Functional Model of Compound II of Cytochrome P450: Spectroscopic Characterization and Reactivity Studies of a FeIV–OH Complex
Source: JACS Au. 2024 Mar 11;4(3):1142–54. doi: 10.1021/jacsau.3c00844 (PMC10976569; doi:10.1021/jacsau.3c00844)
Supplement: Supplementary file 2 — au3c00844_si_002.pdf [file au3c00844_si_002.pdf]

Supporting Information for

Functional Model of Compound II of Cytochrome P450: Spectroscopic Characterization and Reactivity Studies of a Fe^{IV}–OH Complex

Kritika Keshari,[a] Aakash Santra,[a] Lucía Velasco,[b] Maxime Sauvan,[b] Simarjeet Kaur,[a] Ashok D. Ugale[b], Sandip Munshi,[c] J. F. Marco[d], Dooshaye Moonshiram,[b]* Sayantan Paria[a]*

[a]Department of Chemistry, Indian Institute of Technology Delhi, Hauz Khas, New Delhi 110016; India, Email: sparia@chemistry.iitd.ac.in

[b]Instituto de Ciencia de Materiales de Madrid, Consejo Superior de Investigaciones Científicas, Sor Juana Inés de la Cruz, 3, 28049, Madrid, Spain; Email: dooshaye.moonshiram@csic.es

[c]School of Chemical Science, Indian Association for the Cultivation of Science, Raja S C Mulliick Road, Kolkata 700032, India

[d]Instituto de Quimica Fisica Blas Cabrera, Consejo Superior de Investigaciones Científicas, C. de Serrano, 119, Chamartin, 28006, Madrid, Spain

Determination of solution magnetic moment of the Fe complexes. The solution magnetic moment of the Fe^{IV}(OH) complex (**3**) was determined using Evans' method.[1-2] A 8.48 mM of a 640 μL solution of **3** in CD_3CN containing HMDS (internal standard) was prepared at –20 °C inside a Wilmad screw-cap NMR tube. A CD_3CN solution of HMDS in a Wilmad coaxial insert stem was then slowly inserted inside the screw-cap NMR tube. The ^{1}H-NMR spectra of **3** was then recorded in a Bruker 500 MHz NMR instrument at –10 °C. Paramagnetic susceptibility of **3** was measured using the following equation[1]:

$\chi_P = \chi_0 + 3000\Delta\nu/4\pi\nu_0 cM$

Here, χ_0 = diamagnetic susceptibility, $\Delta\nu$ = shift of frequency of the methyl protons of HMDS in Hz, ν_0 = frequency of the NMR instrument used during the measurement, c = concentration of the Fe complex, and M = molecular weight.

Effective magnetic moment (μ_{eff}) of the Fe complexes were determined using the following equation[2]:

$\mu_{eff} = (3k_B\chi_P T/N_A\beta^2)^{1/2} = (8 \times \chi_P \times T)^{1/2}$

Where, k_B = Boltzmann's constant, T = Temperature, N_A = Avogadro's number, β = Bohr magneton. The ratio of $3k_B /N_A\beta^2 \approx 8$.

Diamagnetic corrections of **3** were done according to the literature procedure to estimate the paramagnetic susceptibility value. Molar paramagnetic susceptibility (χ_m) was estimated from the χ_P value and molecular weight of **3**.

$\Delta\nu$ of 180 Hz was observed experimentally. The molecular weight of **3** = 839.6 g/mol, the concentration of **3** = 8.48 mM, and ν_0 = 500.6 MHz were used for the calculation of χ_P.

Kinetic Studies. Kinetic studies reported in this study were performed in an Agilent Cary 8454 spectrophotometer connected to a liquid nitrogen-controlled UNISOKU cryostat. In a typical experiment, 2.5–3 mL of a 0.15-0.32 mM solution of **1** was taken in a long neck cuvette and placed inside the cryostat at a desired temperature (–10 to –45 °C) and allowed to keep for five minutes. A 50 μL suspension of PhINTs (approx. 3 equiv. with respect to **1**) in acetonitrile was introduced in the cuvette using a Hamilton syringe connected to a long neck needle. After the complete formation of the intermediate species **3** (monitored at 465 nm in the UV-vis spectrum), substrate solution in acetonitrile (1-100 equiv, 50–100 μL solution) was introduced into the reaction solution, and the decay of the intermediate was monitored by UV-vis spectroscopy at 465 nm. Pseudo-first-order rate constants (k_{obs}) were estimated from the slope of a plot of ln(A–A$_\infty$) vs. time(s). The second-order rate constants (k_2) were determined from the slope of a plot of k_{obs} vs. [substrate]. For the reactions studied in the presence of one equiv. of the substrate, k_2 values were estimated from the slope of a plot of 1/[complex] vs. time (s). Likewise, the kinetic studies using the complex **5** were done.

X-ray Structure Determination. Single crystals of Fe^{III}(OMe) Complex (**4**), suitable for X-ray diffraction, were obtained by diffusing diethyl ether into the acetonitrile solution of the complex at room temperature. Crystal data was measured on a Bruker D8 VENTURE Microfocus diffractometer system equipped with a PHOTON II Detector, with Mo K_{α} radiation (λ = 0.71073 Å) and controlled by the APEX4 (v2022.1−1) software package. The raw data were integrated and corrected for Lorentz and polarization effects with the aid of the Bruker APEX4 program suite. Absorption corrections were performed by using SADABS. Structures were solved by the intrinsic phasing method and refined against all data in the reported 2θ ranges by the full-matrix least squares method based on F^2 using the SHELXL program suite[3] with all observed reflections. Hydrogen atoms at idealized positions were included in the final refinements. The non-hydrogen atoms were treated anisotropically. Diagrams for the complexes were prepared using Mercury software.[4] Crystallographic data of Fe complex is given in Table S1, and bond parameters are mentioned in Table S2.

Table S1. Crystallographic parameters of **4**.

Identification code	SP300922B_0m_a
Empirical formula	$C_{23}H_{47}ClFeN_4O_7$
Formula weight	547.49
Temperature/K	100(2)
Crystal system	monoclinic
Space group	$P2_1/c$
a/Å	13.8745(11)
b/Å	10.9020(9)
c/Å	19.4072(15)
α/°	90
β/°	104.391(3)
γ/°	90
Volume/Å^3	2843.4(4)
Z	4
ρ_{calc}g/cm^3	1.279
μ/mm^{-1}	0.575
F(000)	1180.0
Radiation	MoKα (λ = 0.71073)
2Θ range for data collection/°	4.318 to 50.144
Index ranges	$-16 \leq h \leq 16$, $-12 \leq k \leq 12$, $-23 \leq l \leq 23$
Reflections collected	39454
Independent reflections	5017 [R_{int} = 0.1229, R_{sigma} = 0.0892]
Data/restraints/parameters	5017/279/345
Goodness-of-fit on F^2	1.014

Final R indexes [I>=2σ (I)]	R_1 = 0.0588, wR_2 = 0.1397
Final R indexes [all data]	R_1 = 0.0877, wR_2 = 0.1541
Largest diff. peak/hole / e Å^{-3}	0.63/-0.48

Table S2. Selected Bond Length and Bond Angles for **4**.

Bond Length (Å)/Bond Angle (°)	**4**	Bond Length (Å)/Bond Angle (°)	**4**
Fe(1)–O(1)	1.889(3)	O(1)–Fe(1)–N(2)	108.05(12) .
Fe(1)–O(2)	1.912(3)	O(2)–Fe(1)–N(2)	140.34(12)
Fe(1)–O(3)	1.914(3)	O(3)–Fe(1)–N(2)	80.30(11)
Fe(1)–N(1)	2.059(3)	O(1)–Fe(1)–N(1)	102.20(12)
Fe(1)–N(2)	2.056(3)	O(2)–Fe(1)–N(1)	80.20(11)
O(1)–Fe(1)–O(2)	107.55(11)	O(3)–Fe(1)–N(1)	146.15(12)
O(1)–Fe(1)–O(3)	107.81(11)	N(2)–Fe(1)–N(1)	75.77(11)
O(2)–Fe(1)–O(3)	104.71(11)		

X-ray Absorption Near Edge (XANES) and Extended X-ray Absorption Fine Structure (EXAFS) Measurements

X-ray absorption spectra on the Fe(III) and Fe(IV) complexes were carried out at the Petra P64 beamline[5] (Hamburg, Germany) at electron energy 7.1 KeV and average current 100 mA. The radiation was monochromatized by a Si(111) crystal monochromator. The intensity of the X-ray was monitored by three ion chambers (I_0, I_1 and I_2) filled with 70% nitrogen and 30% helium and placed before the sample (I_0) and after the sample (I_1 and I_2). A Fe metal foil was placed between the I_1 and I_2 and its absorption recorded with each scan for energy calibration. Plastic (PEEK) EXAFS sample holders (inner dimensions of 12 m x 3 mm x 3mm) filled with the frozen solutions were inserted into a pre-cooled (20 K) cryostat and kept in a He atmosphere at ambient pressure. The XAS data was in this case recorded as fluorescence excitation spectra using a 4-element silicon drift detector. The Fe XAS energy was calibrated by the first maximum of the second derivative of the Fe metal XANES spectrum. A total of 8-10 scans were collected for the Fe(III) and Fe(IV)-based complexes In order to reduce the risk of sample damage by X-ray radiation, no more than 2 scans were taken at each sample position in any conditions. No radiation damage was observed to any of the two samples scan after scan. The XAS data were further recollected at Diamond Light Source at bending magnet beamline BM-18 at a storage-ring electron energy of 7 GeV and average current of 100 mA. The radiation was also monochromatized by a Si(111) crystal monochromator, and the XAS data were recorded in fluorescence mode in a continuous He flow cryostat. Around 15 XAS spectra were collected in this case for the Fe(III) and Fe(IV)-based complexes. Care was also taken to measure at several sample positions on each sample (beam size 1000 µm (Horizontal)

× 1000 μm(Vertical)) and no more than 5 scans were taken at each sample position. No damage was observed scan after scan to any samples.

EXAFS Data Analysis

Athena software[6] was used for data processing. The energy scale for each scan is normalized using the iron metal standard, and scans made for the same samples were added. Data in energy space are pre-edge corrected, normalized, and background corrected. The processed data are next converted to the photoelectron wave vector (*k*) space and weighted by k^2. The electron wave number is defined as $k = [2m(E - E_0)/\hbar^2]^{1/2}$, E_0 is the energy origin or the threshold energy. k-space data were truncated near the zero crossings (k = 2 to 14.107 Å^{-1}) in Fe EXAFS before Fourier transformation. The k-space data were then transferred into the Artemis Software for curve fitting. In order to fit the data, the Fourier peaks are isolated separately, grouped together, or the entire (unfiltered) spectrum was used. The individual Fourier peaks were isolated by applying a Hanning window to the first and last 15% of the chosen range, leaving the middle 70% untouched. Curve fitting is performed using *ab initio*-calculated phases and amplitudes from the FEFF8[7] program and *ab initio*-calculated phases and amplitudes are used in the EXAFS equation[8]

$$\chi(k) = S_0^2 \sum_j \frac{N_j}{kR_j^2} f_{eff_j}(\pi, k, R_j) e^{-2\sigma_j^2 k^2} e^{\frac{-2R_j}{\lambda_j(k)}} \sin(2kR_j + \phi_{ij}(k)) \qquad \text{(S1)}$$

where N_j is the number of atoms in the jth shell; R_j the mean distance between the absorbing atom and the atoms in the jth shell; f_{eff_j} (π,k, R_j) is the *ab initio* amplitude function for shell *j*, and the Debye-Waller term $e^{-2\sigma_j^2 k^2}$ accounts for damping due to static and thermal disorder in absorber-backscatterer distances. The mean free path term $e^{\frac{-2R_j}{\lambda_j(k)}}$ reflects losses due to inelastic scattering, where $\lambda_j(k)$, is the electron mean free path. The oscillations in the EXAFS spectrum are reflected in the sinusoidal term $\sin(2kR_j + \phi_{ij}(k))$, where $\phi_{ij}(k)$ is the *ab initio* phase function for shell *j*. This sinusoidal term shows the direct relation between the frequency of the EXAFS oscillations in k-space and the absorber-back scatterer distance. S_0^2 is an amplitude reduction factor.

The EXAFS equation (Eq. S1) is used to fit the experimental Fourier isolated data (in q-space) as well as unfiltered data (in k-space) and Fourier transformed data (in R-space) using N, S_0^2, E_0, R, and σ^2 as variable parameters**.** N refers to the number of coordination atoms surrounding Fe for each shell. The quality of fit is evaluated by R-factor and the reduced Chi^2 value. The deviation in E_0 was required to be less than or equal to 10 eV. An R-factor less than 2% denotes that the fit is good enough whereas an R-factor between 2 and 5% denotes that the fit is correct within a consistently broad model[9]. The reduced Chi^2 value is used to compare fits as more absorber-backscatter shells are included to fit the data. A smaller reduced Chi^2 value indicates a better fit. Similar results were obtained from fits done in k, q, and R-spaces.

DFT Calculations. The DFT optimization calculations were performed using the ORCA (Version 5.0) program package developed by Neese[10] and co-workers. The calculations were optimized at the BP-86 level[11-12] with the def2-TZVP[13] basis set, and the atom-pairwise dispersion correction D3BJ[14-15]. The conductor-like polarizable continuum model (CPCM)[16] was applied to model the acetonitrile solvent. The RI[17] approximation was used to accelerate Coulomb and exchange integrals. The default GRID settings were further used for the self-consistent field iterations and for the final energy evaluation. The calculated structures were confirmed to be minima based on a check of the energies and the absence of imaginary frequencies from frequency calculations carried out on the optimized geometries.

Time-dependent (TD)-DFT XANES and Optical Calculations. Time-dependent DFT (TD)-DFT calculations for the XANES and optical spectra of the Fe complexes were carried out using previously established protocols.[18-19] The TD-DFT XANES and optical simulations were in this case performed with the B3LYP as functional with the def2-TZVP triple-zeta[20] basis and D3BJ dispersion correction effects with dense integration grids. The def2-TZVP/J auxiliary basis set and conductor-like polarizable continuum model (CPCM)with acetonitrile solvent were further used. Up to 150 and 50 roots were calculated for the XAS and optical spectra, respectively. The XANES absorption spectra from the TD-DFT calculations were shifted in energy by +153 eV relative to the experimental data as previously demonstrated[21-26], and a broadening of 2.0 eV was applied to all calculated XAS spectra (FWHM) with a Gaussian line shape. The calculated XANES spectrum contains contributions from electric quadrupole, electric dipole, and magnetic dipole transitions.
By contrast, a broadening of 100 nm was applied to all calculated optical spectra (FWHM) with a Lorentzian line shape.

Table S3: Summary of parameters employed for the pre-edge fits of Complexes **1** and **3**.

**Pseudo-Voight Function**	E_0 (Centroid, eV)	σ	γ	**AREA (units)**
Complex **1**	**7113.97**	2.038	0.385	**19.3**
Complex **3**	**7114.32**	2.200	0.700	**23.5**
**Erf function**	E_0 (Centroid, eV)	w (Width)	A (Amplitude)	
Complex **1**	7139.00	16.286	0.592	

Complex **3**	7140.00	17.300	0.600

The pre-edge area peaks fitting were further re-carried out in the Fityk[27] software and as previously demonstrated[26], and the same pre-edge peak areas of 19.3 and 23.5 units were obtained for Complexes **1** and **3** oxidized with PhINTs, respectively, thus confirming the fit procedure employed in the Athena[6] software.

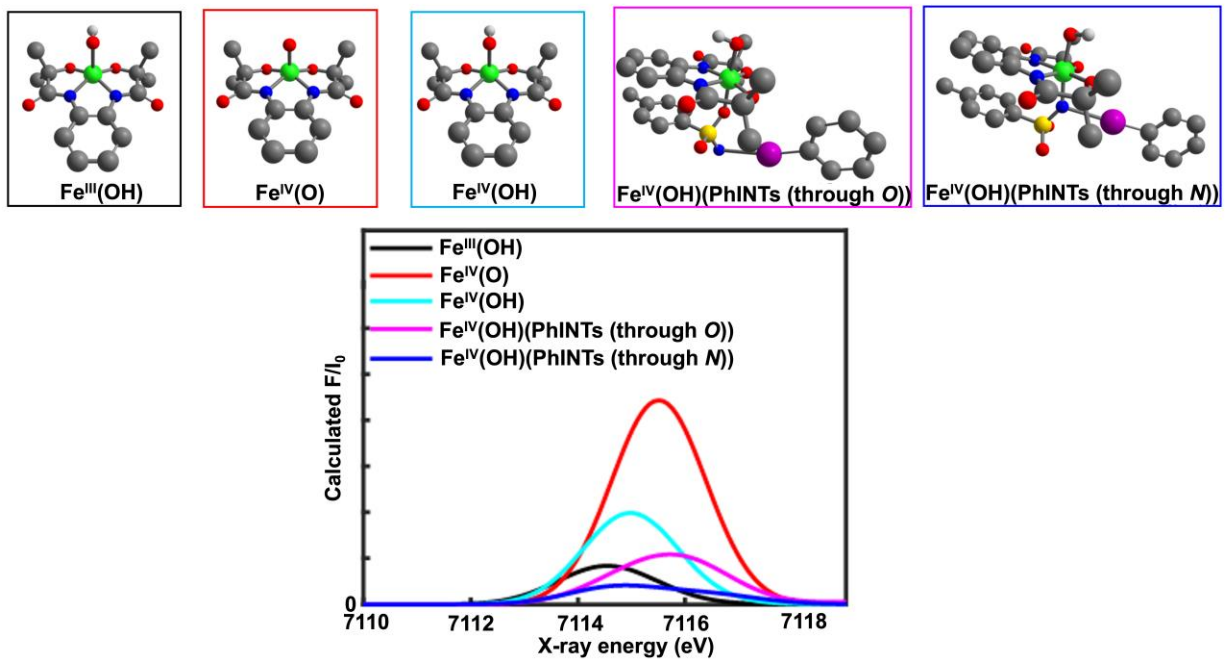

Figure S1: Calculated pre-edge areas of complex **1** (Fe^{III}(OH) complex) together with the oxidized Fe^{IV} (O), Fe^{IV}(OH) and Fe^{IV}(OH)(PhINTs) complex bound to an oxygen (complex **3**) and nitrogen atom respectively.

Summary of parameters employed for the pre-edge fits of Complexes 1 and 3.
Table S4. EXAFS Fits parameters

Complex	Fit		Shell, N	R, Å	E_0	ss.^{2} (10^{-3})	R-factor	Reduced Chi-square
**1**	1	I	Fe-N/O, 5	1.95	-2.0	7.7	0.0201	133
	2	I	Fe-O, 3<br>Fe-N, 2	1.85<br>2.00	-8.2	2.6<br>2.5	0.0057	108
	**3**	**I,II**	**Fe-O,3**	**1.88**	**-3.8**	**3.9**	**0.0098**	**46**

			**Fe-N,2**<br>**Fe-C,6**<br>**Fe-C-N/O,16**	**2.01**<br>**2.87**<br>**3.15**		**1.4**<br>**6.8**<br>**2.8**		
**3**	4	I	Fe- O/N, 5	1.86	-9.1	11	0.0099	43
	5	I	Fe- O/N, 6	1.86	-9.3	13	0.0069	30
	6	I	Fe-O, 3<br>Fe-O, 2	1.78<br>1.95	-12	3.7<br>0.2	0.0026	196
	7	I	Fe-O, 3<br>Fe-N, 2<br>Fe-O, 1	1.83<br>1.99<br>2.13	-2.6	1.4<br>4.6<br>4.6	0.0124	107
	8	I,II	Fe-O,3<br>Fe-N,2<br>Fe-C, 6<br>Fe-C-N/O, 16	1.82<br>1.97<br>2.81<br>3.14	-4.4	5.0<br>0.3<br>4.7<br>1.8	0.0241	58
	**9**	**I,II**	Fe- O,3<br>Fe- N,2<br>Fe-O, 1<br>**Fe-C,6**<br>**Fe-C-N/O,16**	1.82<br>1.97<br>2.13<br>2.87<br>3.18	-0.77	1.9<br>4.1<br>3.3<br>7.2<br>1.4	0.0099	42

* The amplitude reduction factor S_0^2 was fixed to 1. Region I refers to the EXAFS spectra region between apparent distances 1/1.9-2.3 Å, whereas Regions I and II refer to that between 1.1-3 Å. Our EXAFS data resolution determined by $\frac{\pi}{2\Delta k}$ is equal to 0.14 Å.

Table S5. Summary of DFT-calculated distances for all geometry-optimized Fe-based complexes.

**Complex**	**Fe-N_1**	**Fe-N_2**	**Fe-O_1**	**Fe-O_2**	**Fe=O**	**Fe-OH/OMe**	**Fe-OH_2**	**Fe-O (PhINTs)**	**Fe-N (PhINTs)**
**1.Fe^{III} hydroxo, Spin 1/2**	1.9216	1.9341	1.8991	1.9079		1.8985			
**2.Fe^{III} hydroxo, Spin 3/2**	1.9244	1.9252	1.9044	1.9045		1.9826			
**3.Fe^{III} hydroxo ligand oxidized**	1.9283	1.9279	1.8667	1.8675		1.8178			

**complex, S = 1**									
**4. Fe^{IV} hydroxo with oxygen bonded PhNITs ligand, S = 2**	1.8863	1.9003	1.8088	1.8099		1.8479			2.1101
**5. Fe^{IV} hydroxo with oxygen bonded PhNITs ligand, S = 1**	1.8824	1.9010	1.8131	1.8193		1.8493			2.13182
**6. Fe^{IV} hydroxo with nitrogen bonded PhNITs ligand, S = 2**	1.9050	1.8888	1.8404	1.8387		1.8763			1.9654
**7. Fe^{IV} oxo with spin 1**	1.9306	1.9298	1.9147	1.9173	1.6573				
**8. Fe^{IV} hydroxo with spin 1**	1.9283	1.9285	1.8657	1.8674		1.8175			
**9. Fe^{IV} hydroxo with a water molecule with spin 1**	1.9115	1.9110	1.8996	1.9034		1.7973	3.2253		
**10. Fe^{IV} hydroxo with an acetonitrile molecule with spin 1**	1.8863	1.8856	1.8372	1.8375		1.7983	5.9835		

**11. Fe^{III} methoxy with spin ½**	1.9365	1.9270	1.9213	1.9055		1.9221			
**12. Fe^{III} methoxy with spin 3/2**	1.9268	1.9267	1.9016	1.9028		1.9823			
**13. Fe^{III} methoxy with spin 5/2**	2.1055	2.1044	1.9353	1.9365		1.9363			
**14. Fe^{IV} methoxy with oxygen bonded PhINTs complex with spin 2**	1.8848	1.8968	1.8167	1.8132		1.8479			2.1202
**15. Fe^{IV} methoxy with nitrogen bonded PhINTs complex with spin 2**	1.8966	1.9147	1.8407	1.8469		1.8812			1.9481
**16. Fe^{III} methoxy ligand oxidized complex with spin 2**	1.9260	1.9253	1.8384	1.8376		1.9601			

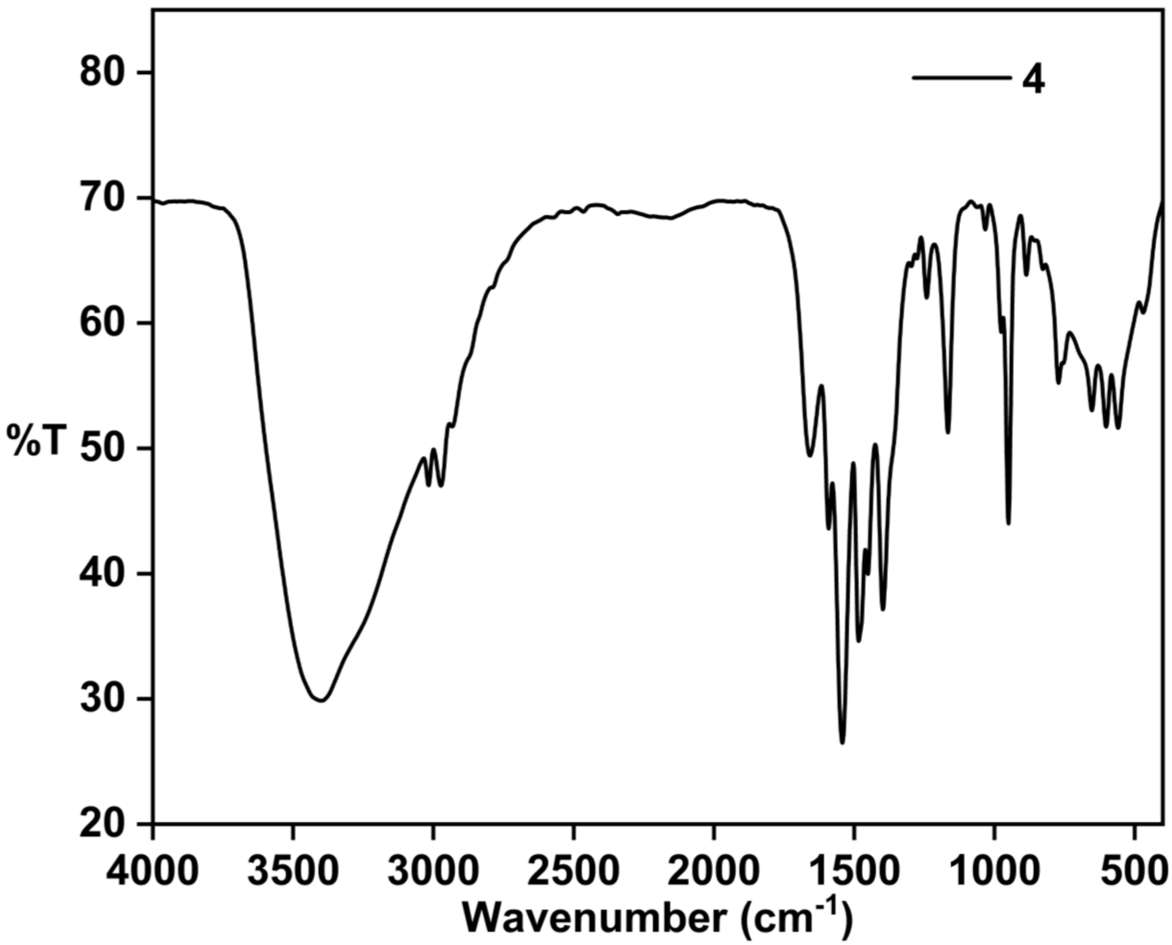

Figure S2. FT-IR spectrum of **4**, recorded on KBr pellet.

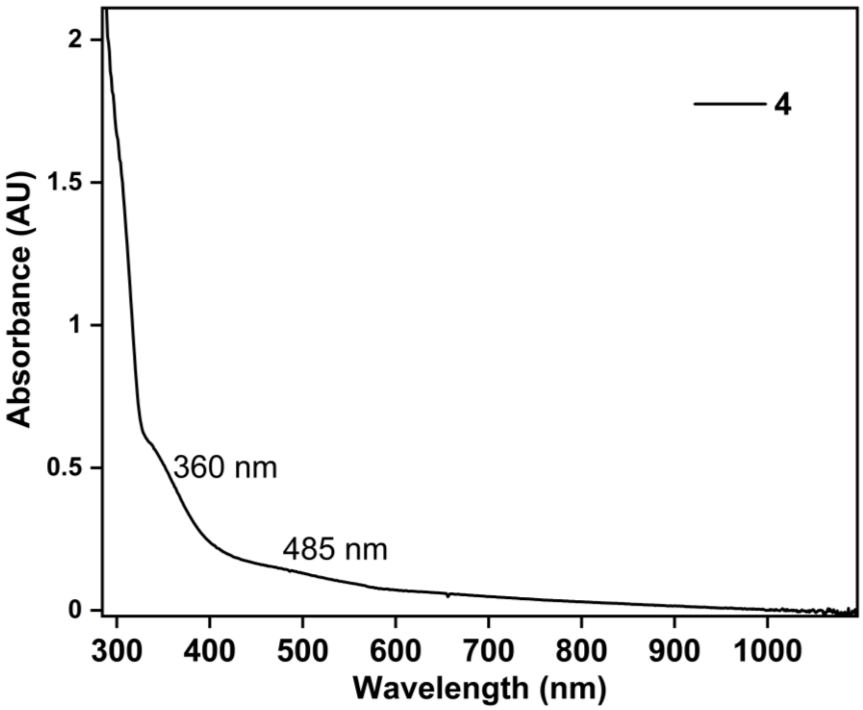

Figure S3. UV-vis spectrum of **4** (0.22 mM) in acetonitrile at 25 °C.

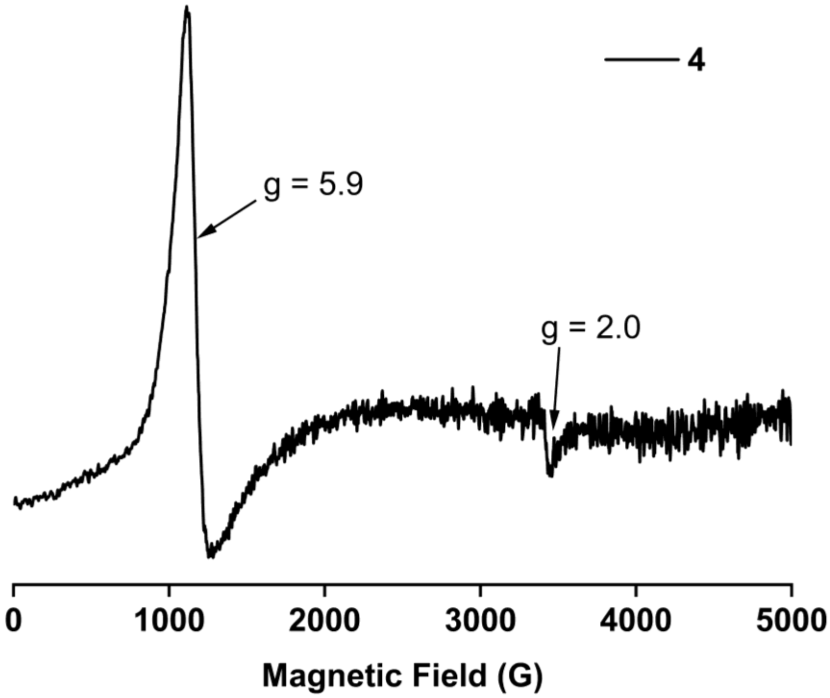

Figure S4. X-band EPR spectrum of **4** (4 mM), measured in 5:2 frozen tetrahydrofuran/methanol (v/v) at 77 K. Experimental parameters: Frequency = 9.6291 GHz, Power = 0.99 mW, Modulation frequency = 100 kHz, Modulation amplitude = 4.91 G.

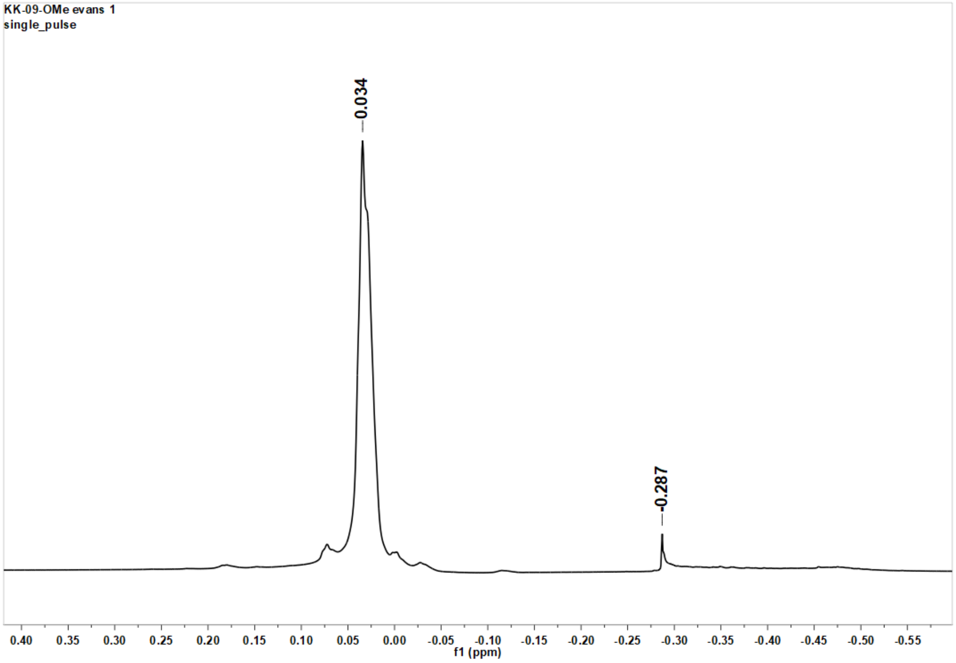

Figure S5. The shift of ^{1}H NMR signal of hexamethyldisilazane in the presence of **4** a 400 MHz NMR instrument for the estimation of the solution magnetic moment of **4** (5.55 mM) in CD_3OD at 25 °C.

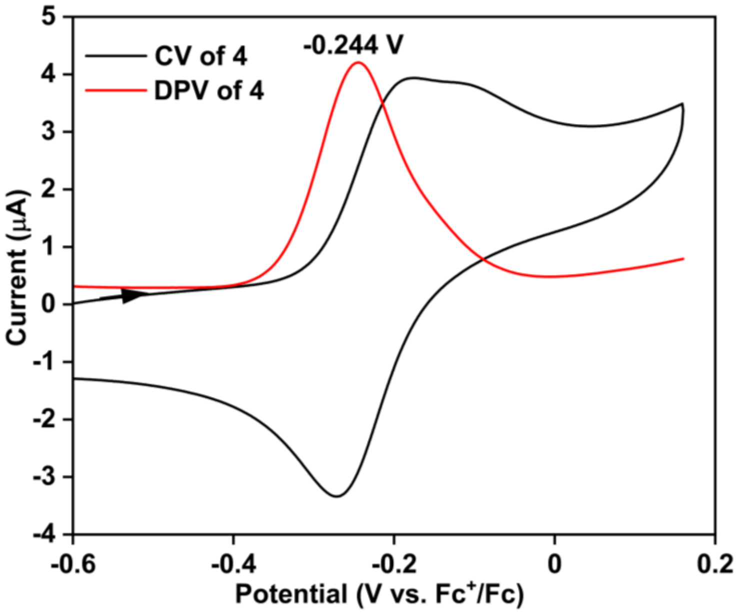

Figure S6. Cyclic voltammogram and differential pulse voltammogram of **4** (0.5 mM) in acetonitrile in the presence of $^nBu_4NPF_6$ (50 mM) as the supporting electrolyte. A 3 mm glassy carbon as the working electrode, a Pt wire as the counter electrode, and an Ag wire as a pseudo reference electrode were used during the CV measurement. The potential value is reported with respect to the Fc^+/Fc couple. CV data was recorded at a scan rate of 100 mV/s.

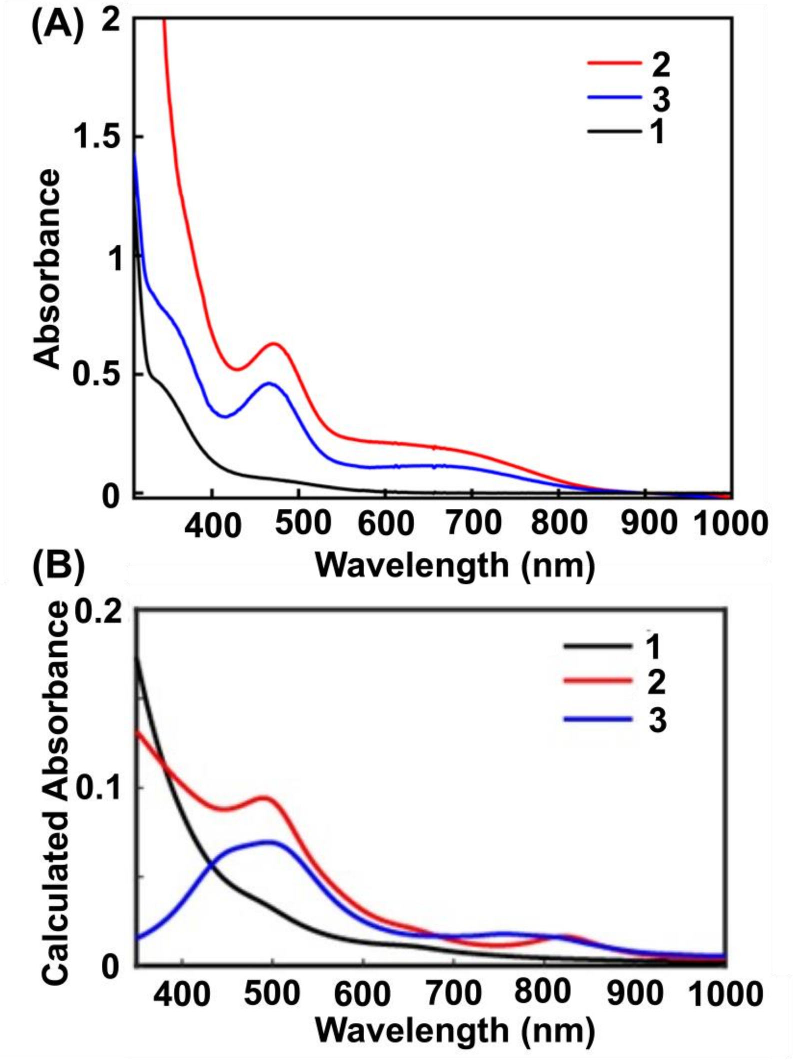

Figure S7 A. UV-vis spectra of complex **1** (0.15 mM) and intermediates formed upon the addition of PhINTs (3 equiv, **3**) and magic blue (one equiv, **2**) to the solution of **1** (0.25 mM) in acetonitrile at –25 °C. **B**. Calculated TD-DFT optical data of complexes **1**, **2** and **3**. While complex **1** shows a featureless optical spectrum, **2** and **3** demonstrate 2 peaks in the visible light region in agreement with experimental data. The calculated optical spectra of **2** and **3** are further very similar.

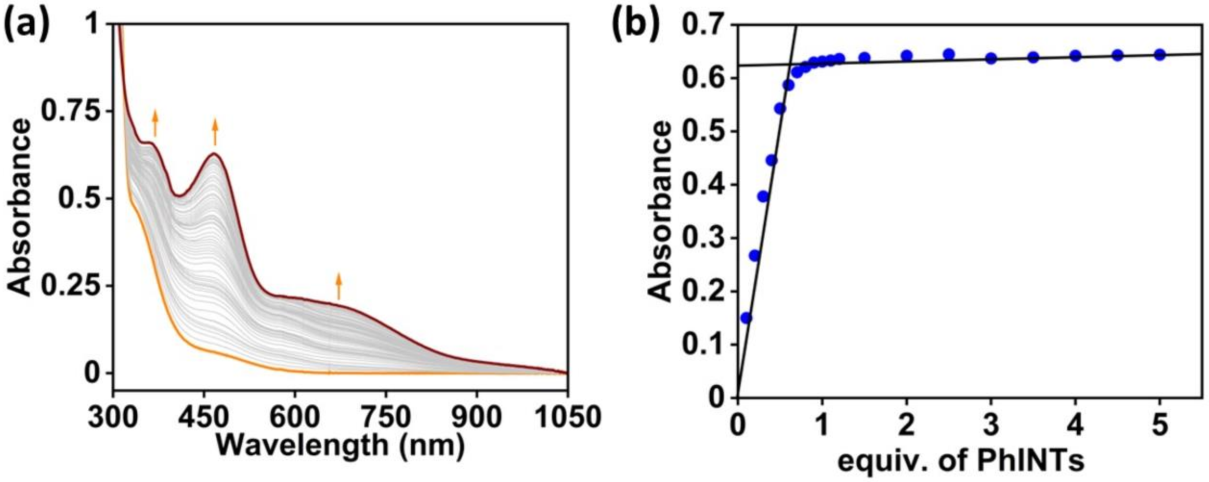

Figure S8. (A) The UV-vis spectrum of the reaction solution obtained upon adding different equiv. of PhINTs to an acetonitrile solution of **1** at –45 °C. (B) A plot of absorbance vs. equiv. of PhINTs added.

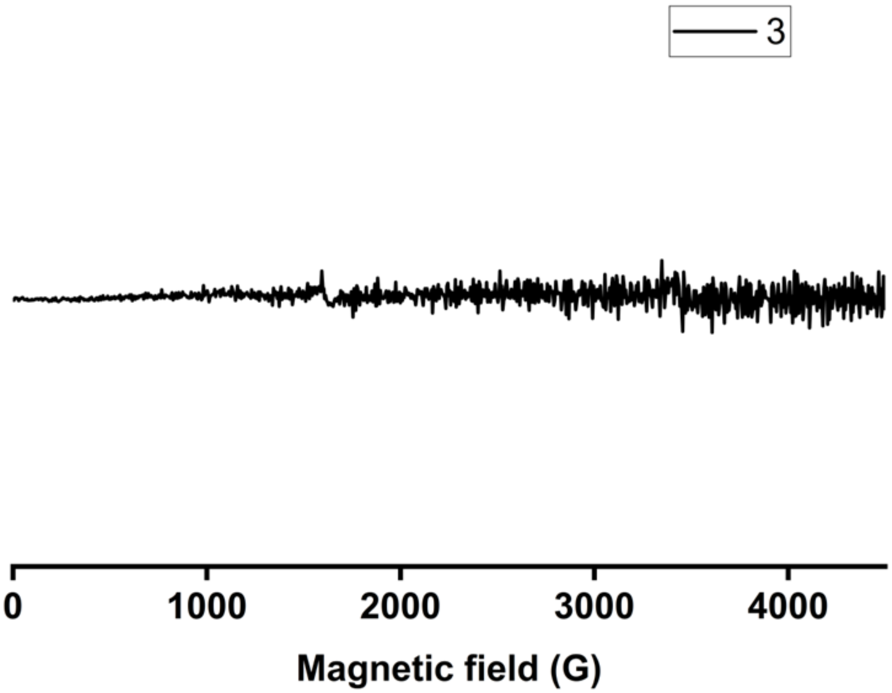

Figure S9. X-band EPR spectrum of **3** (2 mM) in frozen acetonitrile at 77 K.

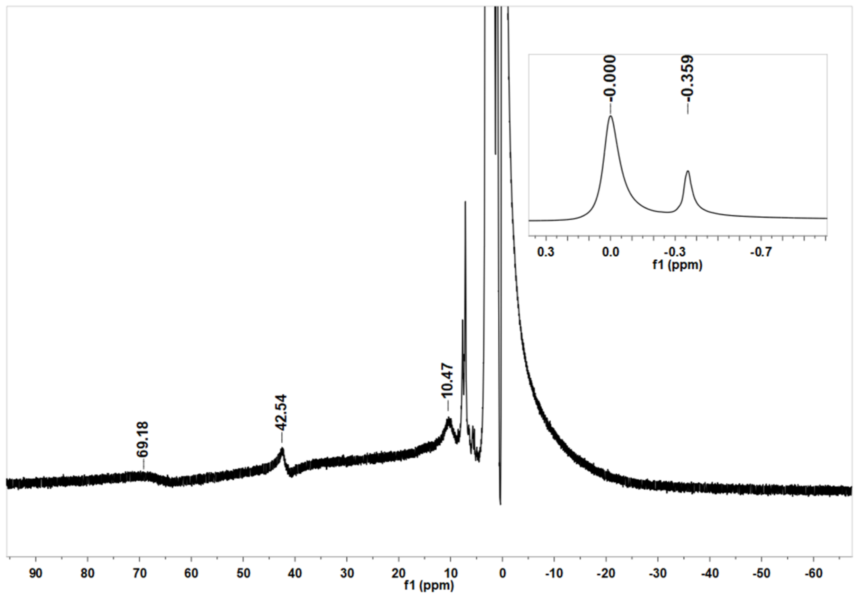

Figure S10. ^{1}H-NMR spectrum of complex **3** in CD_3CN at –10 °C in a 500 MHz NMR instrument. Inset: shift of ^{1}H NMR signal of hexamethyldisilazane in a 500 MHz NMR instrument for the estimation of the magnetic moment of **3** (8.48 mM) in CD_3CN at –10 °C.

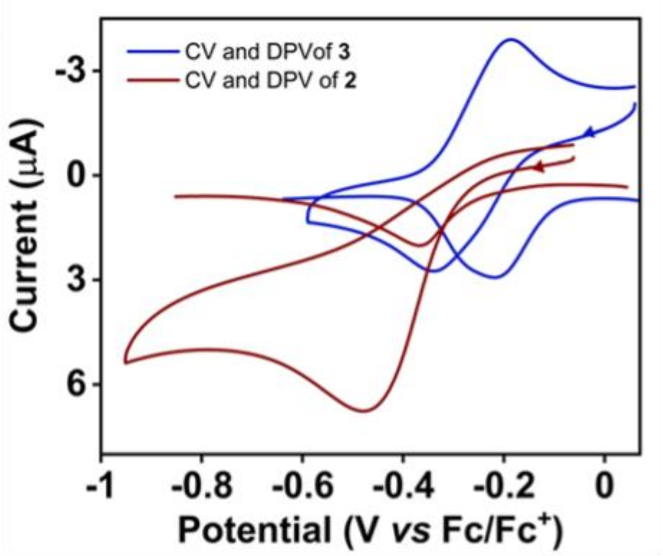

Figure S11. CV and DPV data of **3** (0.5 mM) and **2** (0.5 mM) in acetonitrile containing 50 mM of tetrabutylammonium hexafluorophosphate as supporting electrolyte at –15 °C.

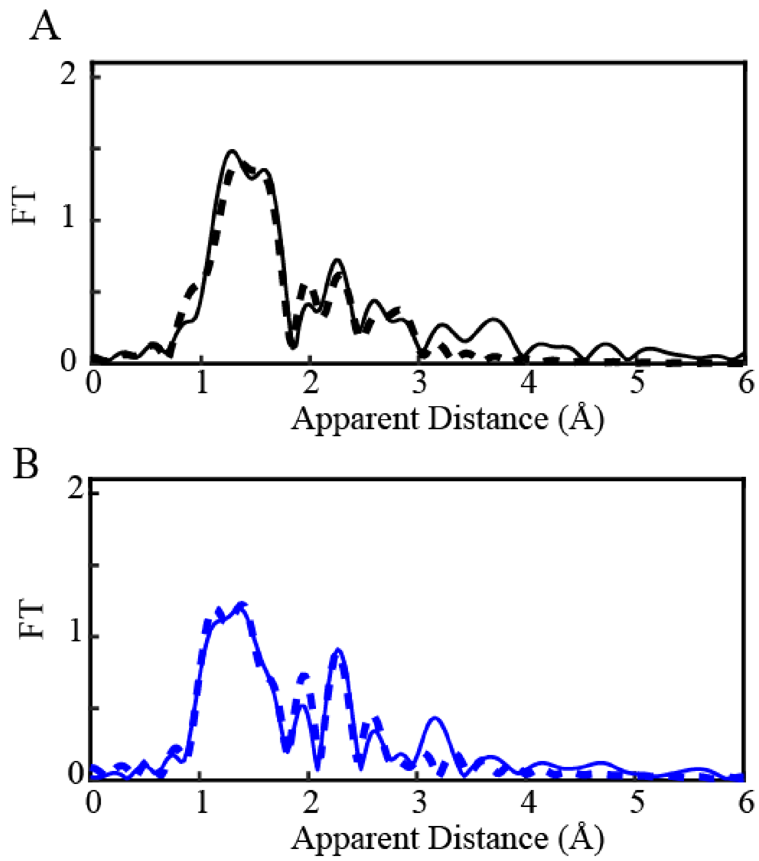

Figure S12. Fourier transforms of k^2-weighted Fe EXAFS for **A**. **1** (solid black line) and its corresponding fit (Fit 3, Table S4), for **B. 3** generated with PhINTs, and its corresponding fit (Fit 9, Table S4).

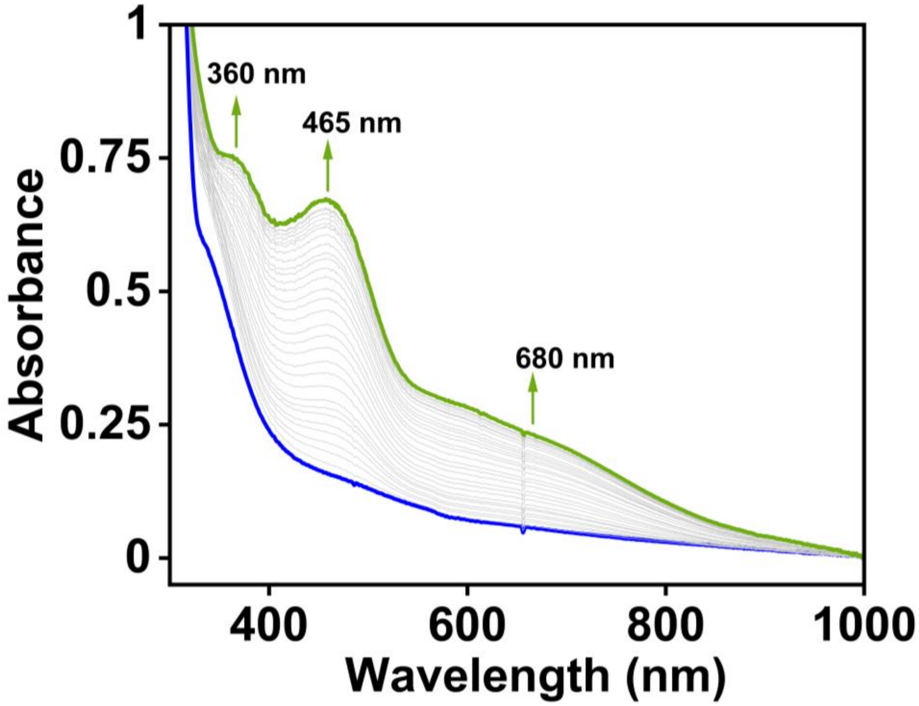

Figure S13. Change of UV-vis spectrum of **4** (0.22 mM) upon addition of PhINTs (3 equiv.) in acetonitrile at –45 °C.

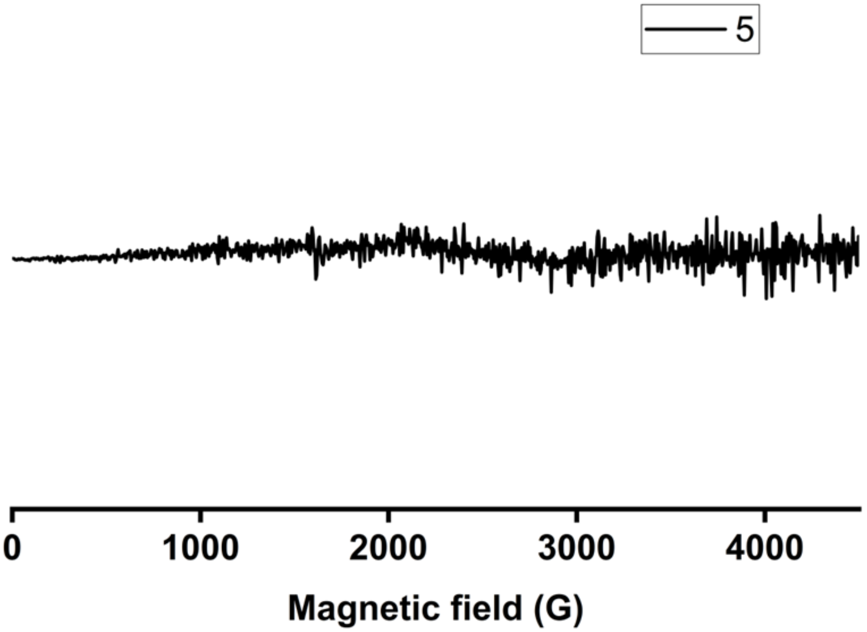

Figure S14. X-band EPR spectrum of **5** (4 mM) measured in frozen acetonitrile at 77 K.

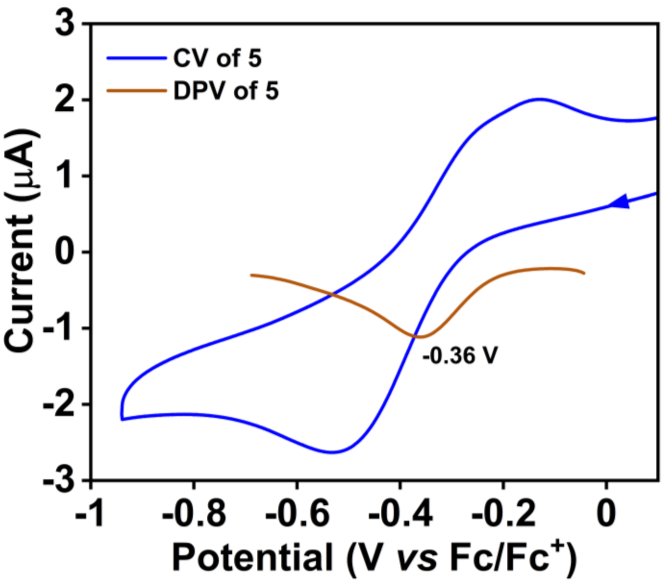

Figure S15. Cyclic voltammogram and differential pulse voltammogram of **5** (0.5 mM) measured in acetonitrile at around −15 °C.

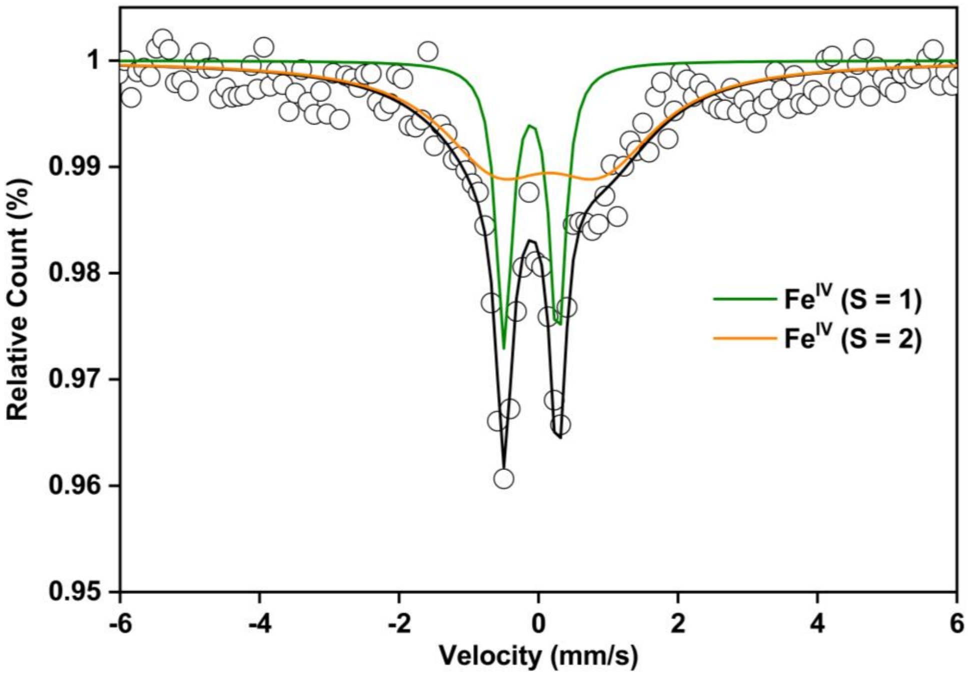

Figure S16. Zero-field ^{57}Fe Mossbauer spectrum of the reaction solution obtained upon adding PhINTs (3 equiv) to an acetonitrile solution of **4** at −25 °C. The data was recorded at a liquid nitrogen temperature. The Fe^{IV} species (S = 1, ~40 %) revealed δ = –0.11 mm/s and ΔE_q = 0.77 mm/s. While the Fe^{IV} species (S=2, ~60 %) showed δ = 0.17 mm/s and ΔE_q = 1.43 mm/s.

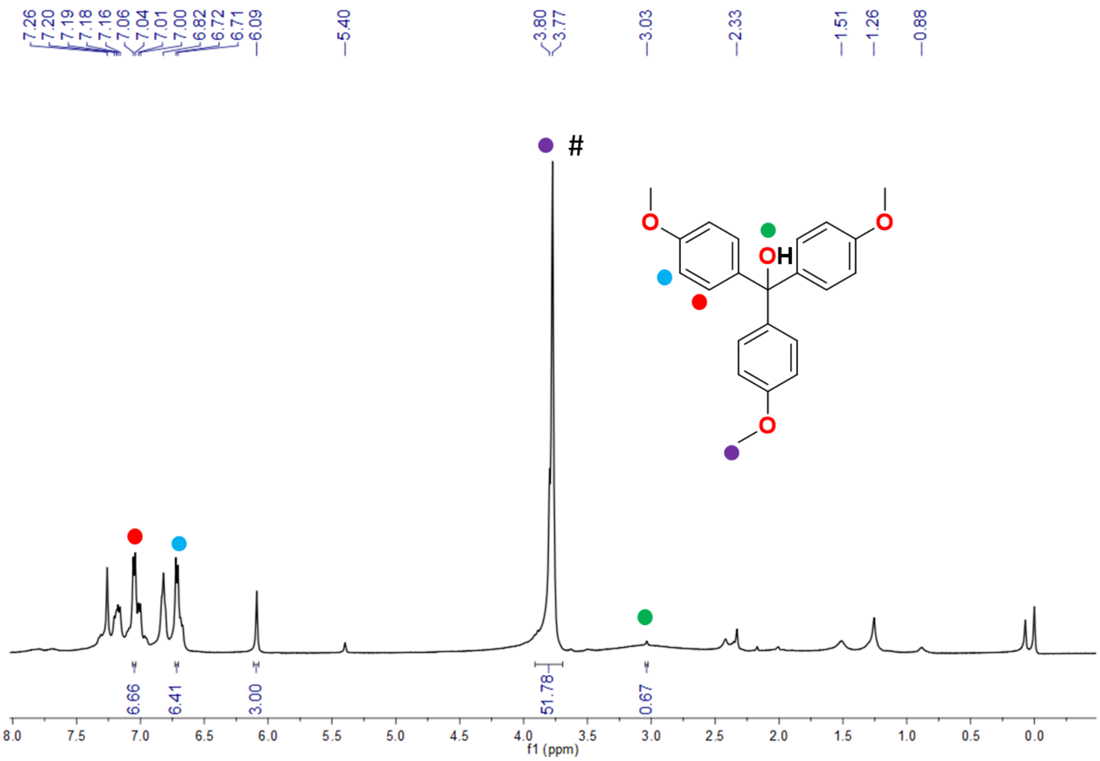

Figure S17. ^{1}H NMR spectrum ($CDCl_3$, 500 MHz, 25 °C) of the reaction mixture obtained after reaction of **3** with (*p*-OMe-C_6H_4)$_3$C$^\bullet$ ($^\#$Trimethoxy benzene as internal standard) in 1:4 acetonitrile/toluene (*v*/*v*).

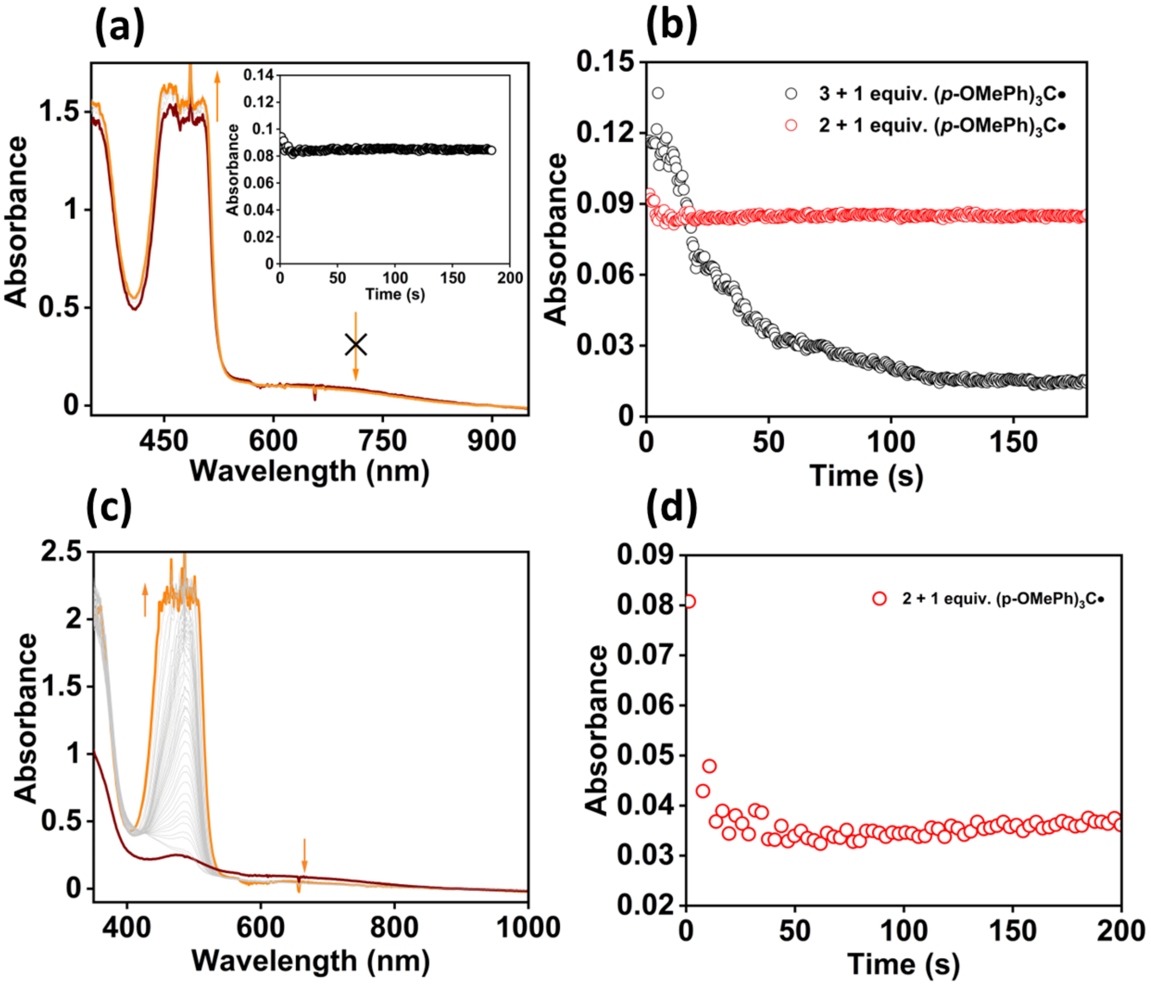

Figure S18. (a) Change of UV-vis spectrum of **2** upon addition of one equiv. of (4-OMe-$C_6H_4)_3C^{\bullet}$ in a 1:4 acetonitrile/toluene (v/v) solution at –60 °C (Inset: Time trace of **2** (0.2 mM) at 680 nm upon addition of 0.2 mM of (4-OMe-$C_6H_4)_3C^{\bullet}$), at –60 °C. (b) Comparison of decay of **2** and **3** (0.2 mM) at 680 nm in the presence of 0.2 mM of (4-OMe-$C_6H_4)_3C^{\bullet}$) at –60 °C. (c) Change of UV-vis spectrum of **2** (0.2 mM) upon addition of one equiv. of (4-OMe-$C_6H_4)_3C^{\bullet}$ to 1:4 acetonitrile/toluene (v/v) solution of **2** at –10 °C (d) Time trace of **2** (0.2 mM) at 680 nm upon addition of 0.2 mM of (4-OMe-$C_6H_4)_3C^{\bullet}$) at –10 °C.

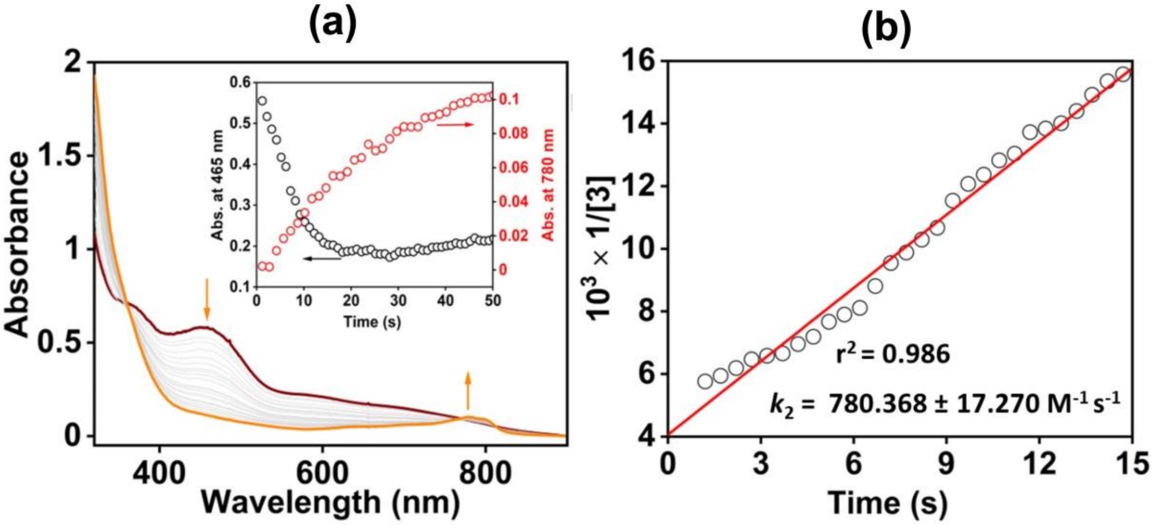

Figure S19. (a) Change of UV-vis spectrum of **3** (0.21 mM) upon addition of one equiv. of decamethylferrocene (Fc^*) to an acetonitrile solution of **3** at –25 °C (Inset: Decay of **3** (0.21 mM) at 465 nm and formation of Fc^{*+} at 780 nm upon addition of 0.21 mM of Fc^*). (b) A plot of 1/[**3**] vs. time (s) for the determination of k_2 value for one-electron reduction of **3**.

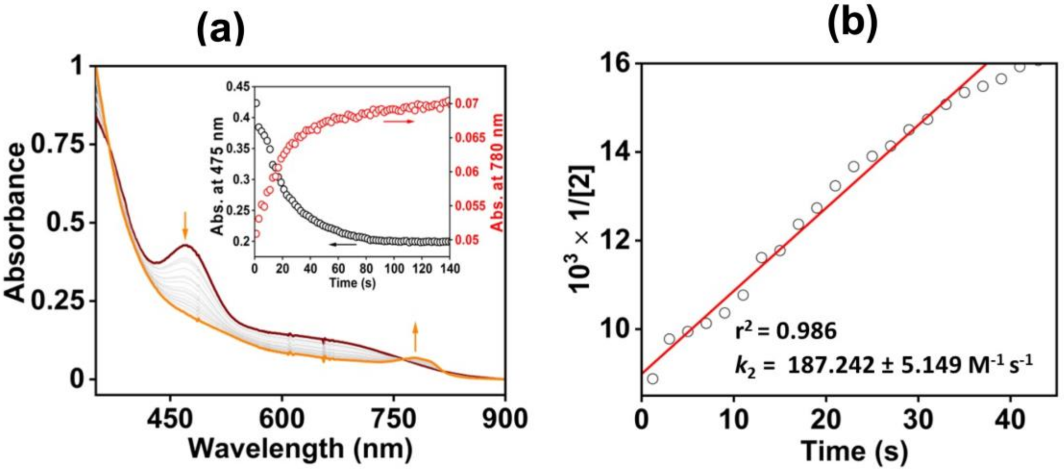

Figure S20. (a) Change of UV-vis spectrum of **2** (0.12 mM) upon addition of one equiv. of decamethylferrocene (Fc^*) to an acetonitrile solution of **2** at –20 °C (Inset: Decay of **2** (0.12 mM) at 465 nm and formation of (Fc^{*+}) at 780 nm upon addition of 0.12 mM of Fc^*). (b) A plot of 1/[**2**] vs. time for the determination of k_2 value for one-electron reduction of **2**.

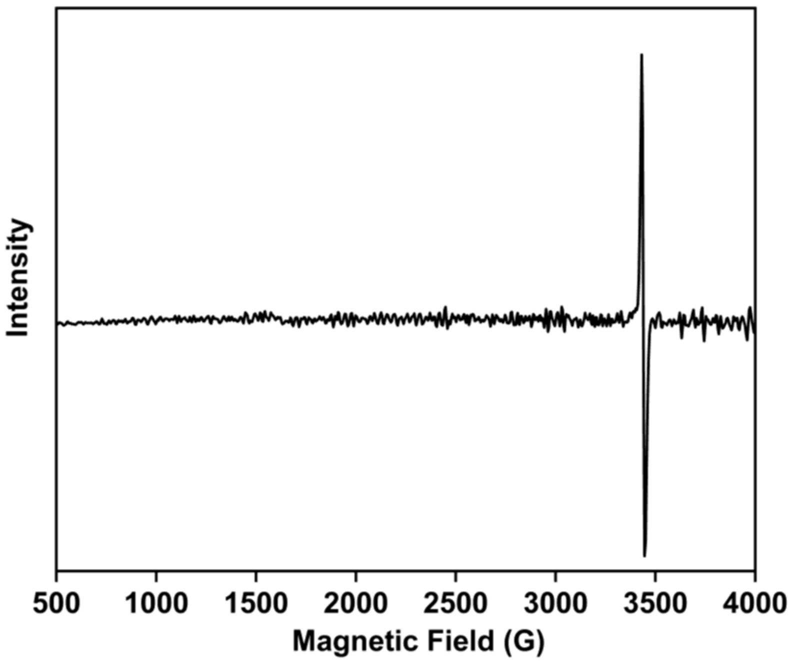

Figure S21. The X-band EPR spectrum of the reaction solution was obtained upon adding one equiv. of 4-OMe-DTBP to an acetonitrile solution of **3** (2 mM) at −20 °C.

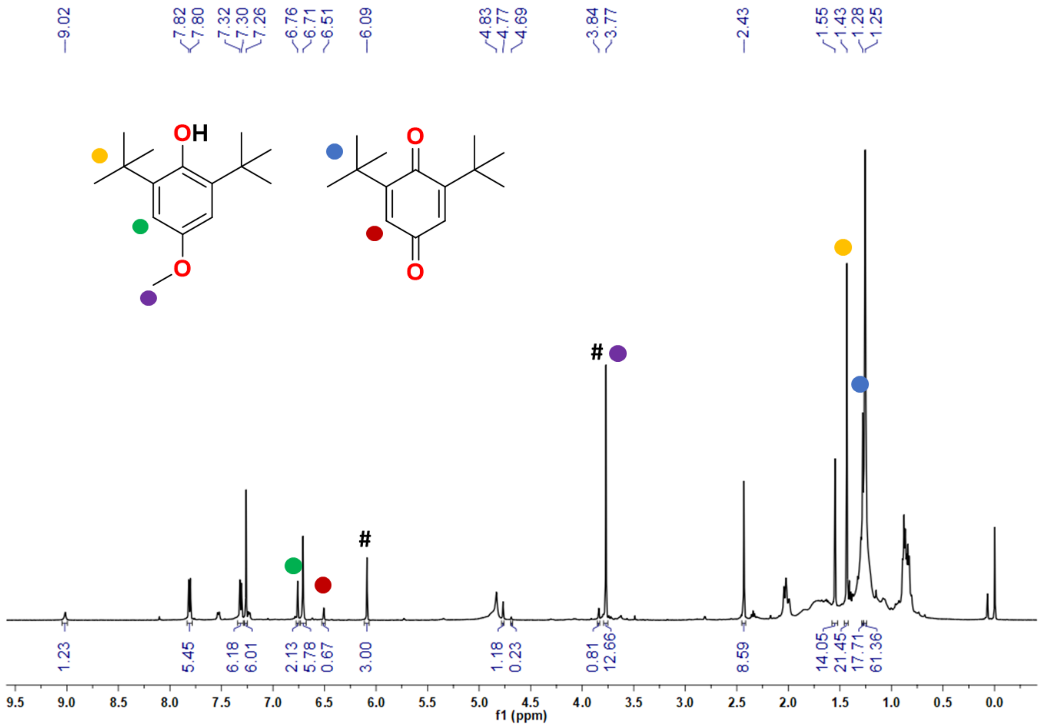

Figure S22. ^{1}H NMR spectrum ($CDCl_3$, 500 MHz, 25 °C) of the reaction mixture obtained after reaction of **3** with 4-OMe-DTBP followed by acid workup ($^{\#}$Trimethoxy benzene).

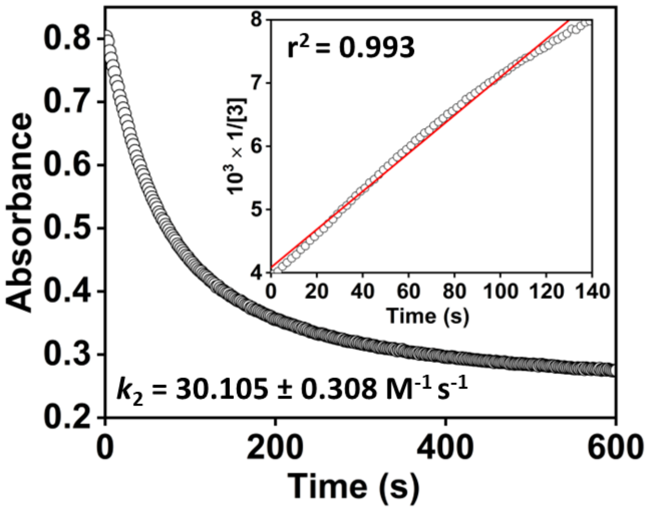

Figure S23. Decay of **3** (0.25 mM) at 465 nm upon addition of 4-OMe-DTBP at −45 °C. (Inset: Second-order fitting of the time trace at 465 nm at −45 °C of the reaction of **3** (0.25 mM) with 4-OMe-DTBP (0.25 mM) in acetonitrile)

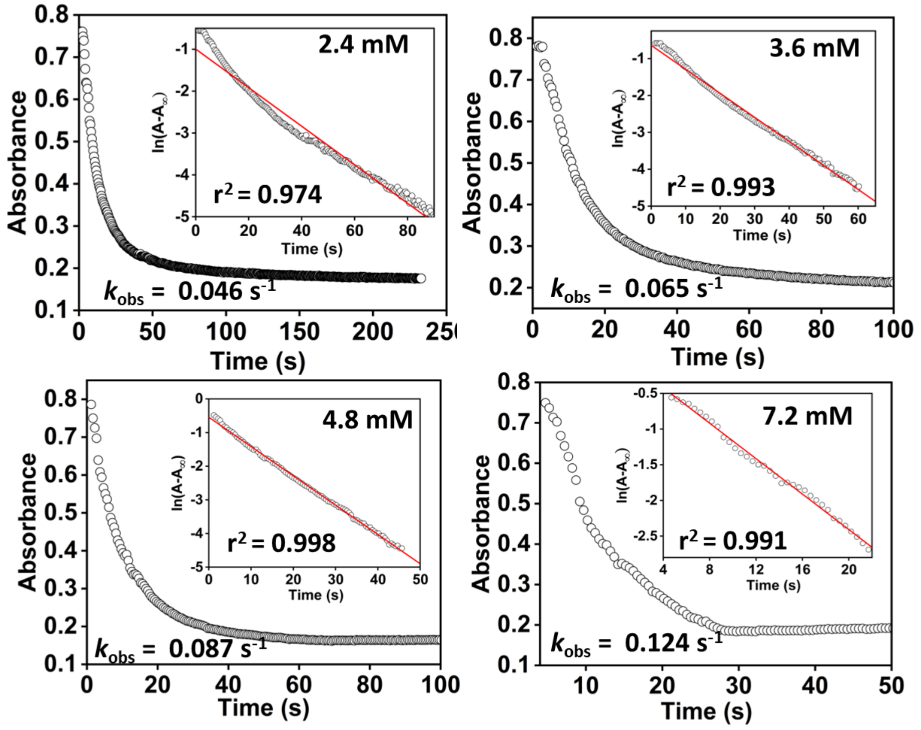

Figure S24. Decay of **3** (0.25 mM) at 465 nm upon addition of different concentrations (2.4 – 7.2 mM) of 4-OMe-DTBP-*d* (Inset: determination of k_{obs} value). The reaction was studied at −45 °C.

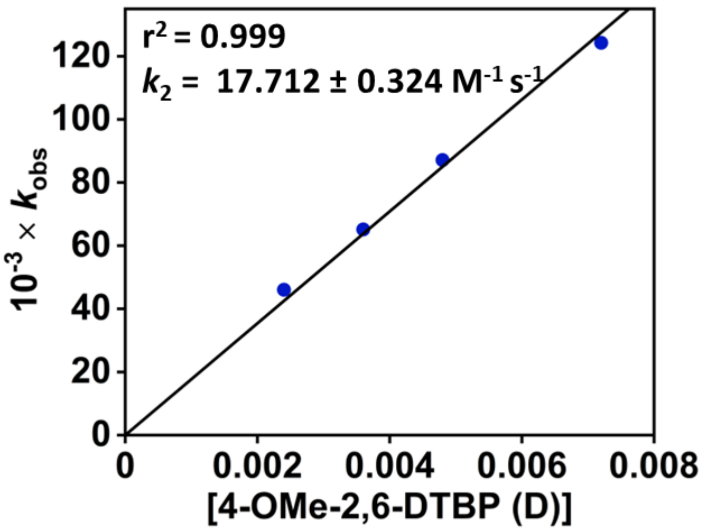

Figure S25. A plot of k_{obs} vs. [4-OMe-DTBP-*d*]. The k_{obs} values were obtained by reacting **3** with different concentrations of 4-OMe-2,6-DTBP-*d* at –45 °C in acetonitrile. The second-order rate constant was obtained from the slope of the plot.

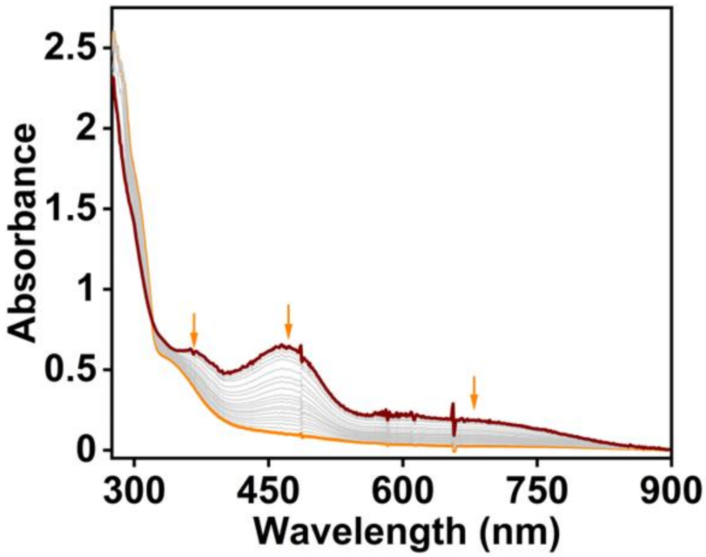

Figure S26. Change of single spectrum of **3** (0.22 mM) upon addition of 2.4 mM solution of 4-Me-DTBP in acetonitrile at –45 °C.

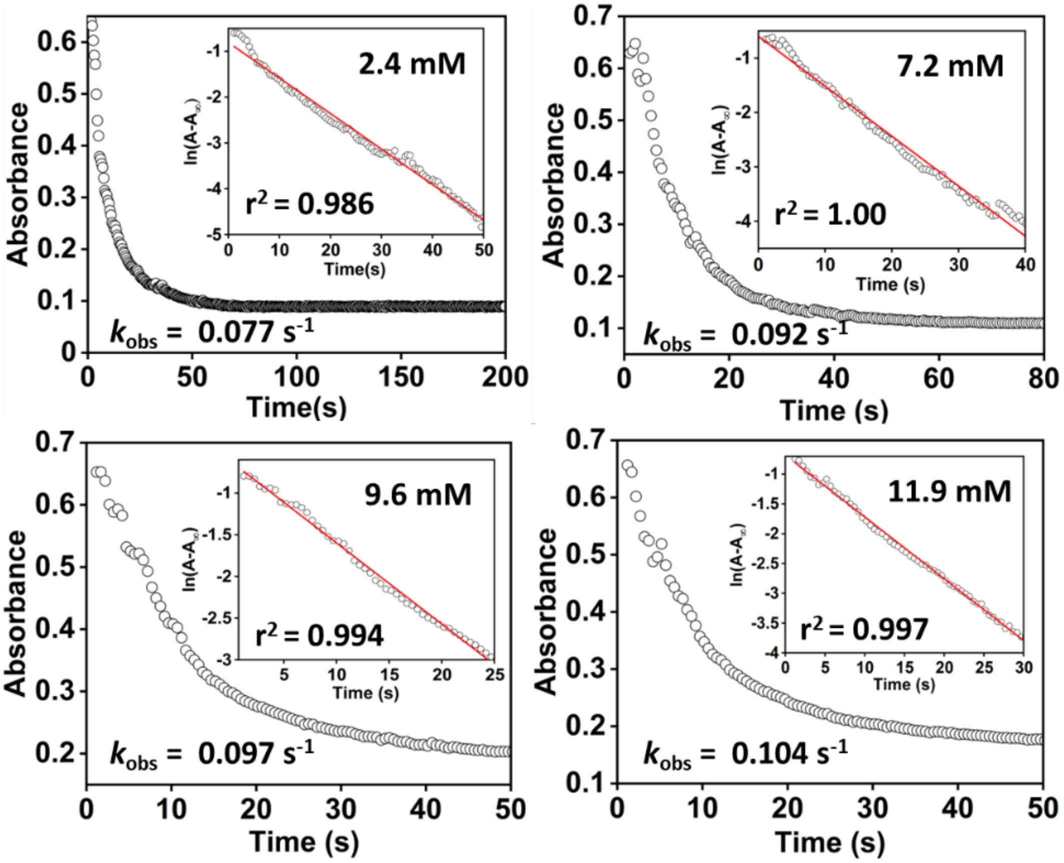

Figure S27. Decay of **3** (0.22 mM) at 465 nm upon addition of different concentrations (2.4–7.2 mM) of 4-Me-DTBP (Inset: determination of k_{obs} value). The reaction was studied at –45 °C.

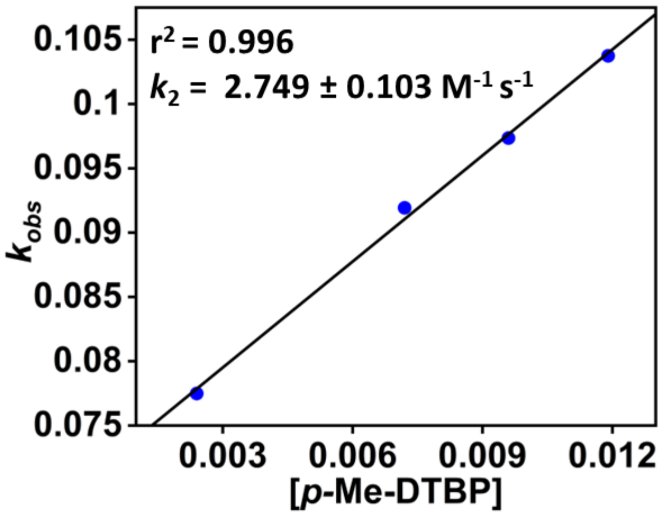

Figure S28. A plot of k_{obs} vs. [4-Me-2,6-DTBP]. The k_{obs} values were obtained by reacting **3** with different concentrations of 4-Me-DTBP at –45 °C in acetonitrile. The second-order rate constant was obtained from the slope of the plot.

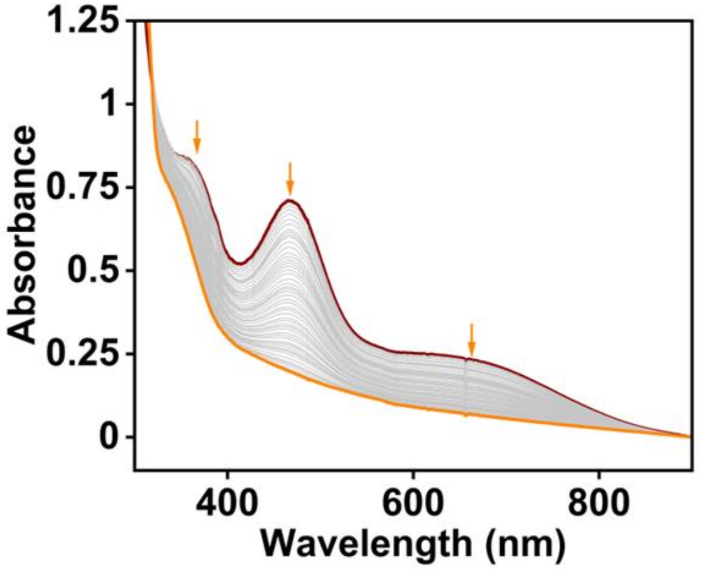

Figure S29. Change of single spectrum of **3** (0.25 mM) upon addition of 2.4 mM solution of 4-Et-DTBP in acetonitrile at –45 °C.

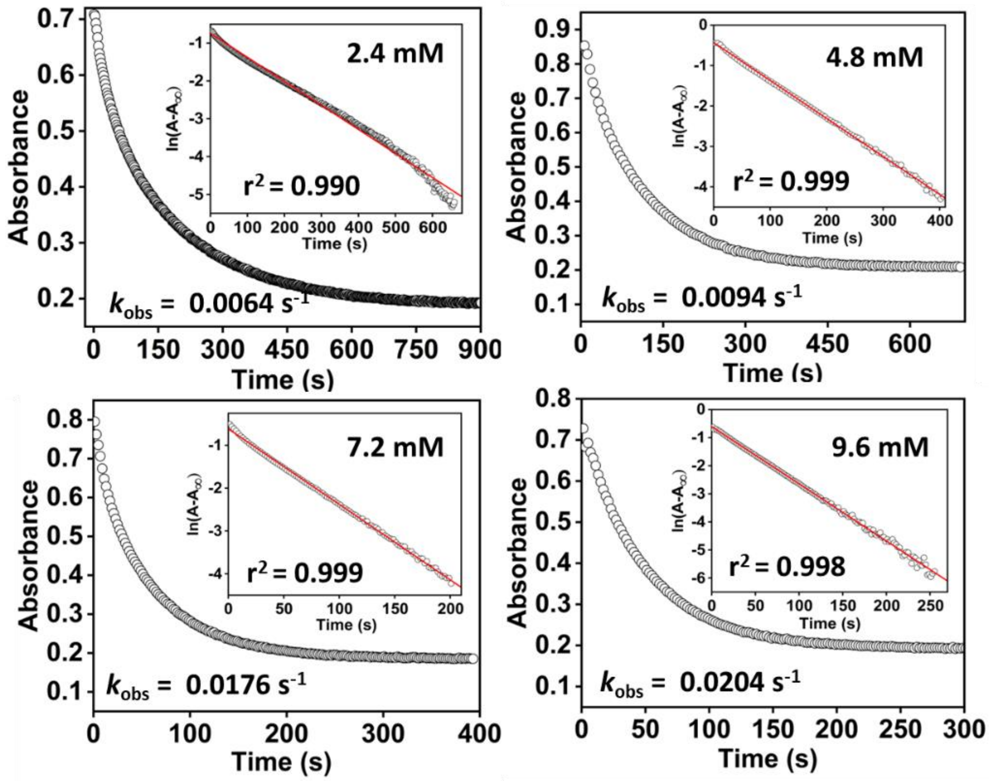

Figure S30. Decay of **3** (0.25 mM) at 465 nm upon addition of different concentrations (2.4 – 9.6 mM) of 4-Et-DTBP (Inset: determination of k_{obs} value). The reaction was studied at –45 °C.

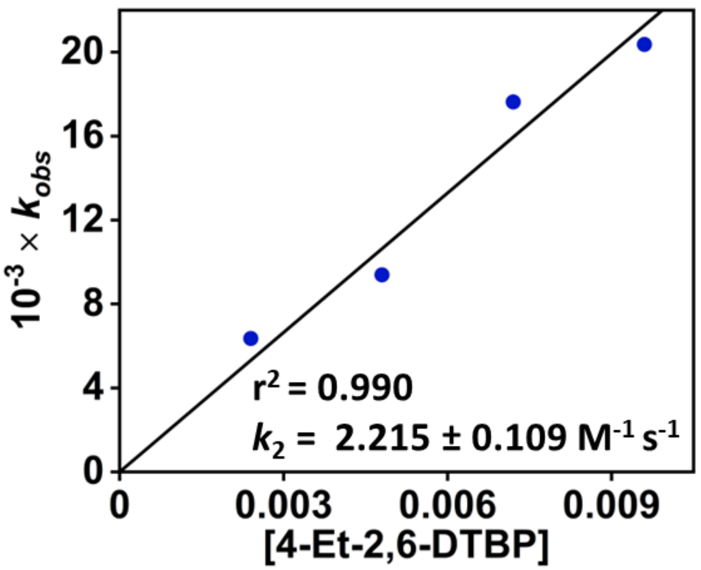

Figure S31. A plot of k_{obs} vs. [4-Et-DTBP]. The k_{obs} values were obtained by reacting **3** with different concentrations of 4-Et-DTBP at –45 °C in acetonitrile. The second-order rate constant was obtained from the slope of the plot.

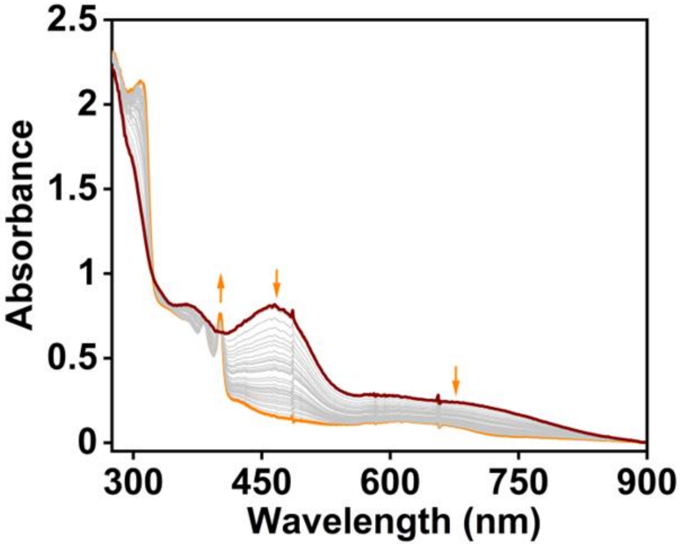

Figure S32. Change of single spectrum of **3** (0.25 mM) upon addition of 4.8 mM solution of 4-^{t}Bu-DTBP in acetonitrile at –45 °C.

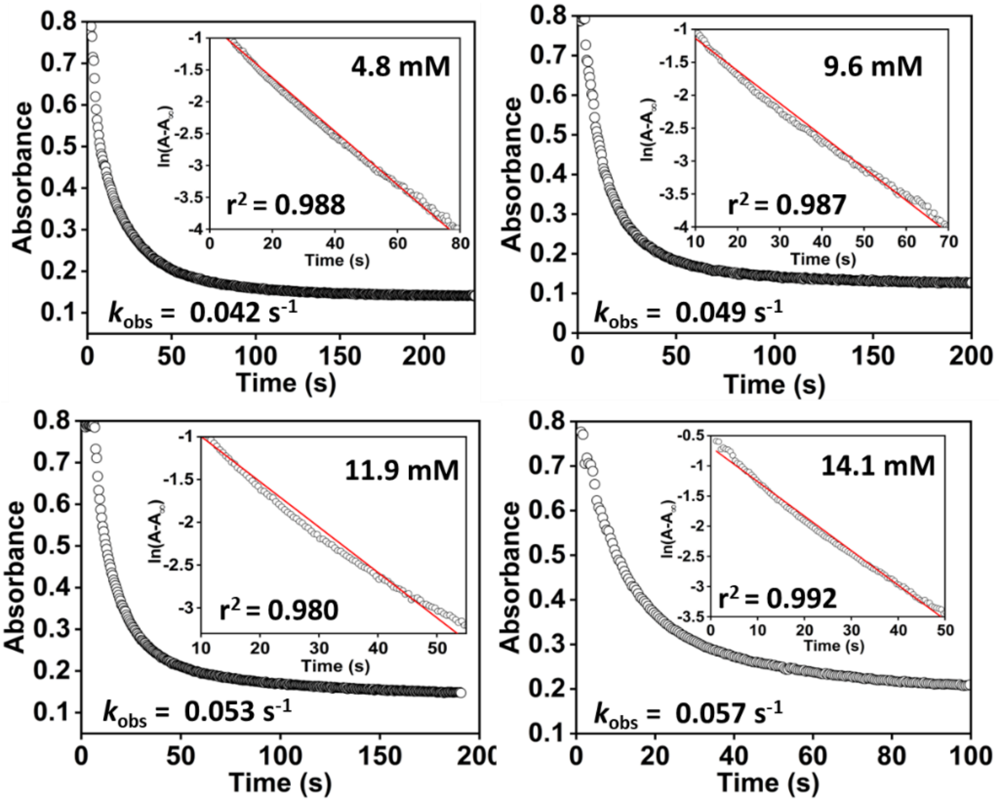

Figure S33. Decay of **3** (0.25 mM) at 465 nm upon addition of different concentrations (4.8 – 14.1 mM) of 4-^{t}Bu-DTBP (Inset: determination of k_{obs} value). The reaction was studied at –45 °C.

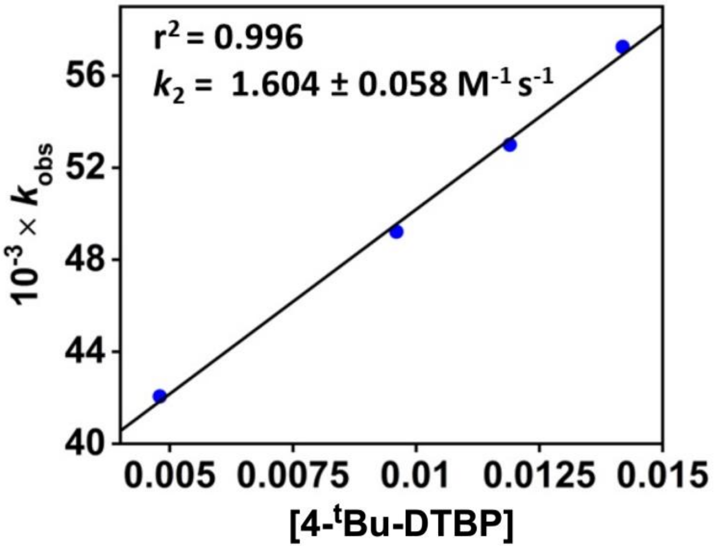

Figure S34. A plot of k_{obs} vs. [4-^{t}Bu-DTBP]. The k_{obs} values were obtained by reacting **3** with different concentrations of 4-^{t}Bu-DTBP at –45 °C in acetonitrile. The second-order rate constant was obtained from the slope of the plot.

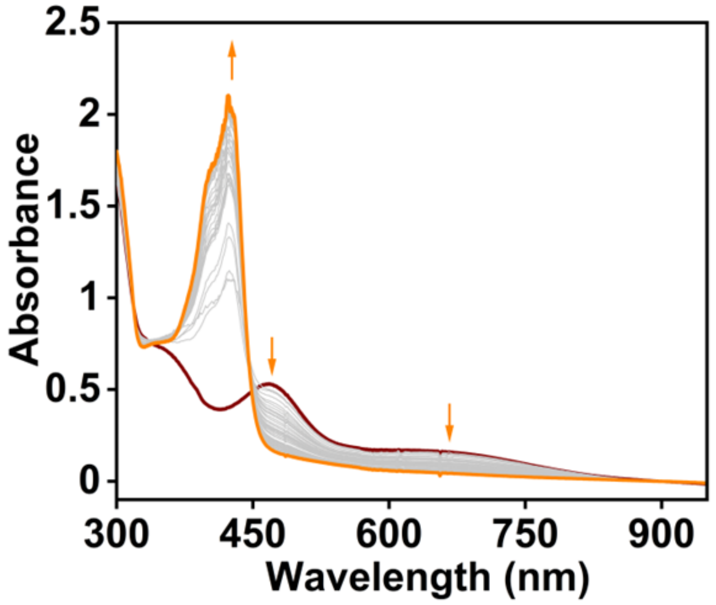

Figure S35. Change of single spectrum of **3** (0.2 mM) upon addition of 2.4 mM solution of 4-H-DTBP in acetonitrile at –45 °C.

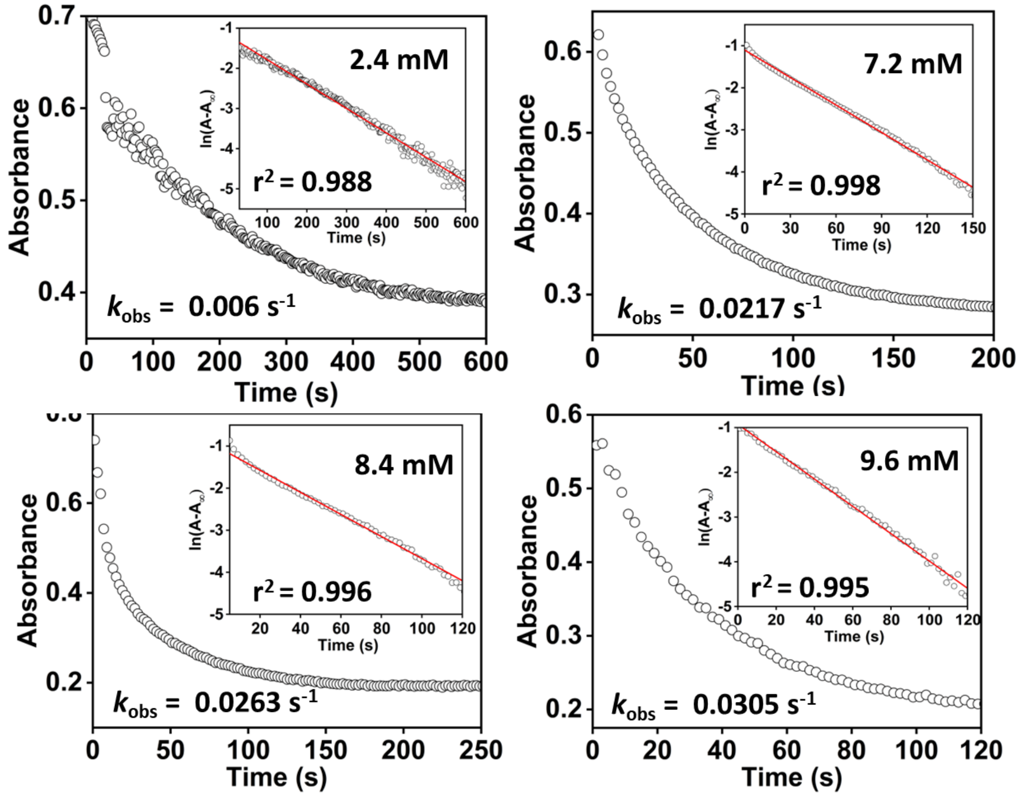

Figure S36. Decay of **3** (0.2 mM) at 465 nm upon addition of different concentrations (2.4 – 9.6 mM) of 4-H-DTBP (Inset: determination of k_{obs} value). The reaction was studied at –45 °C.

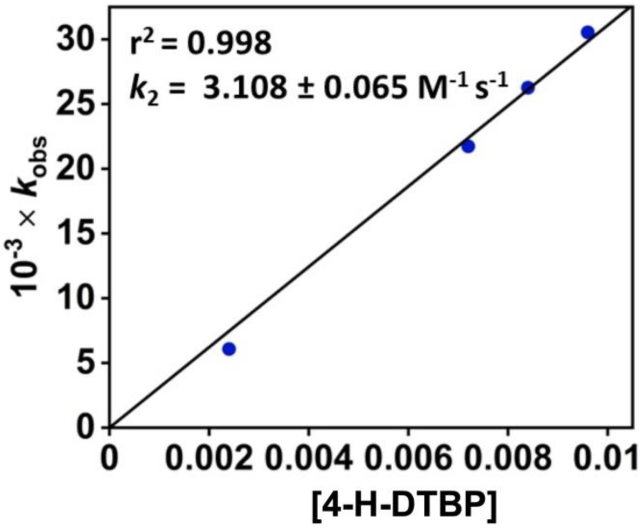

Figure S37. A plot of k_{obs} vs. [4-H-DTBP]. The k_{obs} values were obtained by reacting **3** with different concentrations of 4-H-DTBP at –45 °C in acetonitrile. The second-order rate constant was obtained from the slope of the plot.

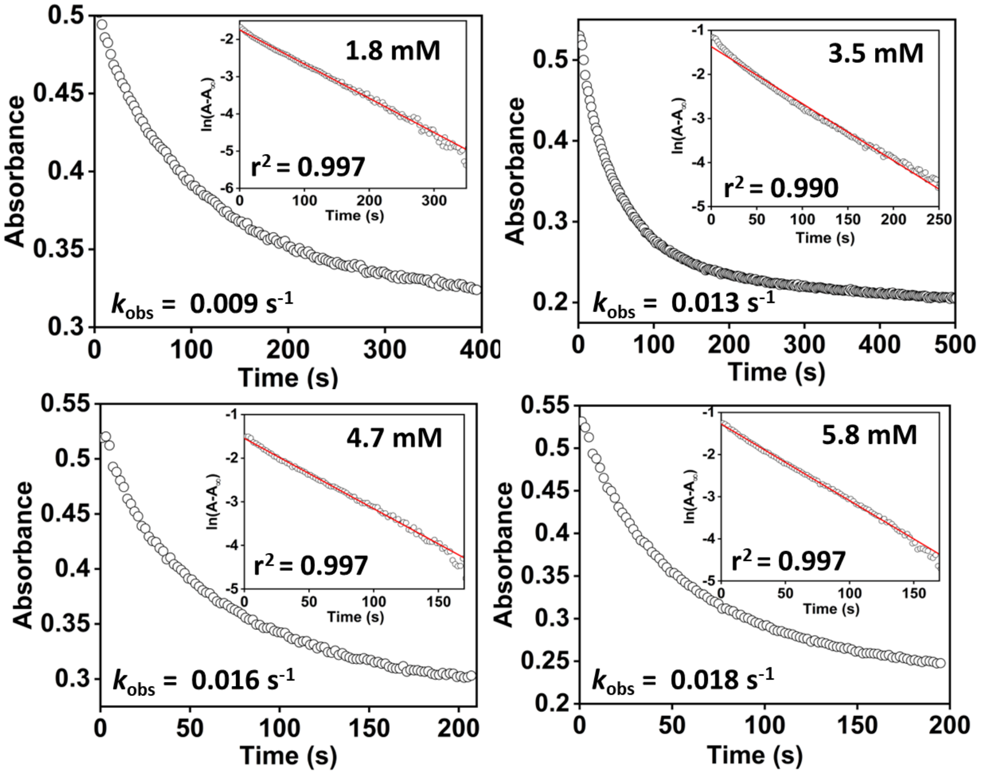

Figure S38. Decay of **3** (0.17 mM) at 465 nm upon addition of different concentrations (1.8–5.8 mM) of 2,6-DTBP-*d* (Inset: determination of k_{obs} value). The reaction was studied at –45 °C.

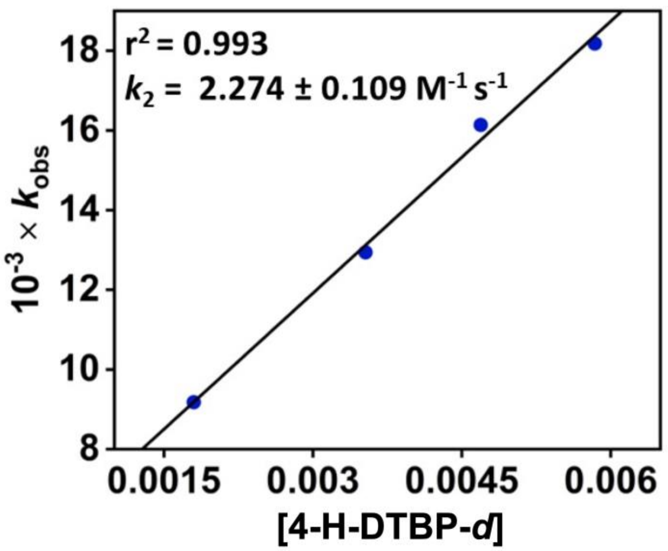

Figure S39. A plot of k_{obs} vs. [4-H-DTBP-*d*]. The k_{obs} values were obtained by reacting **3** with different concentrations of 4-H-DTBP-*d* at –45 °C in acetonitrile. The second-order rate constant was obtained from the slope of the plot.

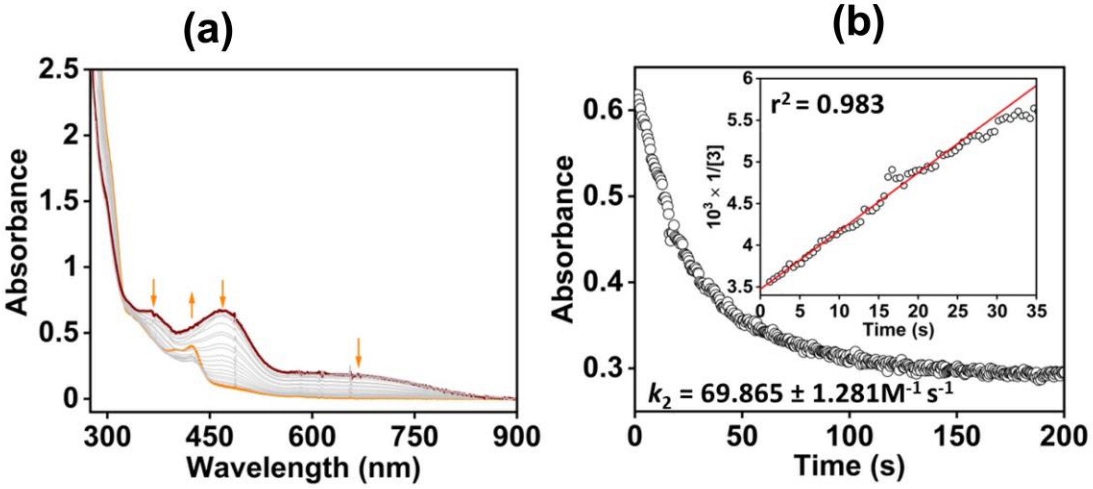

Figure S40. (a) Change of single spectrum of **3** (0.19 mM) upon addition of 0.19 mM solution of 4-Br-DTBP in acetonitrile at –45 °C. (b) Decay of **3** (0.19 mM) at 465 nm upon addition of 0.19 mM of 4-Br-DTBP (Inset: Second-order fitting of the time trace at 465 nm at –45 °C).

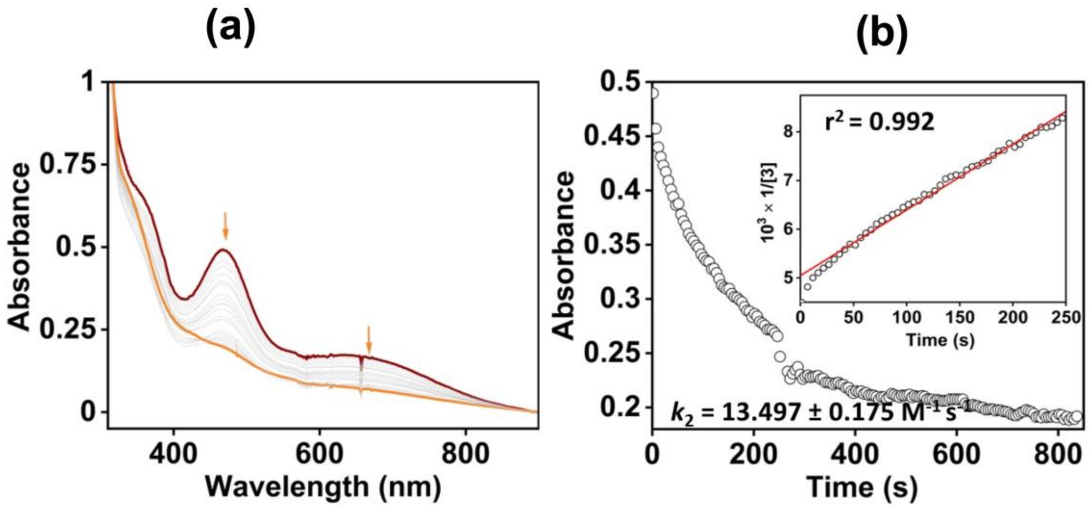

Figure S41. (a) Change of single spectrum of **3** (0.16 mM) upon addition of 0.16 mM solution of 4-OAc-DTBP in acetonitrile at –45 °C. (b) Decay of **3** (0.16 mM) at 465 nm upon addition of 0.16 mM of 4-OAc-DTBP (Inset: Second-order fitting of the time trace at 465 nm at –45 °C).

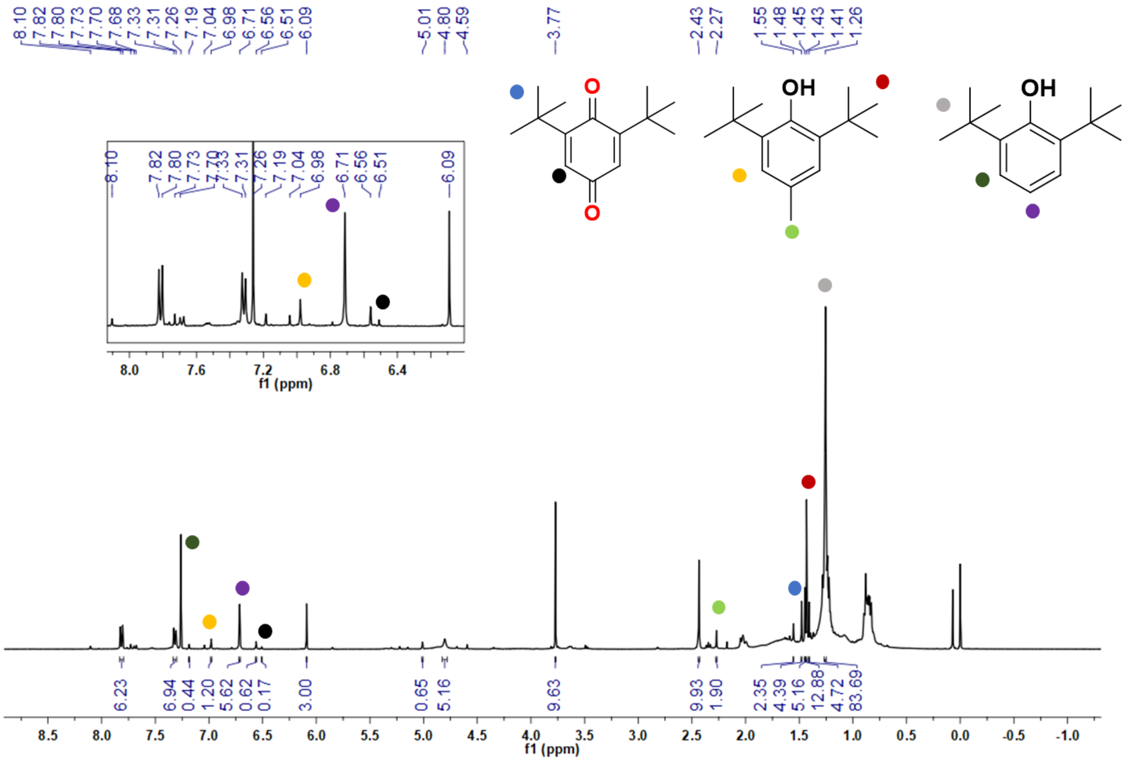

Figure S42. ^{1}H NMR spectrum ($CDCl_3$, 500 MHz, 25 °C) of the reaction mixture obtained after a reaction of **3** with 4-Me-DTBP in acetonitrile followed by an acid workup.

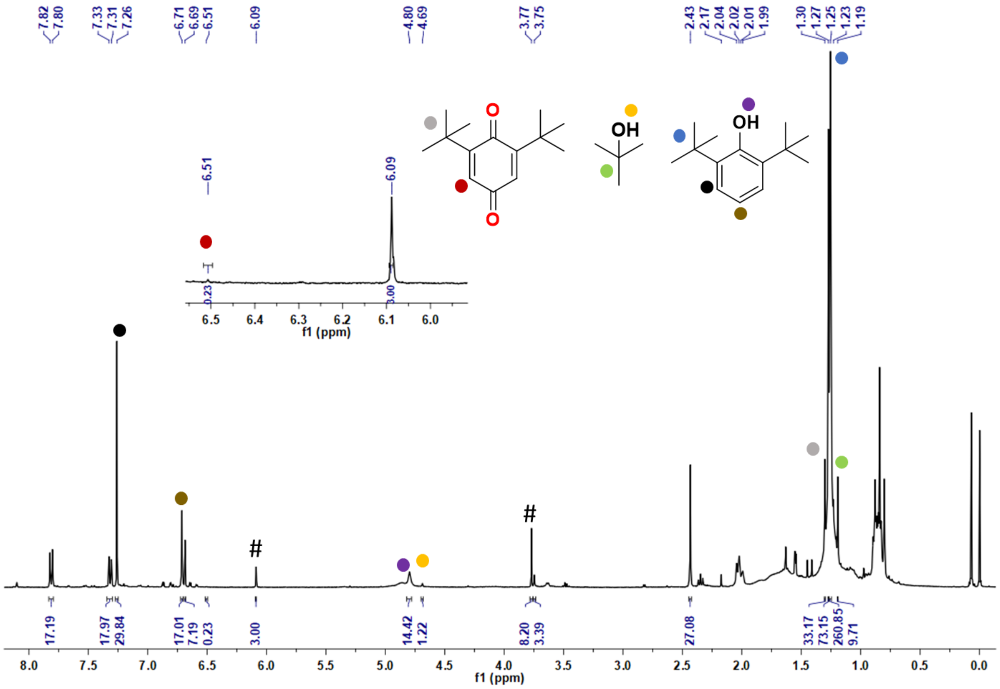

Figure S43. ^{1}H NMR spectrum ($CDCl_3$, 500 MHz, 25 °C) of the reaction mixture obtained after reaction of **3** with 4-H-DTBP in acetonitrile followed by an acid workup ($^{\#}$Trimethoxy benzene as internal standard).

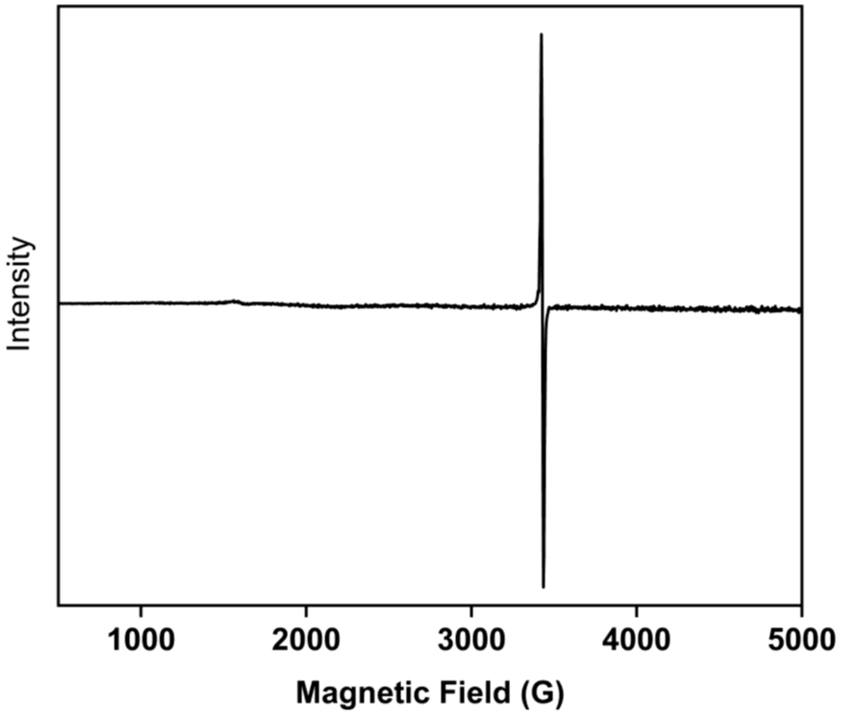

Figure S44. The X-band EPR spectrum of the reaction solution was obtained upon adding ten equiv. of 4-^{t}Bu-DTBP to an acetonitrile solution of **3** at –25 °C. The EPR data was recorded at the liquid nitrogen temperature.

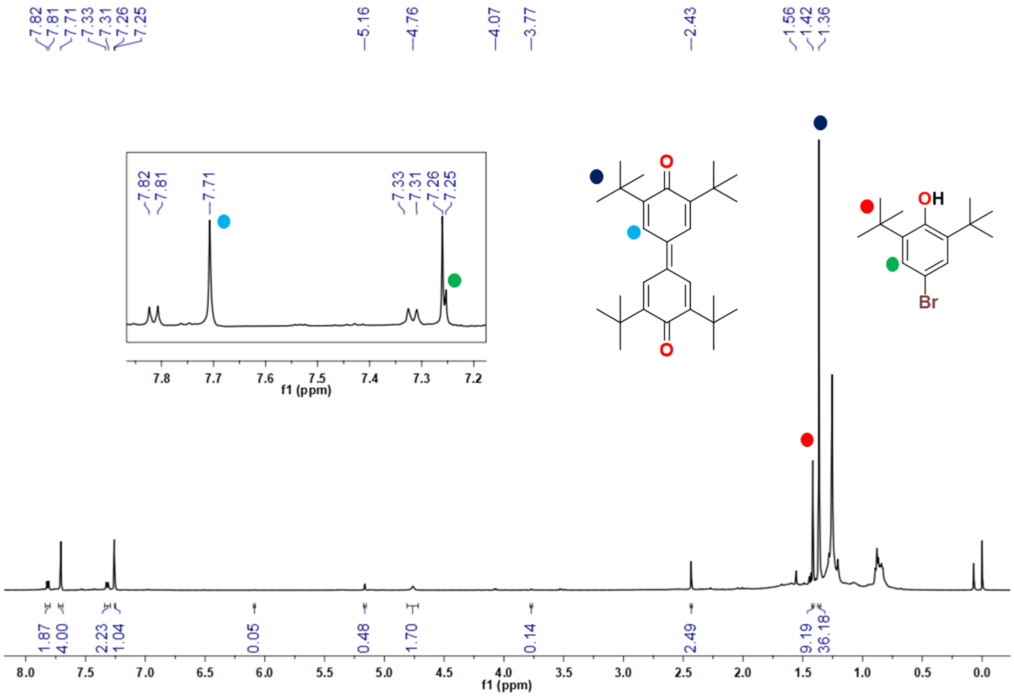

Figure S45. ^{1}H-NMR spectrum ($CDCl_3$, 500 MHz, 25 °C) of the reaction mixture obtained after a reaction of **3** with 4-Br-DTBP followed by an acid workup.

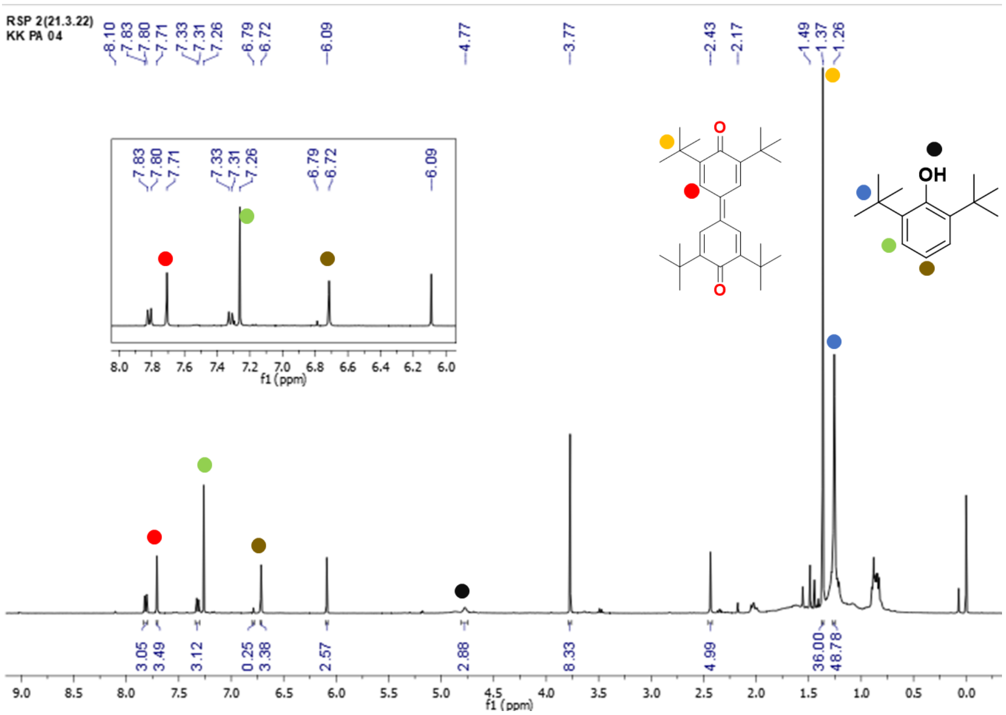

Figure S46. ^{1}H-NMR spectrum ($CDCl_3$, 500 MHz, 25 °C) of the reaction mixture obtained after a reaction of **3** with 2,6-DTBP followed by an acid workup.

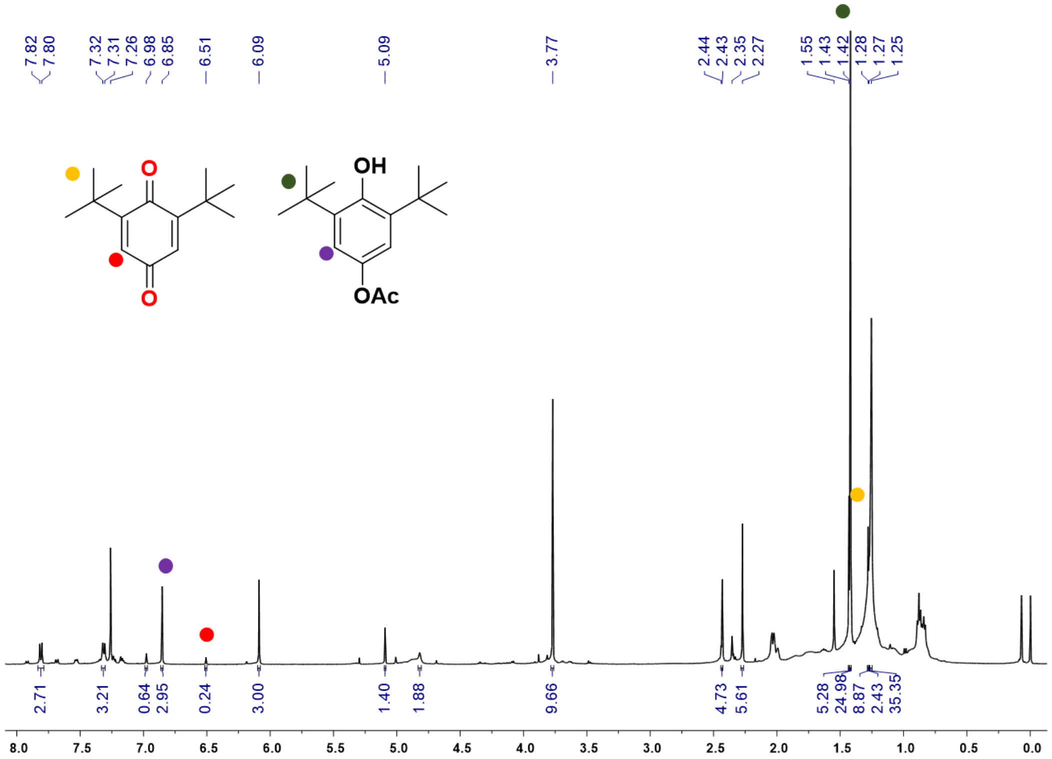

Figure S47. ^{1}H NMR spectrum ($CDCl_3$, 500 MHz, 25 °C) of the reaction mixture obtained after a reaction of **3** with 4-OAc-DTBP in acetonitrile followed by an acid workup.

Table S6. The second-order rate constant for the reaction of **3** with 4-X-2,6-di-*tert*-butylphenols (X= –OCH_3, –CH_3, –$C(CH_3)_3$, –H, –Br, and –OAc) at –45 °C and O–H bond dissociation energy of different phenols.

**Substrate**	**O–H BDE^{a}**	**E_{ox}^{b}**	σ_p^+	k_2^{c}	***log k$_2$***
2,6-di-*tert*-butyl-4-methoxyphenol	78.31	0.526	-0.78	30.10	1.44786
2,6-di-*tert*-butyl-4-methylphenol	81.02	0.81	-0.31	2.75	0.43917
2,6-di-*tert*-butyl-4-ethylphenol	80.1	0.88	-0.30	2.22	0.34537
2,4,6-tri-*tert*-butylphenol	81.24	0.927	-0.26	1.60	0.2052
2, 6-di-*tert*-butylphenol	82.8	1.074	0	3.11	0.48244
2,6-di-*tert*-butyl-4-acetoxyphenol	84.4	0.888	-0.19	13.5	1.13
2,6-di-*tert*-butyl-4-bromophenol	–	1.1	0.15	69.66	1.84

^{a}kcal/mol, ^{b}V vs. Fc^{+}/Fc, $^{c}M^{-1}$ s^{-1}.

σ_p^+ values were taken from reference [28].

Table S7. The yield of the products formed for the reaction of **3** with 4-X-DTBP, (4-X-$C_6H_4)_3P$, and other substrates.

**Substrate**	**Product**	**Yield (%)**
4-OMe-DTBP	2,6-Di-*tert*-butyl-P-benzoquinone	24
4-Me-DTBP	2,6-Di-*tert*-butyl-P-benzoquinone	13
4-^{t}Bu-DTBP	2,6-Di-*tert*-butyl-P-benzoquinone	12
4-H-DTBP	3,3',5,5'-tetra-*tert*-butyldiphenoquinone	22
4-Br-DTBP	3,3',5,5'-tetra-*tert*-butyldiphenoquinone	40
4-OAc-DTBP	2,6-di-*tert*-butyl-P-benzoquinone	10
BNAH	BNA^+	96
9,10-DHA	Anthracene	50
PPh_3	Ph_3PO	25
(4-OMe-$C_6H_4)_3P$	4-OMe-$C_6H_4)_3PO$	44
(4-OMe-$C_6H_4)_3C$•	(4-OMe-$C_6H_4)_3C(OH)$	67

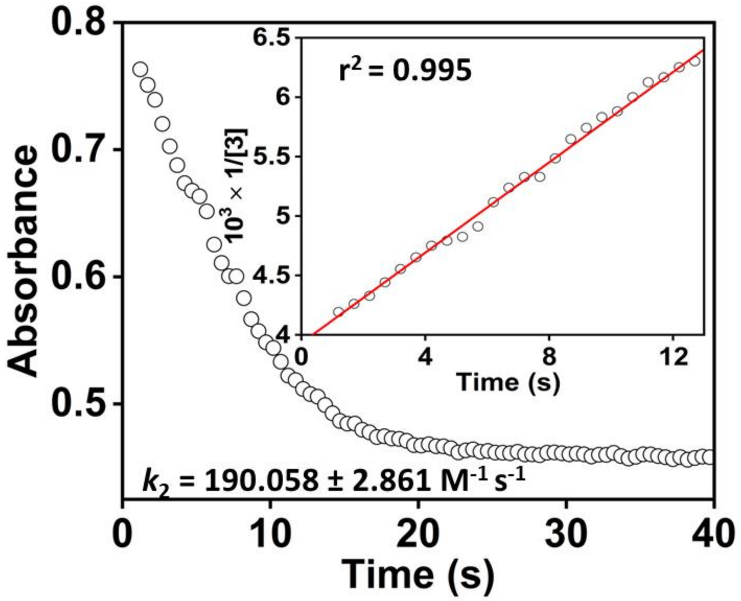

Figure S48. Decay of **3** (0.25 mM) at 465 nm upon addition of 0.25 mM of 4-OMe-DTBP at –25 °C (Inset: Second-order fitting of the time trace at 465 nm at –25 °C of the reaction of **3** (0.25 mM) with 4-OMe-DTBP (0.25 mM) in acetonitrile).

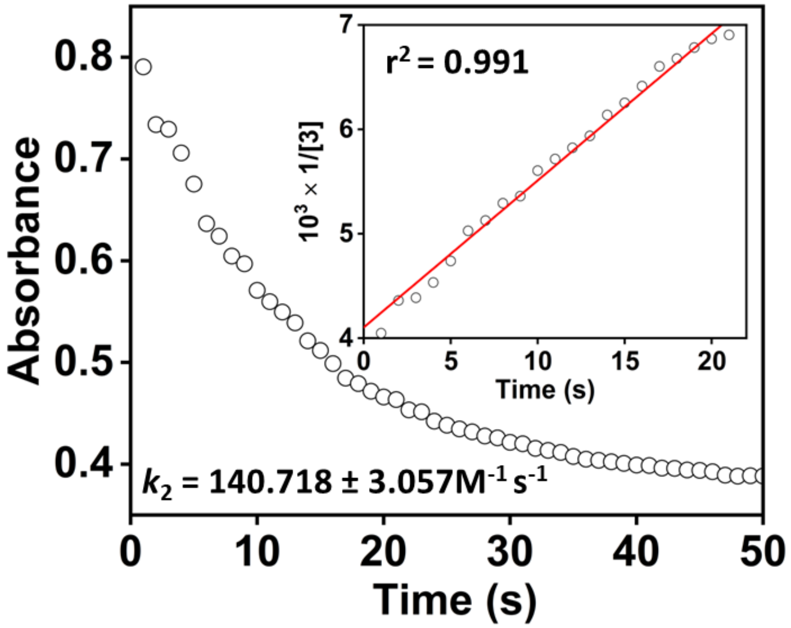

Figure S49. Decay of **3** (0.25 mM) at 465 nm upon addition of 0.25 mM of 4-OMe-DTBP-*d* (Inset: Second-order fitting of the time trace at 465 nm at –25 °C for the reaction of **3** (0.25 mM) with 4-OMe-DTBP-*d* (0.25 mM) in acetonitrile).

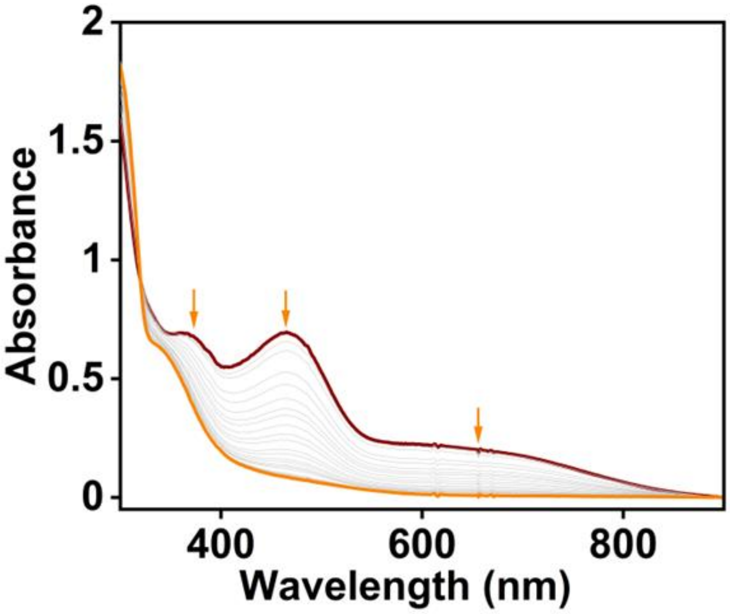

Figure S50. Change of single spectrum of **3** (0.22 mM) upon addition of 3.6 mM solution of 4-Me-DTBP in acetonitrile at –25 °C.

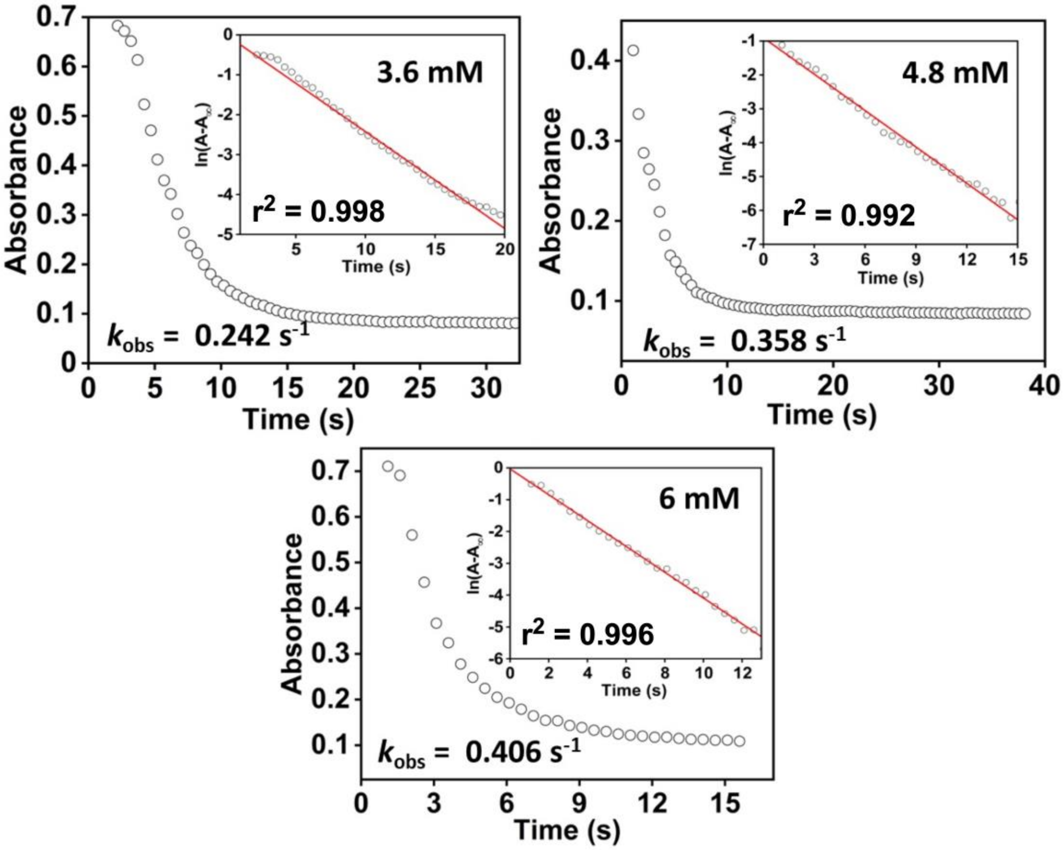

Figure S51. Decay of **3** (0.22 mM) at 465 nm upon addition of different concentrations (3.6–6 mM) of 4-Me-DTBP at –25 °C (Inset: determination of k_{obs} value).

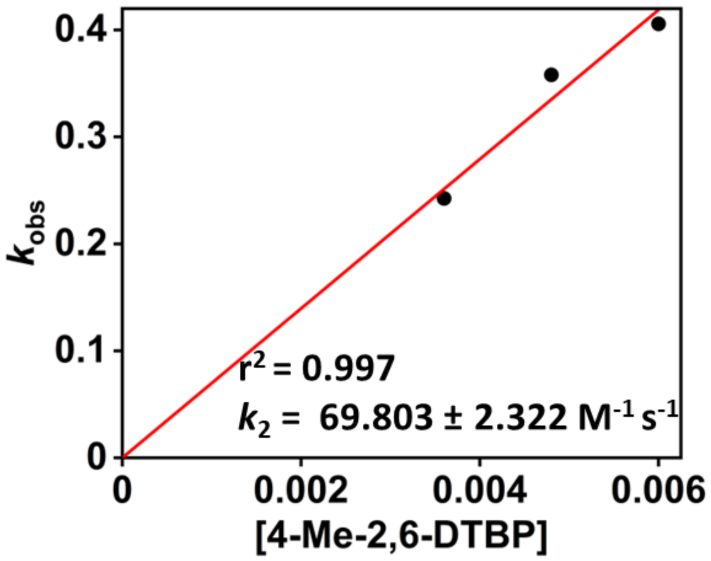

Figure S52. A plot of k_{obs} vs. [4-Me-DTBP]. The k_{obs} values were obtained by reacting **3** with different concentrations of 4-Me-DTBP at –25 °C in acetonitrile. The second-rate constant was obtained from the slope of the plot.

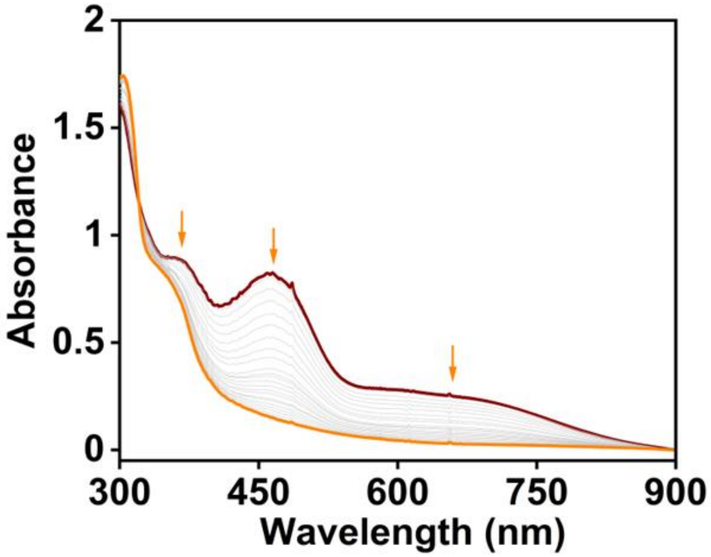

Figure S53. Change of single spectrum of **3** (0.25 mM) upon addition of 2.4 mM solution of 4-Et-DTBP in acetonitrile at –25 °C.

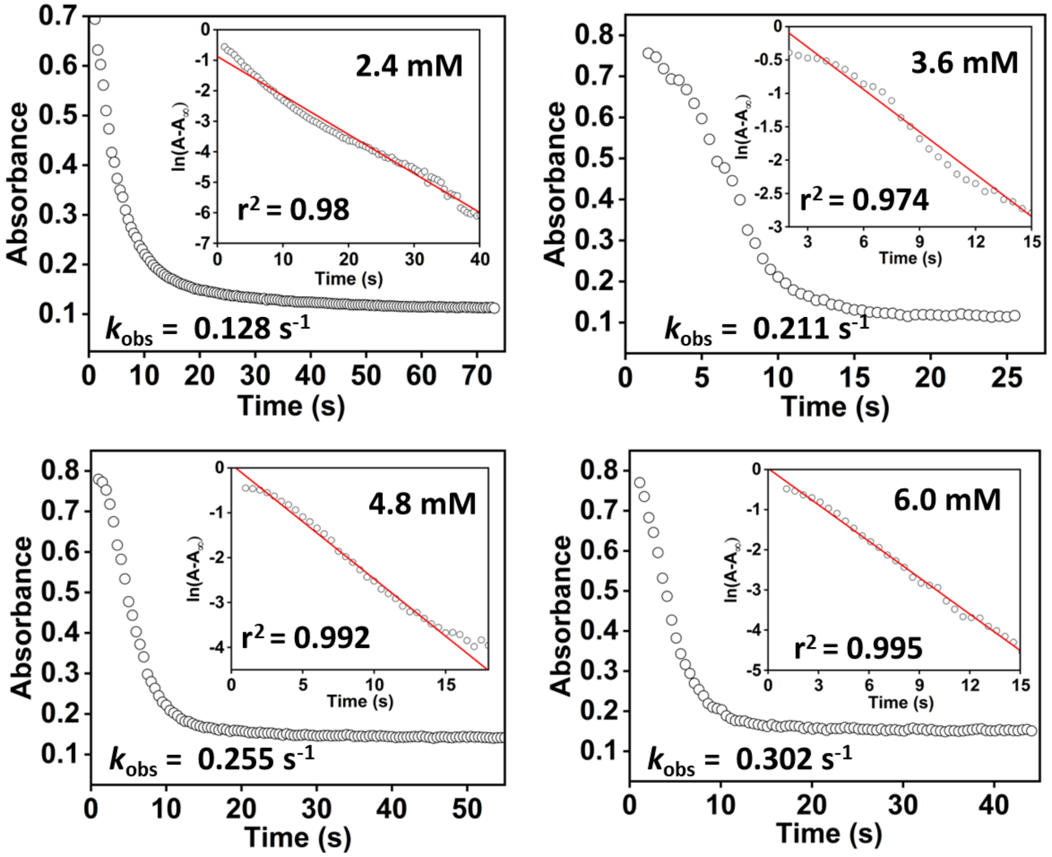

Figure S54. Decay of **3** (0.25 mM) at 465 nm upon addition of different concentrations (2.4–6 mM) of 4-Et-DTBP –25 °C (Inset: determination of k_{obs} value).

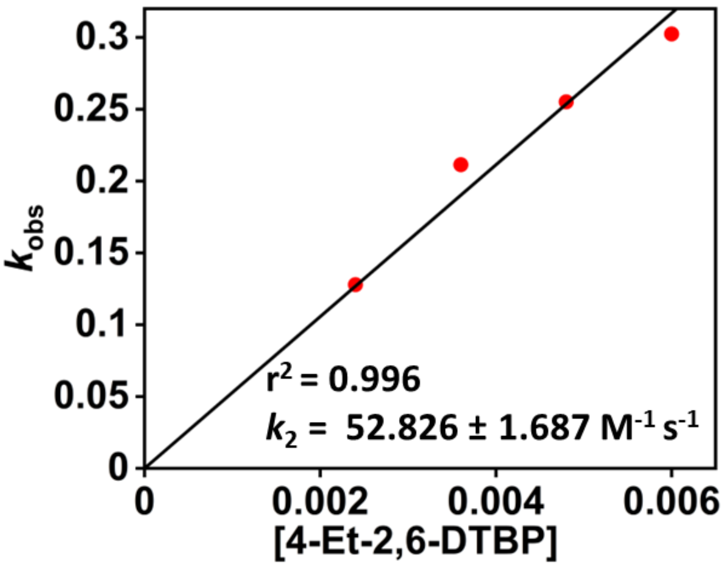

Figure S55. A plot of k_{obs} vs. [4-Et-DTBP]. The k_{obs} values were obtained by reacting **3** with different concentrations of 4-Et-DTBP at –25 °C in acetonitrile. The second-order rate constant was obtained from the slope of the plot.

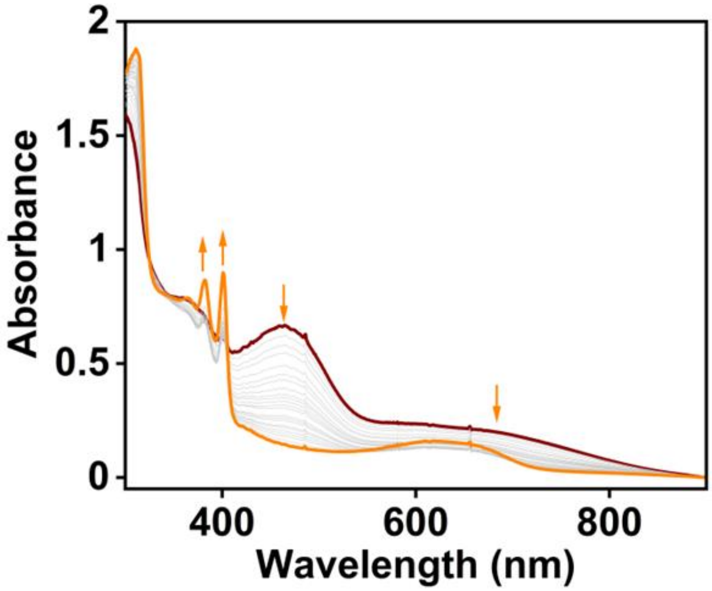

Figure S56. Change of single spectrum of **3** (0.22 mM) upon addition of 2.4 mM solution of 4-^{t}Bu-DTBP in acetonitrile at –25 °C.

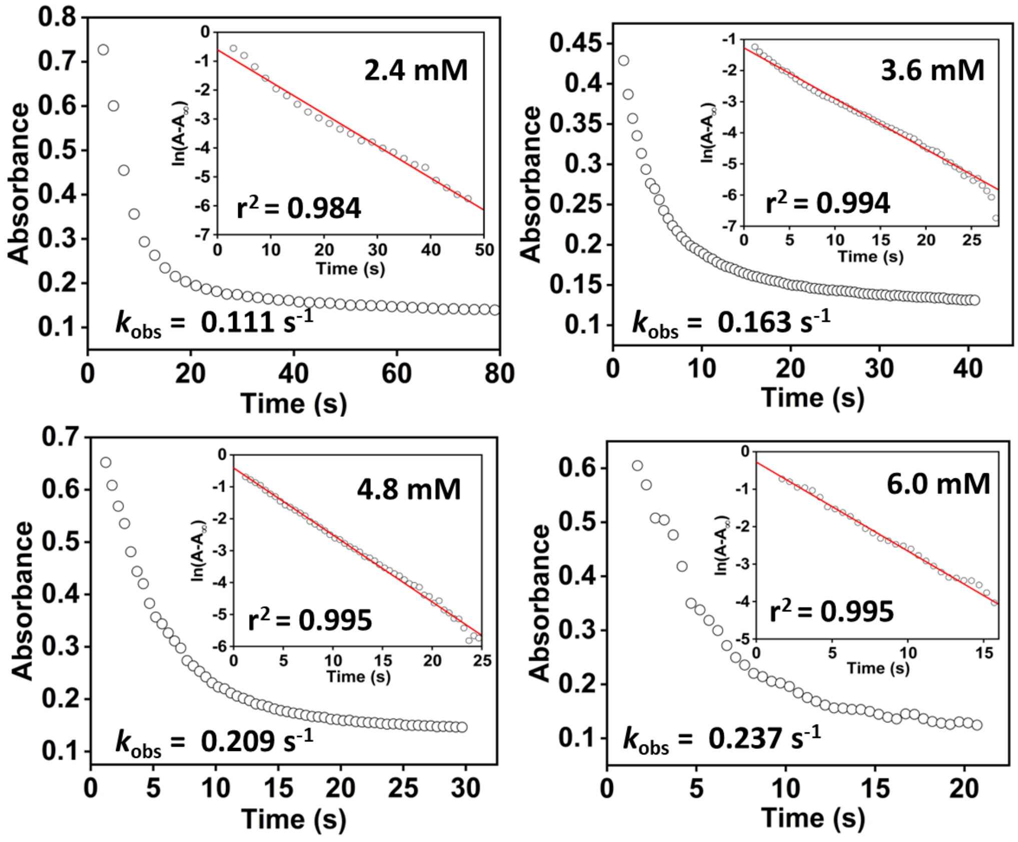

Figure S57. Decay of **3** (0.22 mM) at 465 nm upon addition of different concentrations (2.4–6 mM) of 4-^{t}Bu-DTBP –25 °C (Inset: determination of k_{obs} value).

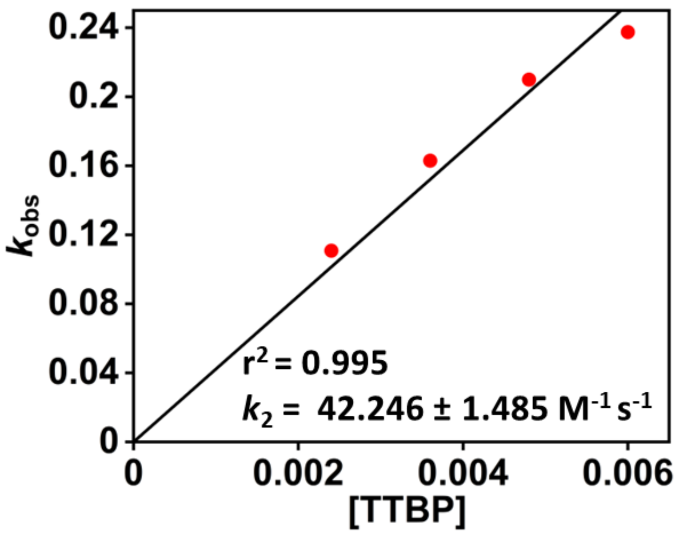

Figure S58. A plot of k_{obs} vs. [4-^{t}Bu-DTBP]. The k_{obs} values were obtained by reacting **3** with different concentrations of 4-^{t}Bu-DTBP at −25 °C in acetonitrile. The second-order rate constant was obtained from the slope of the plot.

Table S8. Second-order rate constant (k_2) values for the reaction of intermediate **3** and **2** with 4-Et-DTBP at different temperatures.

**Temperature**	**k_2 (M^{-1} s^{-1}) using 3**	**k_2 (M^{-1} s^{-1}) using 2**	**Ratio of $k_2(3)/k_2(2)$**
−25 °C	52.826	0.416	126.98
−30 °C	20.991	0.302	69.5
−35 °C	10.090	0.229	44.06
−40 °C	6.090	0.177	34.4
−45 °C	2.215	-	-

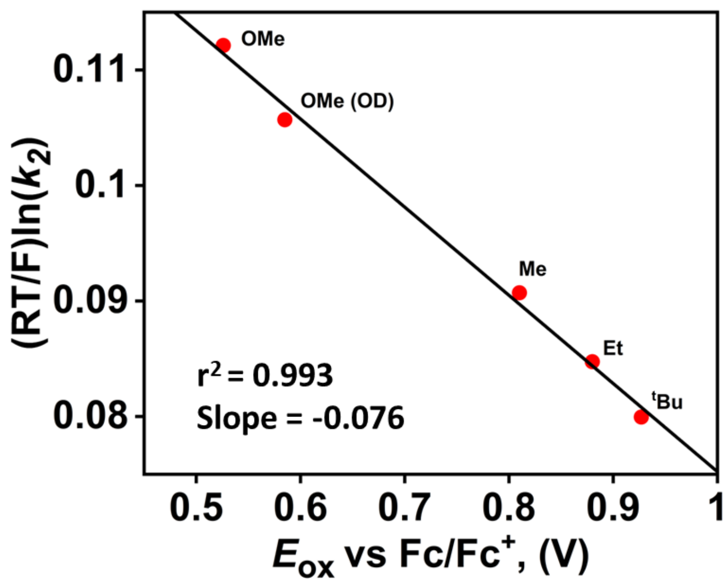

Figure S59. A plot of (RT/F)lnk_2 vs. E_{ox} (vs. Fc^+/Fc) for 4-X-DTBP at –25 °C in acetonitrile.

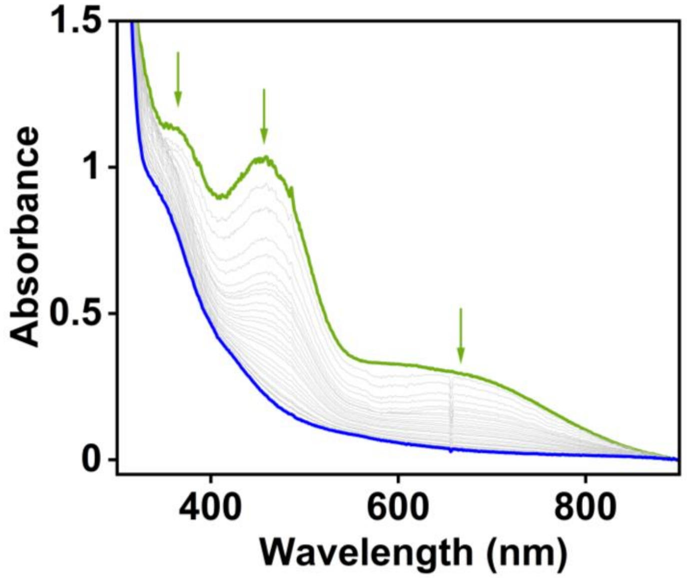

Figure S60. Change of single spectrum of **5** (0.32 mM) upon addition of 8.2 mM solution of 4-Me-DTBP in acetonitrile at –25 °C.

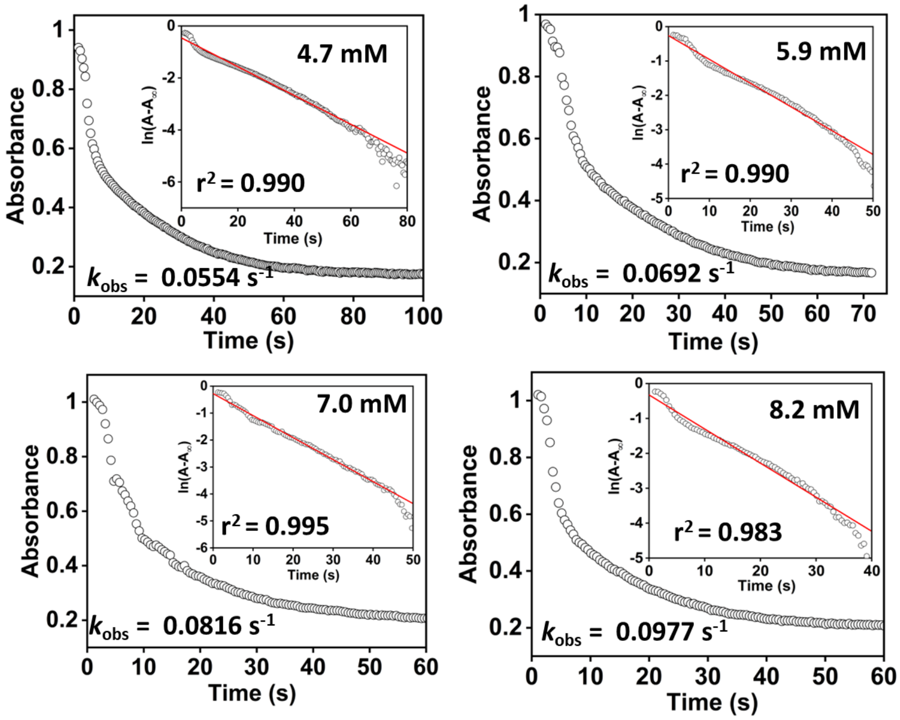

Figure 61. Decay of **5** (0.32 mM) was monitored at 465 nm upon the addition of different concentrations (4.7–8.2 mM) of 4-Me-DTBP at −25 °C (Inset: determination of k_{obs} value).

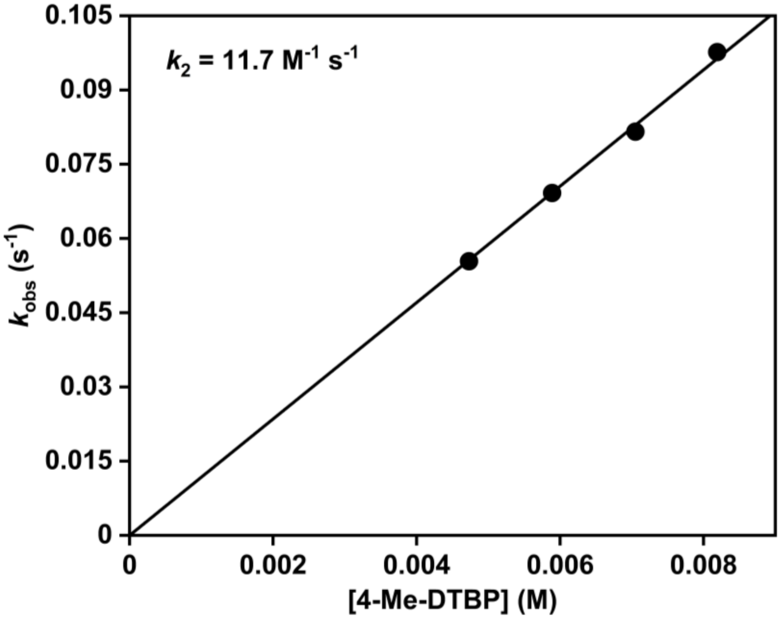

Figure S62. A plot of k_{obs} vs. [4-Me-DTBP]. The k_{obs} values were obtained by reacting **5** with different concentrations of 4-Me-DTBP at −25 °C in acetonitrile. The second-order rate constant was obtained from the slope of the plot.

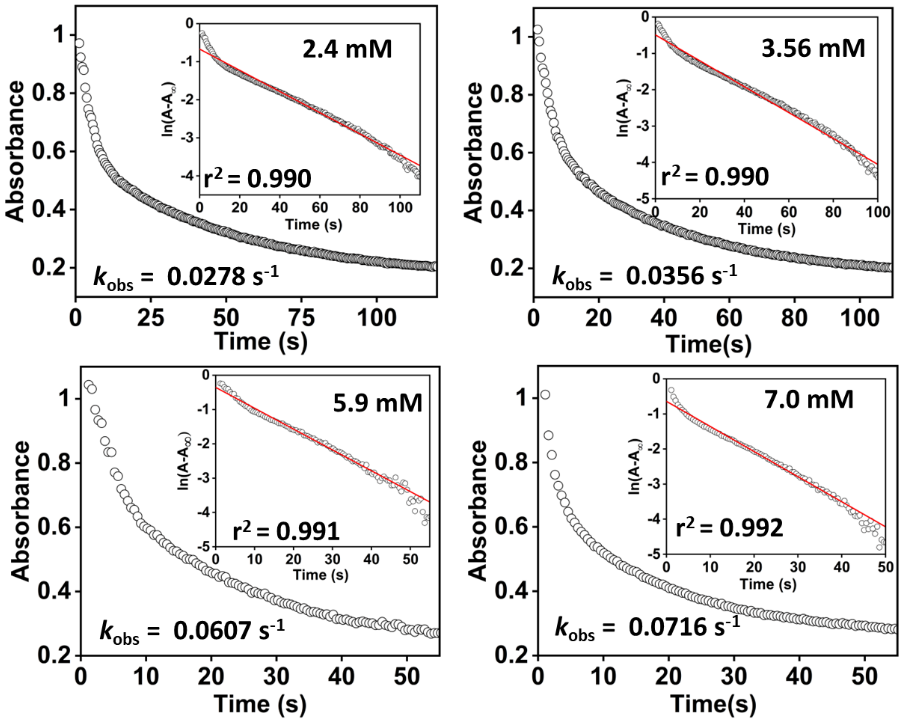

Figure 63. Decay of **5** (0.32 mM) was monitored at 465 nm upon the addition of different concentrations (2.4–7.0 mM) of 4-Et-DTBP –25 °C (Inset: determination of k_{obs} value).

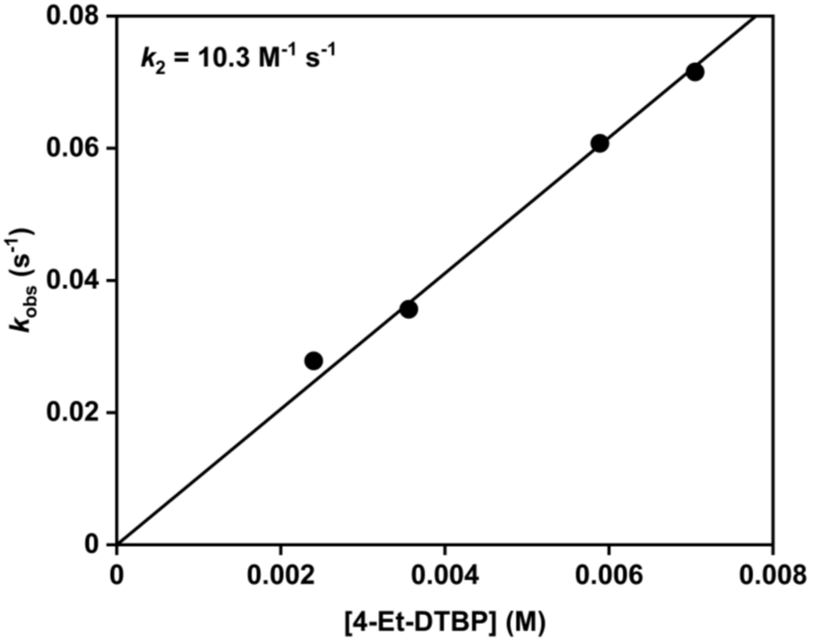

Figure S64. A plot of k_{obs} vs. [4-Et-DTBP]. The k_{obs} values were obtained by reacting **5** with different concentrations of 4-Et-DTBP at –25 °C in acetonitrile. The second-order rate constant (k_2) was obtained from the slope of the plot.

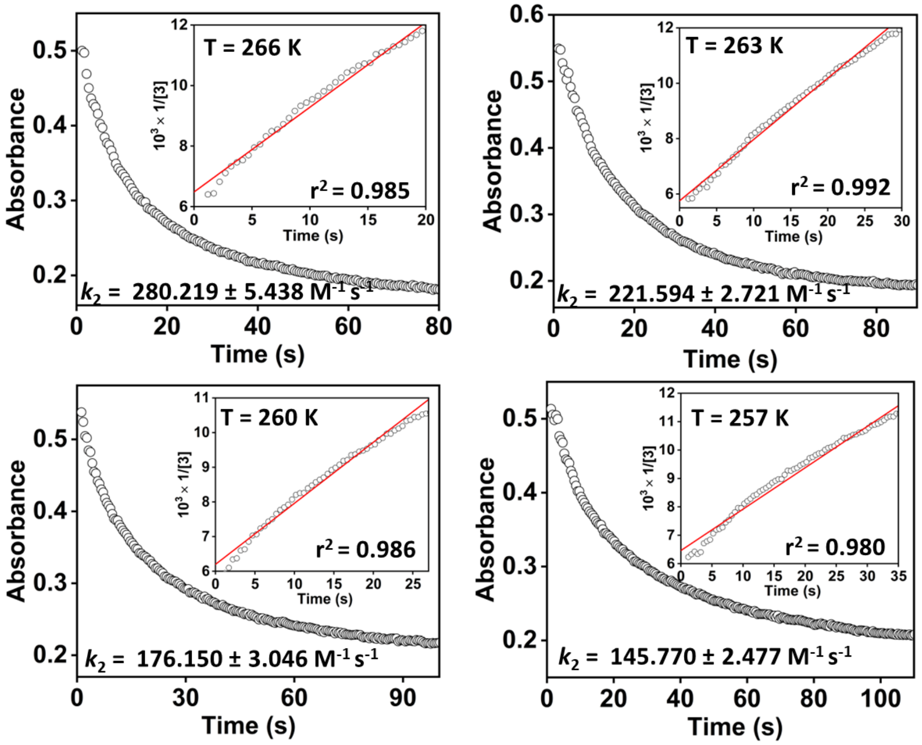

Figure S65. Decay of **3** (0.2 mM) at 465 nm upon addition of BNAH (0.2 mM) at different temperatures. Inset: Second-order fitting of the time trace at 465 nm at different temperatures of the reaction of **3** (0.2 mM) with BNAH (0.2 mM) acetonitrile.

Table S9. Second-order rate constants (k_2) for the reaction of intermediate **3** with BNAH at different temperatures.

Temperature (K)	k_2 (M^{-1} s^{-1})
−16 °C	145.77
−13 °C	176.15
−10 °C	221.59
−7 °C	280.22

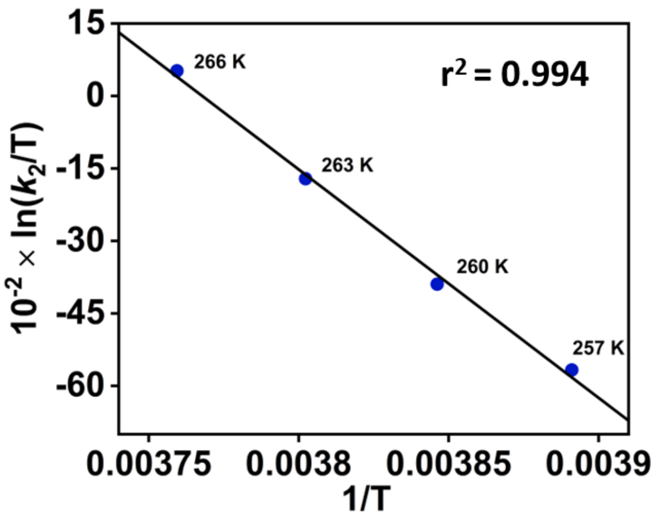

Figure S66. Eyring plot for the reaction of **3** with BNAH over a temperature range of −7 °C to −16 °C.

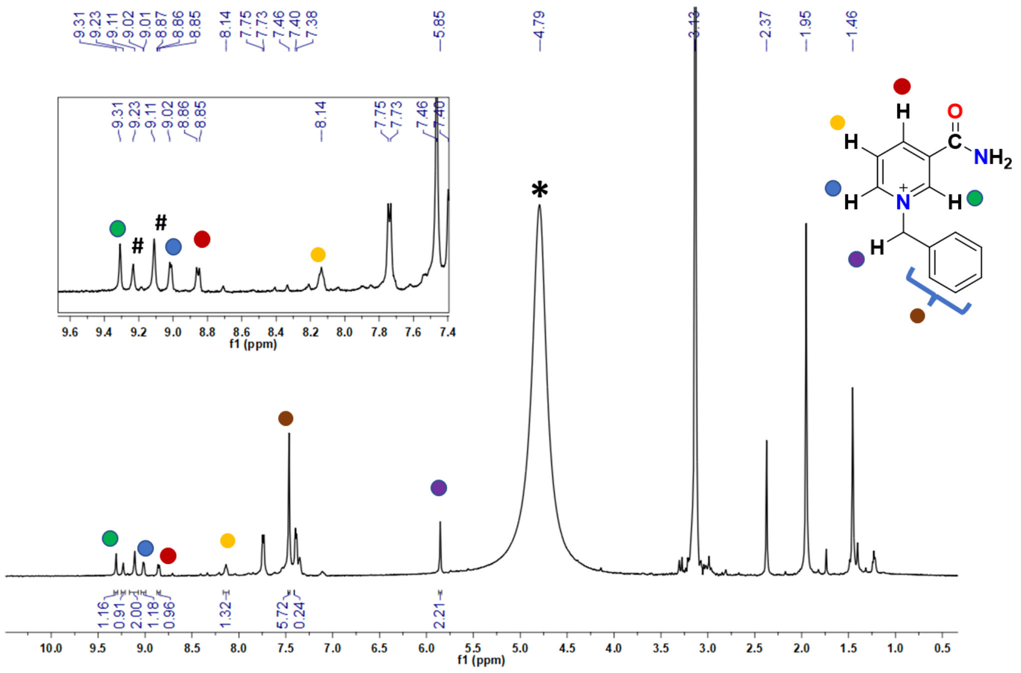

Figure S67. ^{1}H NMR spectrum (D_2O, 500 MHz, 25 °C) of the reaction mixture obtained after reaction of **3** with BNAH in acetonitrile ([#]3,5-Dinitrobenzoic acid as internal standard, * solvent residual peak).

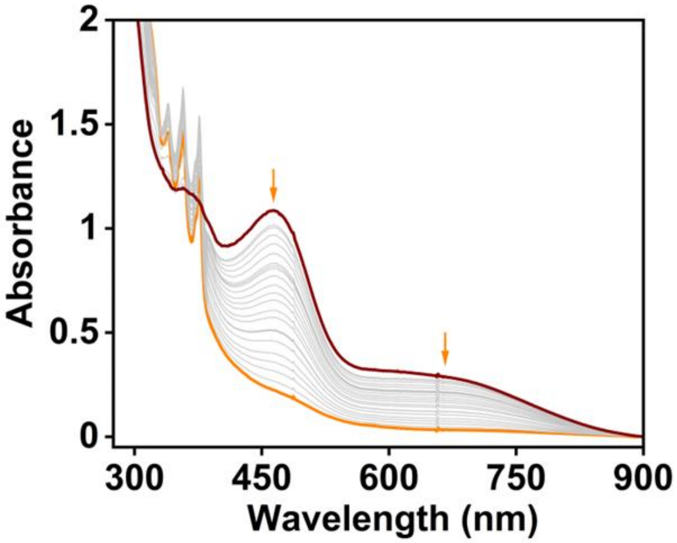

Figure S68. Change of single spectrum of **3** (0.30 mM) upon addition of 11.9 mM solution of 9,10-DHA in acetonitrile at –10 °C.

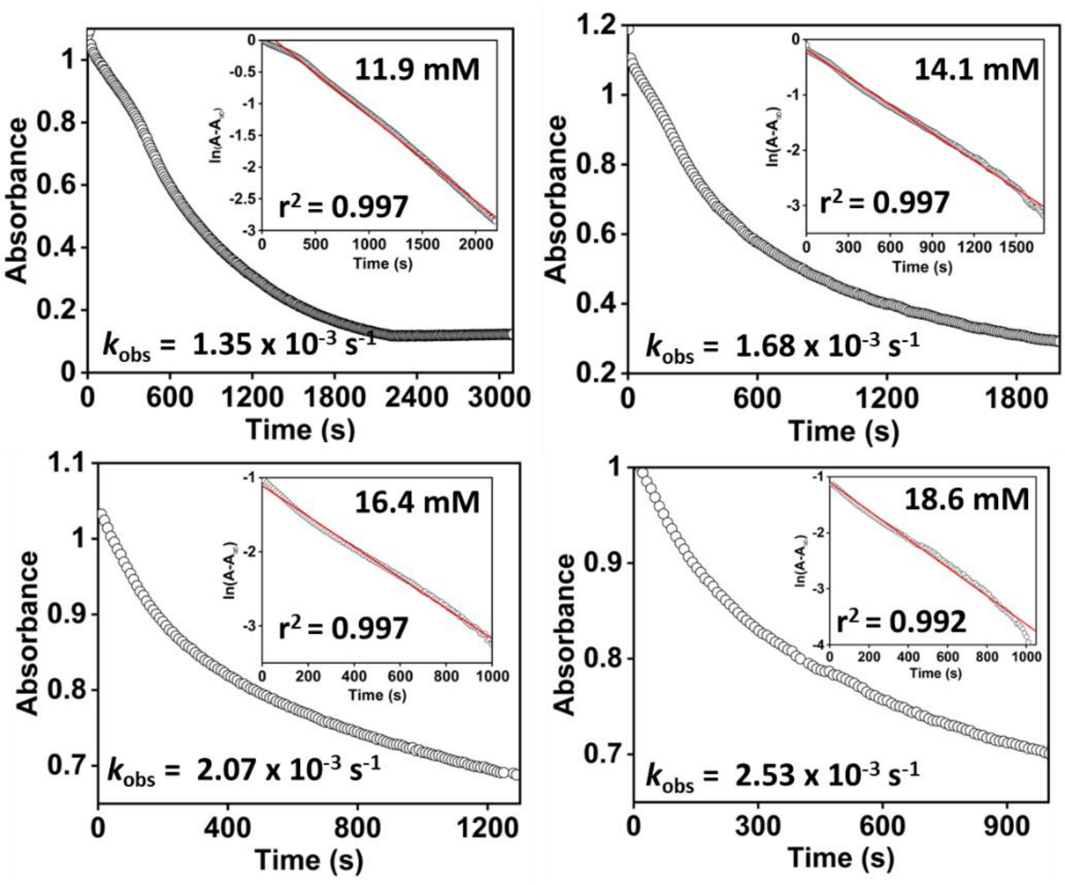

Figure S69. Decay of **3** (0.30 mM) at 465 nm upon addition of different concentrations (11.9–18.6 mM) of 9,10-DHA at –10 °C (Inset: determination of k_{obs} value).

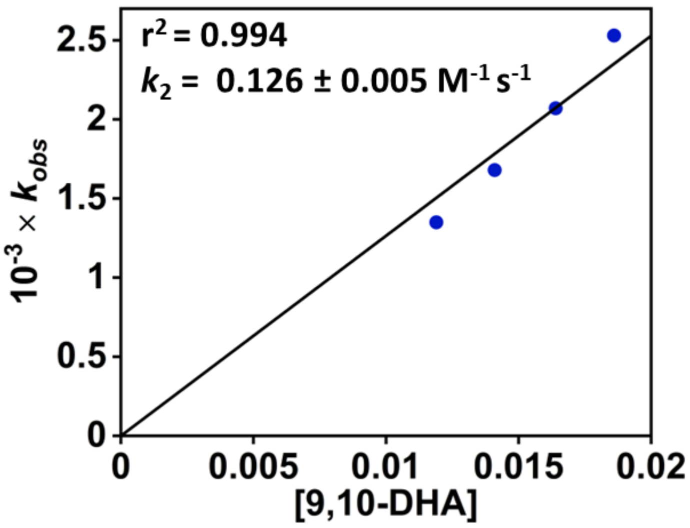

Figure S70. A plot of k_{obs} vs. [9,10-DHA]. The k_{obs} values were obtained by reacting **3** with different concentrations of 9,10-DHA at –10 °C in acetonitrile. The second-order rate constant was obtained from the slope of the plot.

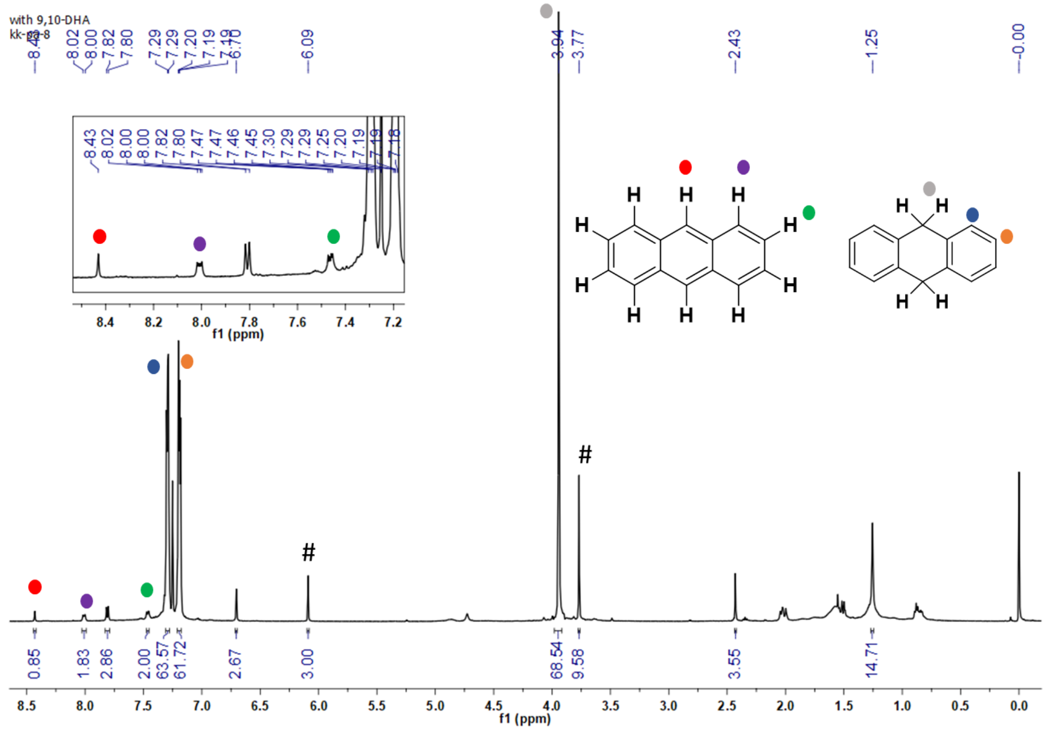

Figure S71. ^{1}H NMR spectrum ($CDCl_3$, 500 MHz, 25 °C) of the reaction mixture obtained after a reaction of **3** with 9,10-DHA in acetonitrile followed by an acid workup.

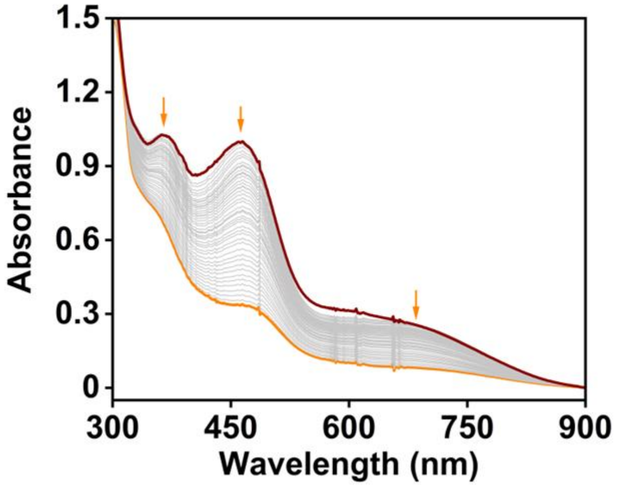

Figure S72. Change of single spectrum of **3** (0.3 mM) upon addition of 11.9 mM solution of 1,4-cyclohexadiene in acetonitrile at –10 °C.

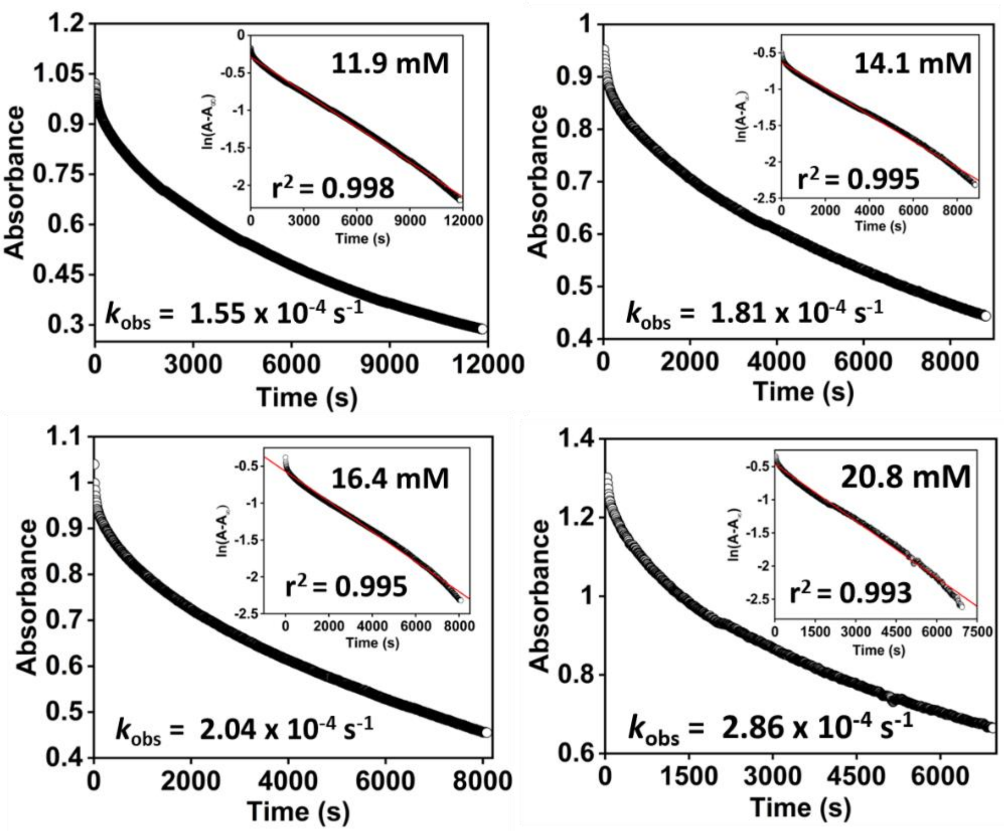

Figure S73. Decay of **3** (0.3 mM) at 465 nm upon addition of different concentrations (11.9–20.8 mM) of 1,4-cyclohexadiene at –10 °C (Inset: determination of k_{obs} value).

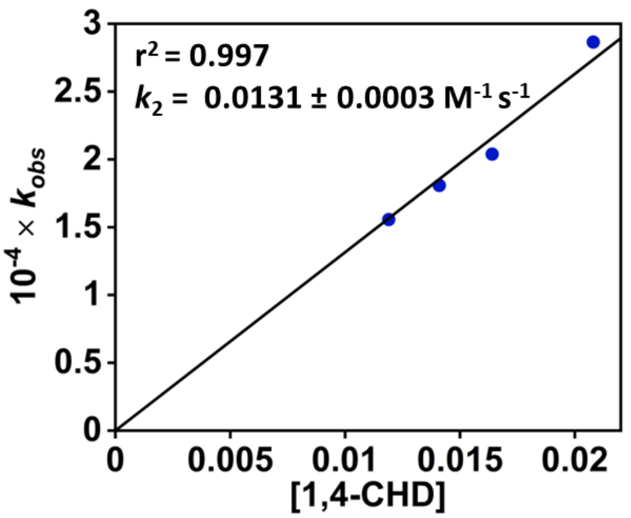

Figure S74. A plot of k_{obs} vs. [1,4-CHD]. The k_{obs} values were obtained by reacting **3** with different concentrations of 1,4-CHD in acetonitrile at –10 °C. The second-order rate constant was obtained from the slope of the plot.

Table S10. The second-order rate constant (k_2) for the reaction of **3** with BNAH, 9,10-DHA, and 1,4-CHD substrates.

**Substrate**	**C–H BDE (kcal. mol^{-1})**	**k_2 (M^{-1} s^{-1})**	***logk_2′***
BNAH	70.7	221.594	2.0437
9,10-DHA	77	0.13	-1.5
1,4-CHD	78	0.013	-2.4828

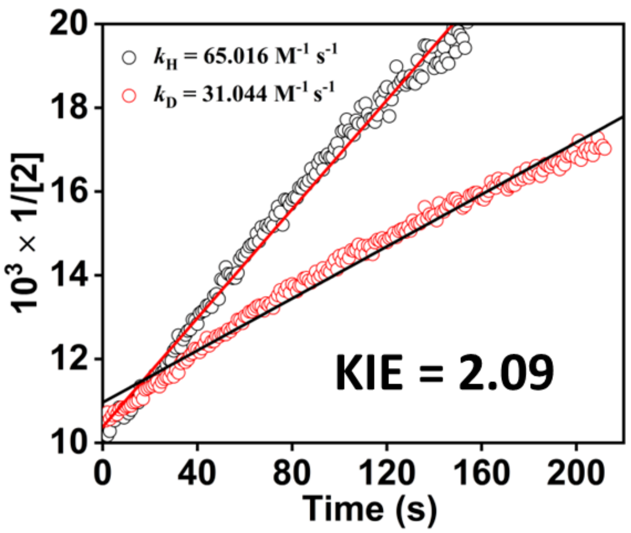

Figure S75. A plot of 1/[**2**] versus time (s) for the reaction of **2** with BNAH or BNAD at –10 °C. The k_2^H and k_2^D values were determined from the slope of this plot.

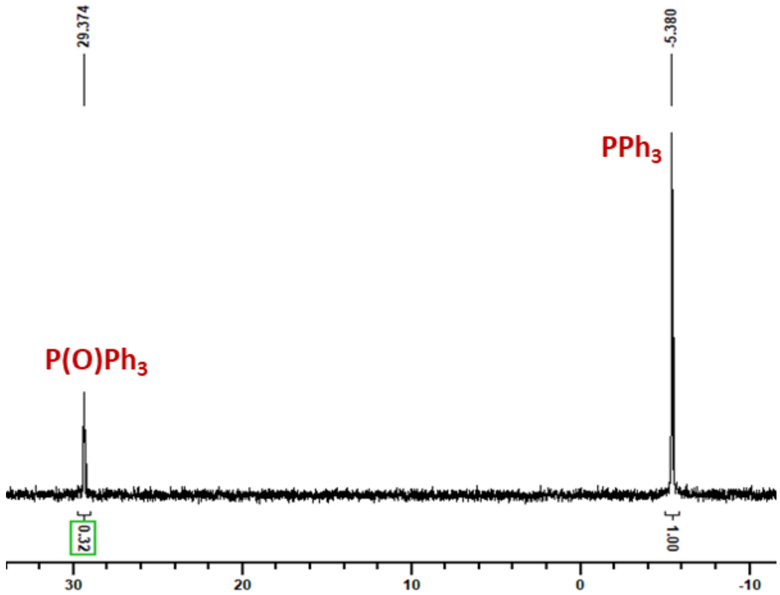

Figure S76. ^{31}P NMR spectrum ($CDCl_3$, 202 MHz, 25 °C) of the reaction mixture obtained after reaction of **3** with PPh_3 in acetonitrile.

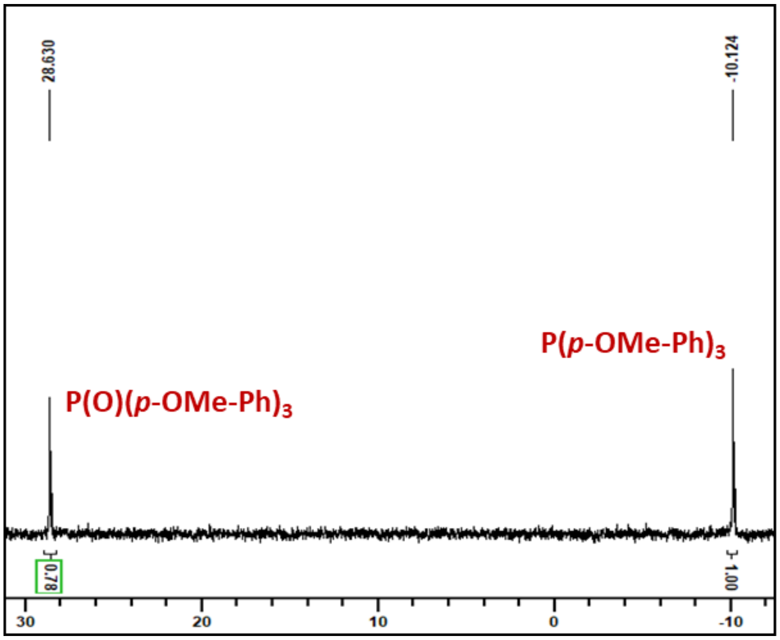

Figure S77. ^{31}P NMR spectrum ($CDCl_3$, 202 MHz, 25 °C) of the reaction mixture obtained after a reaction of **3** with (4-OMe-C_6H_4)$_3$P in acetonitrile.

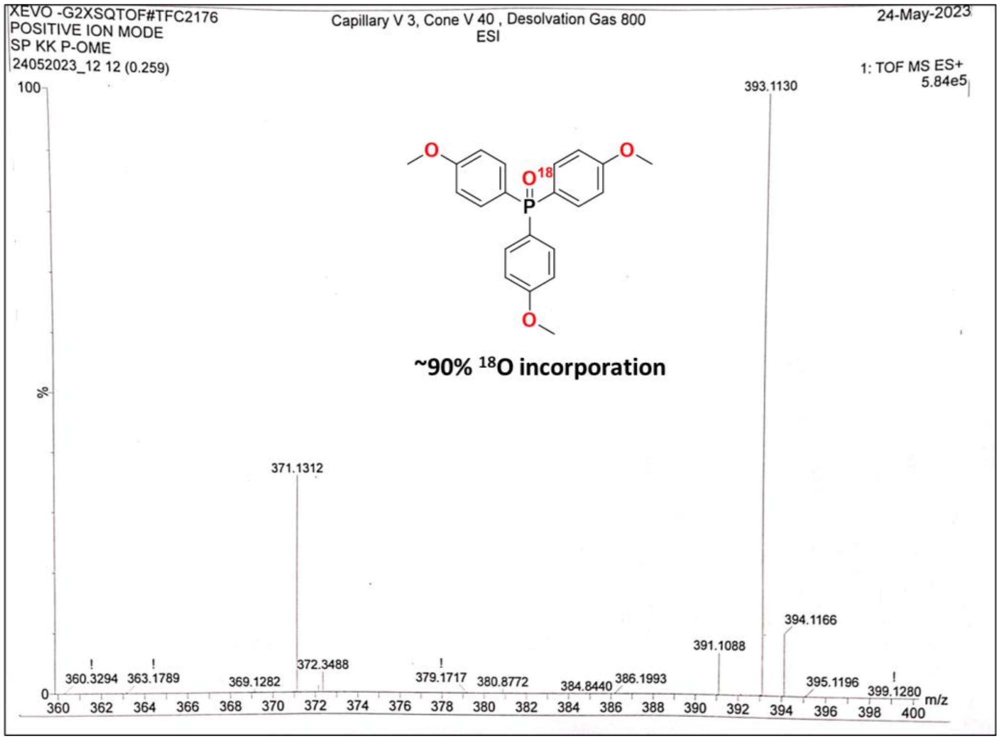

Figure S78. The ESI-mass spectrum of the reaction solution obtained upon reacting intermediate [(HMPAB)$Fe^{IV}O^{18}H$]$^{-}$ with (4-OMe-C_6H_4)$_3$P in acetonitrile.

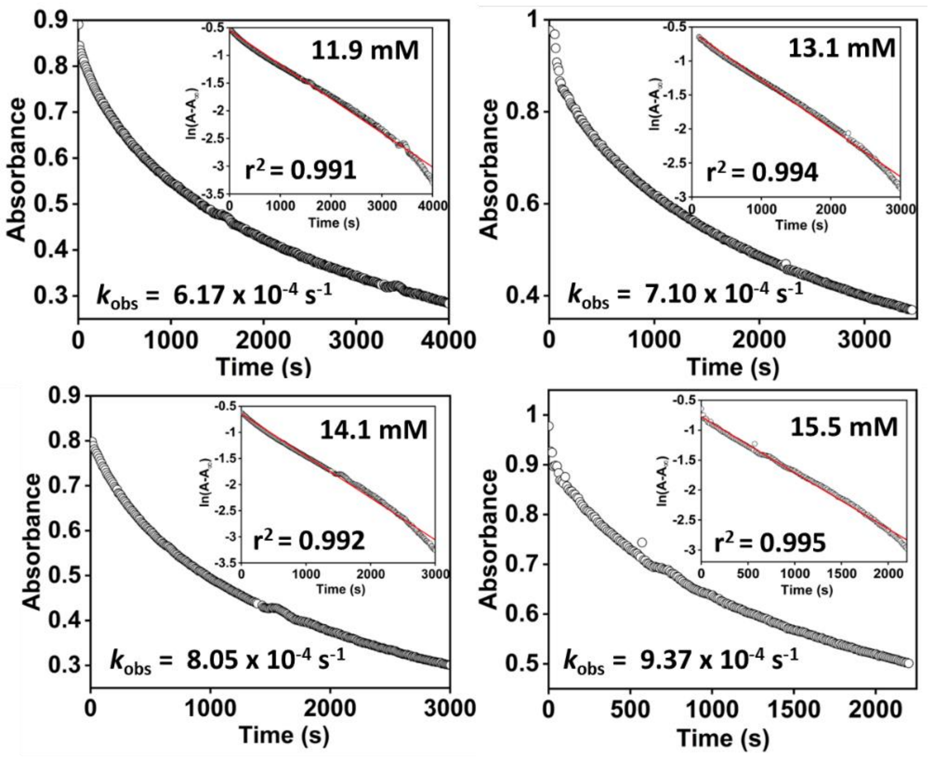

Figure S79. Decay of **3** (0.28 mM) at 465 nm upon addition of different concentrations (11.9–15.5 mM) of PPh_3 at –10 °C (Inset: determination of k_{obs} value).

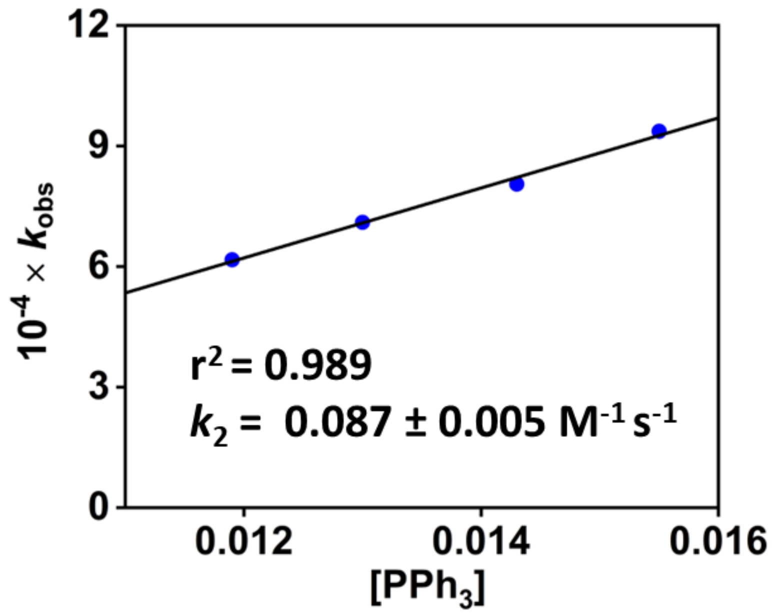

Figure S80. A plot of k_{obs} vs. [PPh_3]. The k_{obs} values were obtained by reacting **3** with different concentrations of PPh_3 at –10 °C in acetonitrile. The second-order rate constant value was obtained from the slope of the plot.

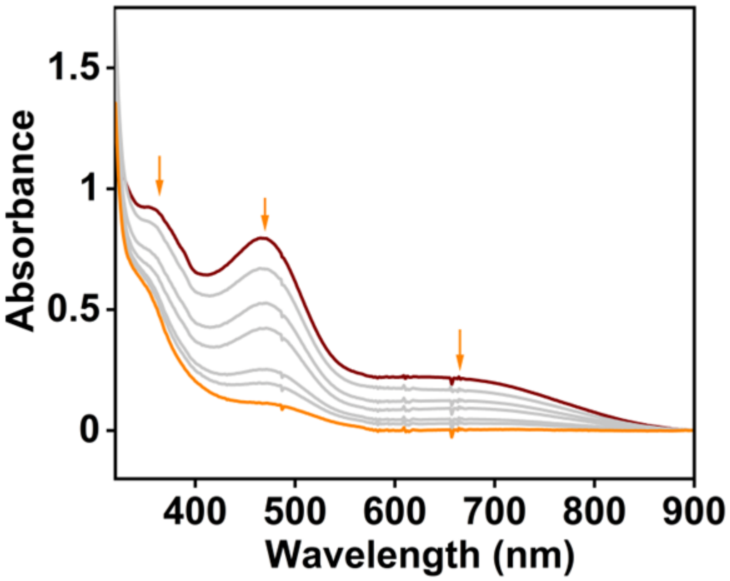

Figure S81. Change of single spectrum of **3** (0.22 mM) upon addition of a 2.4 mM solution of (4-Me-$C_6H_4)_3P$ in acetonitrile at –10 °C.

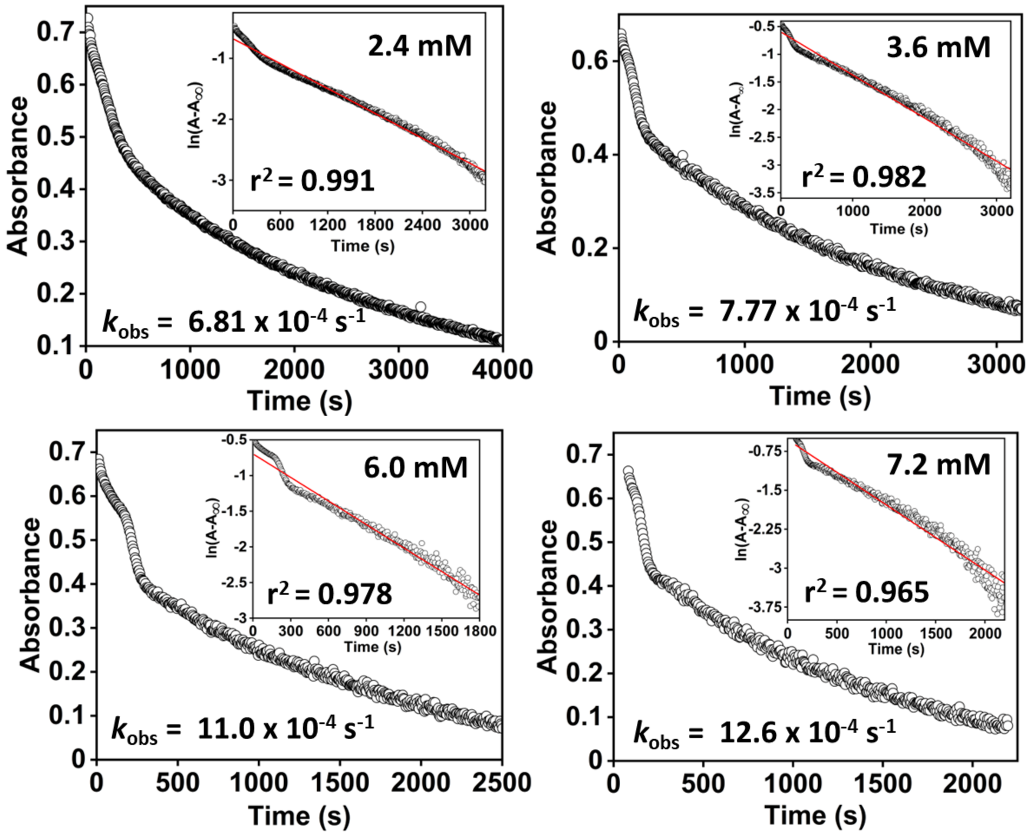

Figure S82. Decay of **3** (0.22 mM) at 465 nm upon addition of different concentrations (2.4 -7.2 mM) of (4-Me-$C_6H_4)_3P$ at –10 °C (Inset: determination of k_{obs} value).

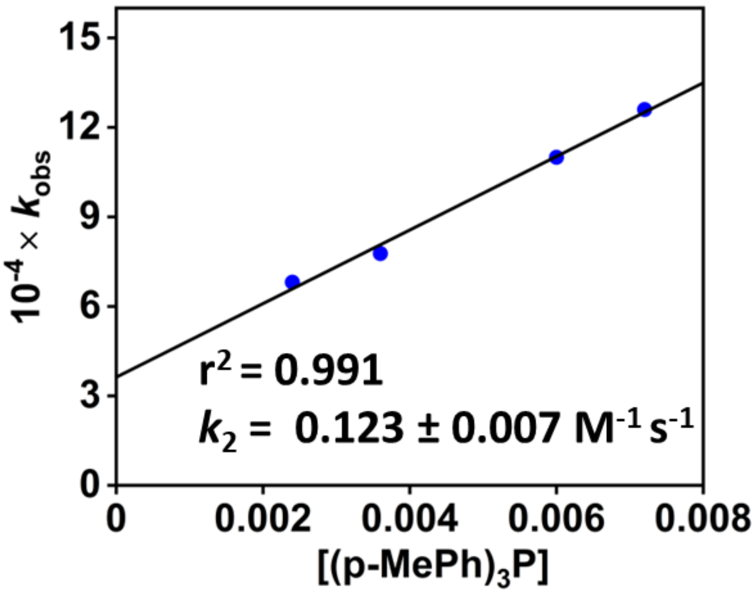

Figure S83. A plot of k_{obs} vs. [(4-Me-C_6H_4)$_3$P]. The k_{obs} values were obtained by reacting **3** with different concentrations of (4-Me-C_6H_4)$_3$P in acetonitrile at –10 °C. The second-order rate constant was obtained from the slope of the plot.

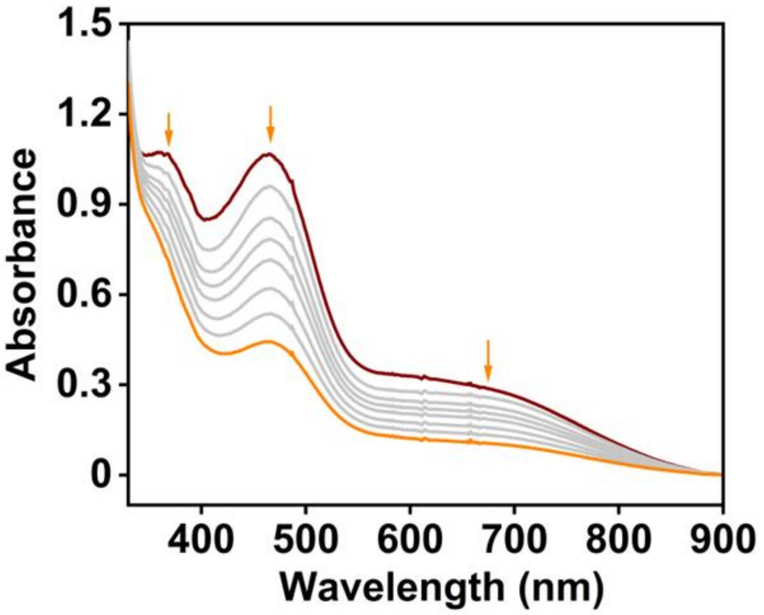

Figure S84. Change of single spectrum of **3** (0.30 mM) upon addition of 2.4 mM solution of (4-Cl-C_6H_4)$_3$P in acetonitrile at –10 °C.

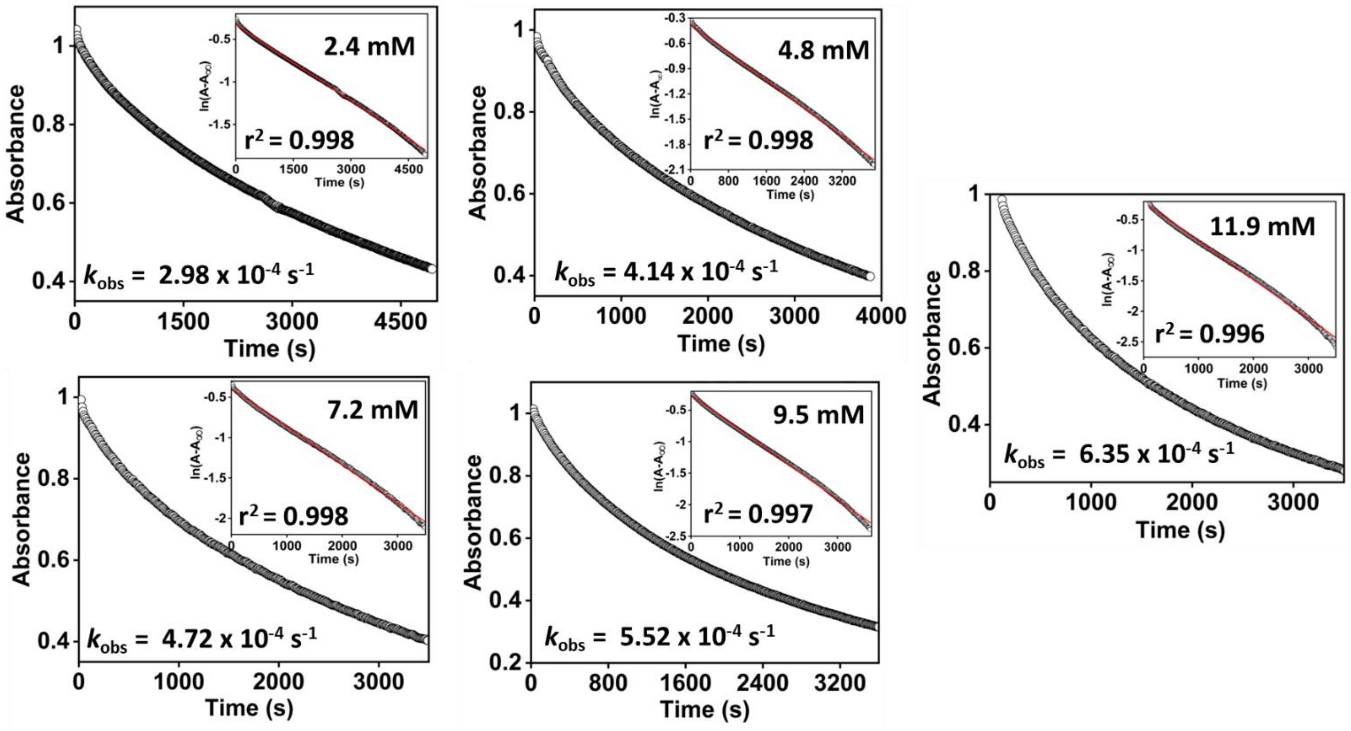

Figure S85. Decay of **3** (0.3 mM) at 465 nm upon addition of different concentrations (2.4–11.9 mM) of (4-Cl-C_6H_4)$_3$P at –10 °C (Inset: determination of k_{obs} value).

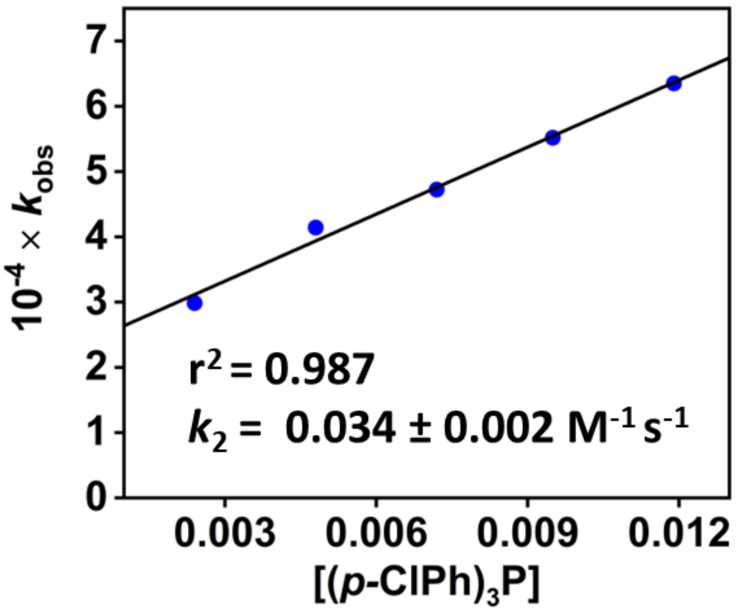

Figure S86. A plot of k_{obs} vs. [(4-Cl-C_6H_4)$_3$P]. The k_{obs} values were obtained by reacting **3** with different concentrations of (4-Cl-C_6H_4)$_3$P in acetonitrile at –10 °C. The second-order rate constant was obtained from the slope of the plot.

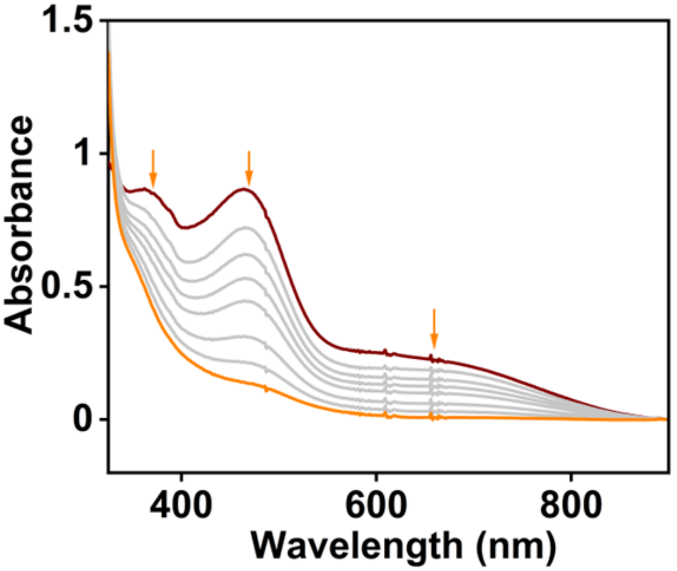

Figure S87. Change of single spectrum of **3** (0.22 mM) upon addition of 11.9 mM solution of (4-OMe-$C_6H_4)_3P$ in acetonitrile at –10 °C.

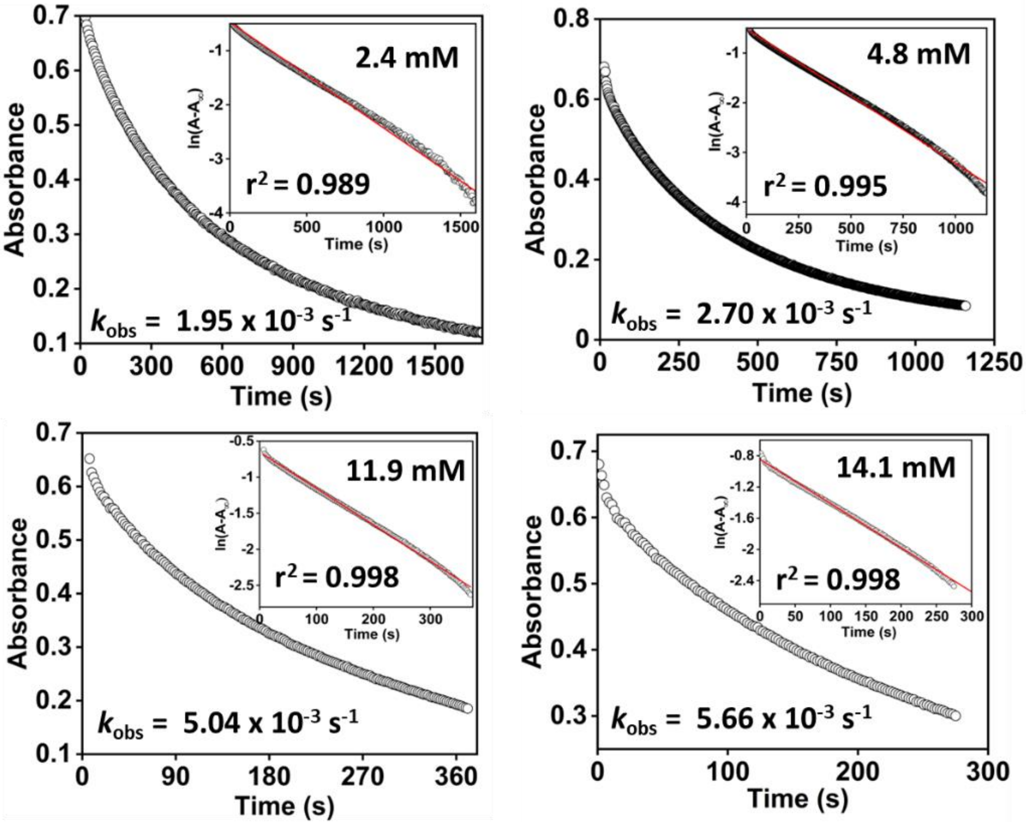

Figure S88. Decay of **3** (0.22 mM) at 465 nm upon addition of different concentrations (2.4–14.1 mM) of (4-OMe-$C_6H_4)_3P$ in acetonitrile at –10 °C (Inset: determination of k_{obs} value).

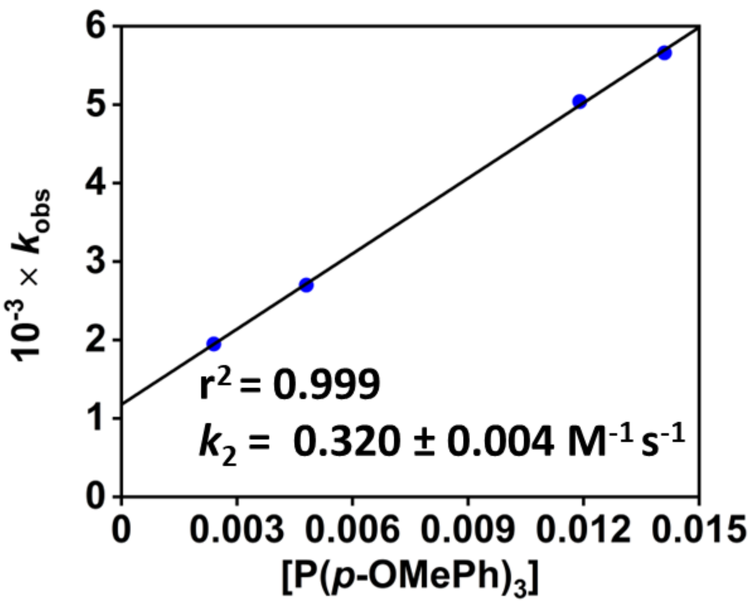

Figure S89. A plot of k_{obs} vs. (4-OMe-C_6H_4)$_3$P. The k_{obs} values were obtained by reacting **3** with different concentrations of (4-OMe-C_6H_4)$_3$P in acetonitrile at −10 °C. The second-order rate constant was obtained from the slope of the plot.

Table S11. The second-order rate constant values for the reaction of intermediate **3** with (4-X-C_6H_4)$_3$P (X= –OCH_3, –CH_3, –H, –Cl). σ_p^+ values are also mentioned in the plot.

**Phosphines**	σ_p	**E_{ox}/V *vs.* SCE[a]**	**k_2 (M^{-1} s^{-1})**	**log k_2**	**log k_{rel}**
(*p*-OMe-Ph)$_3$P	-0.27	0.8	0.320	-0.5	0.55948
(*p*-Me-Ph)$_3$P	-0.17	0.97	0.123	-0.91	0.1512
PPh_3	0	1.06	0.087	-1.06	0
(*p*-Cl-Ph)$_3$P	0.227	1.28	0.034	-1.46	-0.40563

[a]E_{ox} value of the Ar_3P substrates were taken from the reference [29]. The σ_p values were taken from the reference [28].

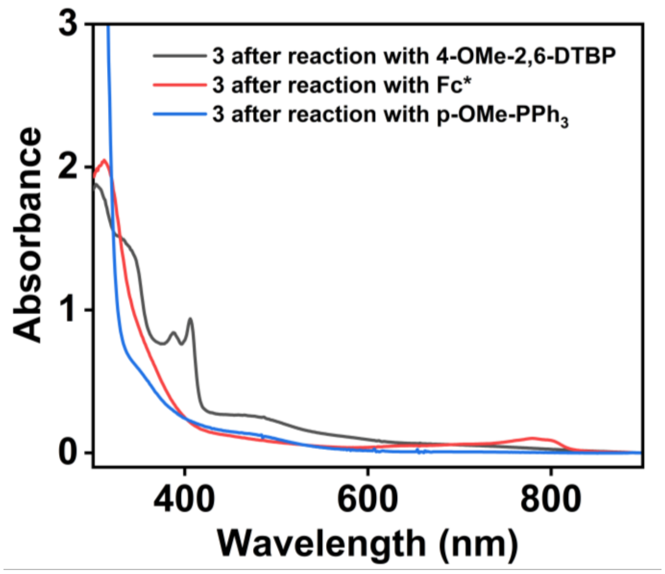

Figure S90. UV-vis spectra of the reaction solutions obtained upon reacting **3** (0.2 mM) with 4-OMe-DTBP, Fc*, and (4-OMe-$C_6H_4)_3P$ in acetonitrile at –25 °C.

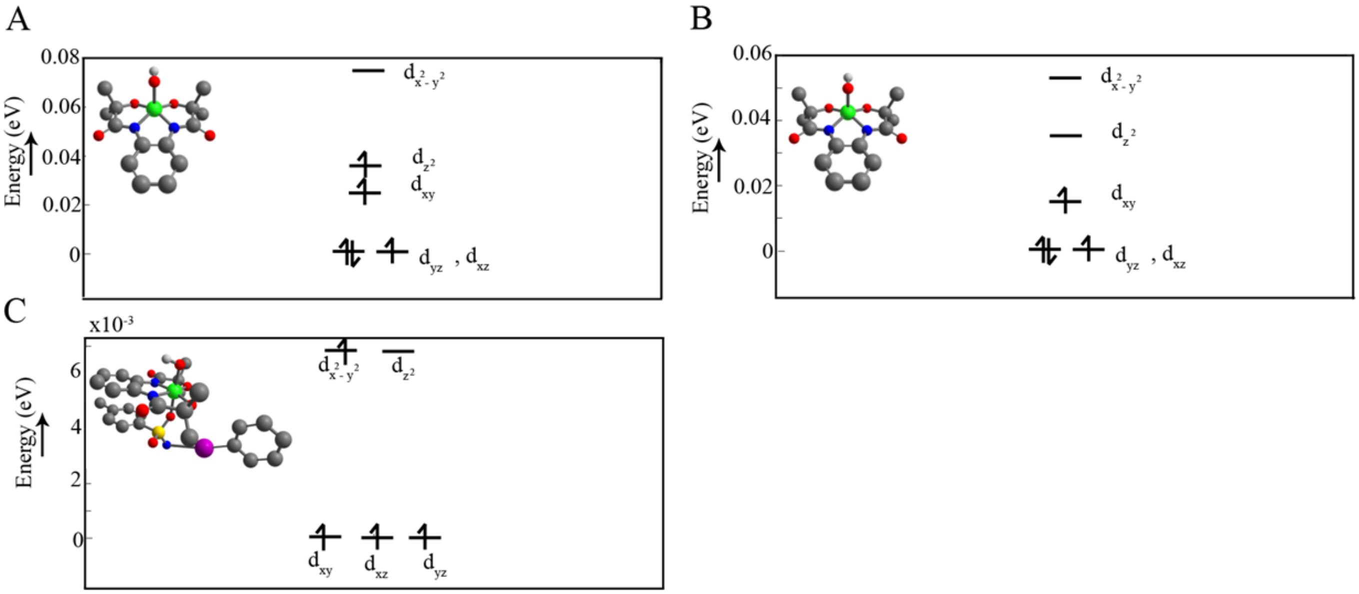

Figure S91. Simplified orbital scheme based on DFT calculations of A. Complexes **1**, B. **2**, and C. **3**

Table S12. Isosurface of the molecular orbitals assigned to the main UV-Vis transitions of Complexes **1**, **2**, and **3**.

Complexes	Isosurface of the molecular orbitals assigned to the main UV-Vis transitions
**1**	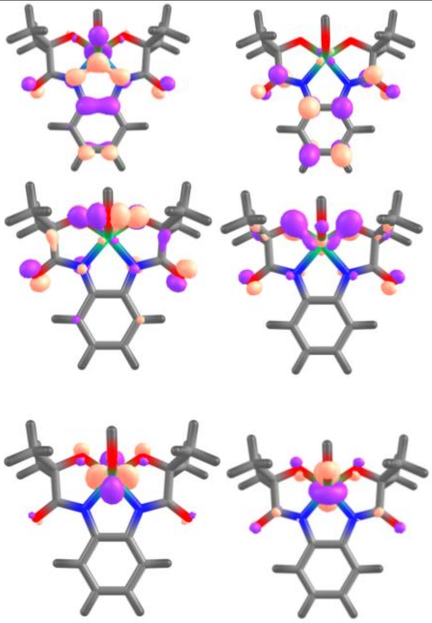
**2**	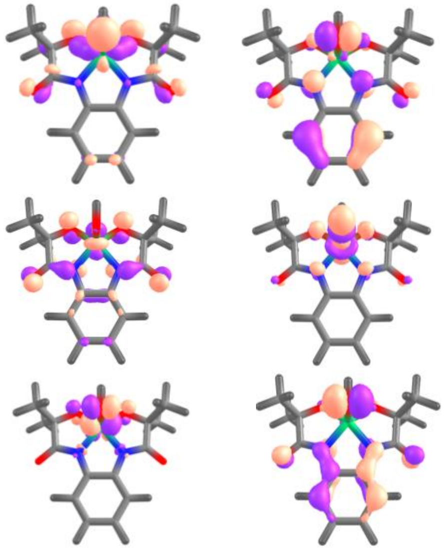
**3**	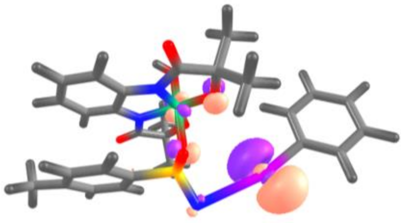

	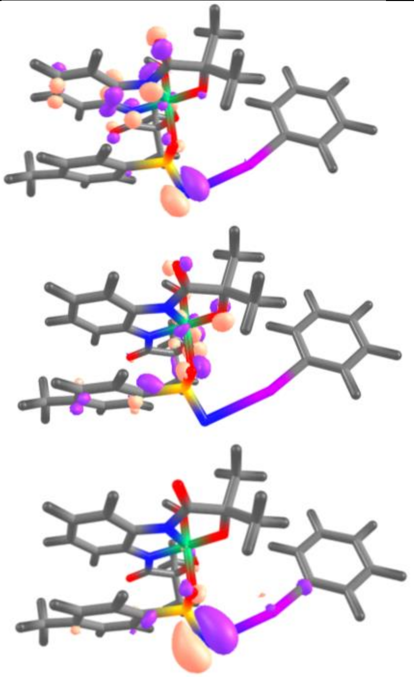

Table S13. LUMO, HOMO, LUMO- HUMO molecular orbitals of complexes **1**, **2** and **3** together with the LUMO-HUMO energy gaps. The LUMO-HUMO gaps were also determined for complexes **1**, **2** and **3**, Complexes **1, 2** and **3** display LUMO-HOMO energy gaps of 1.078 eV, -0.567 eV and 0.357 eV respectively with the energy gaps of **3** < **2** < **1**. The observed energy gap trends are in agreement with the lesser stable excited state[27] of complex **3** vs **1** and **2**. The LUMO-HOMO gaps of **2** and **3** are also very similar with the isosurface of the molecular orbitals distributed over the metal and ligand in comparison to **1**.

**Com -plex**	**LUMO**	**HOMO**	**LUMO-HOMO**
**1**	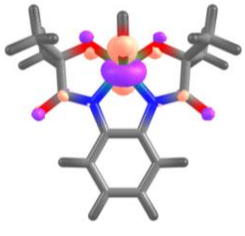	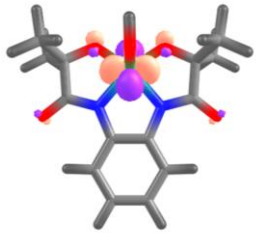	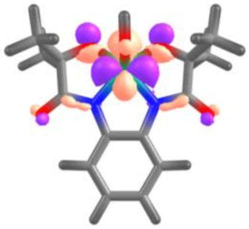 **1.07757936 eV**
**2**	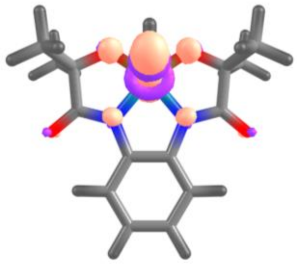	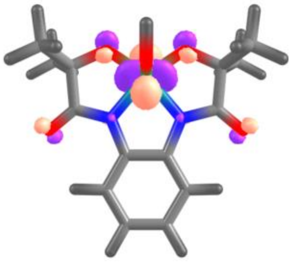	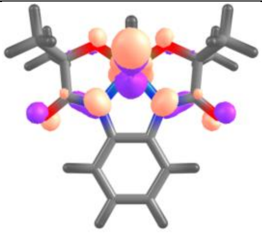 **0.56736186 eV**

**3**	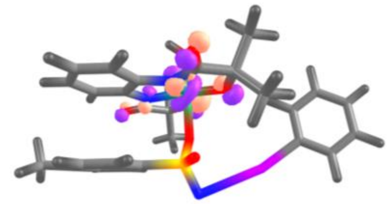	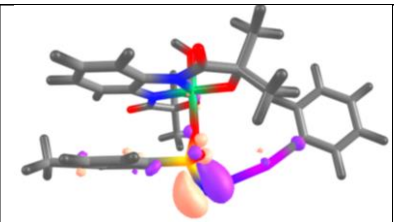	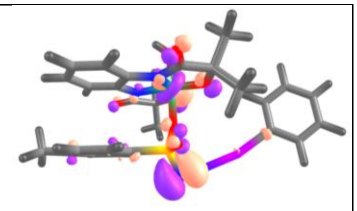 **0.357016192 eV**

Appendix

1. Fe^{III}(OH), Spin ½

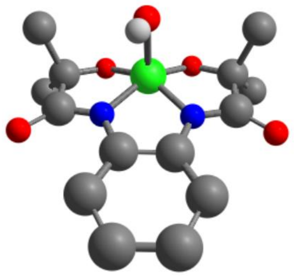

26	7.179966000	7.446168000	13.117294000
1	7.859841000	2.943136000	14.064012000
8	7.014798000	9.287580000	13.588546000
8	8.702271000	7.549675000	11.986634000
7	6.347717000	7.122226000	14.818688000
8	5.132510000	8.207955000	16.502354000
7	7.715082000	5.598794000	13.320961000
8	9.099707000	3.965632000	12.365221000
6	8.638017000	5.128396000	12.450059000
6	9.081926000	6.286261000	11.494191000
6	10.611433000	6.240181000	11.339840000
1	10.926365000	6.992290000	10.598352000
1	10.944231000	5.239003000	11.025891000
1	11.081331000	6.486108000	12.303950000
6	8.392064000	6.035426000	10.133255000
1	7.307388000	6.157469000	10.274177000
1	8.613231000	5.024400000	9.751047000
1	8.739598000	6.788150000	9.405417000
6	7.135448000	4.932683000	14.396181000
6	6.358886000	5.796747000	15.246646000
6	5.783332000	8.191688000	15.431223000
6	6.026170000	9.476879000	14.571158000
6	4.679353000	9.802852000	13.883024000

1	4.783067000	10.744839000	13.318569000
1	3.860929000	9.899914000	14.616543000
1	4.455352000	8.991213000	13.174298000
6	6.448710000	10.624461000	15.503769000
1	7.437289000	10.398791000	15.931385000
1	5.724977000	10.751744000	16.323558000
1	6.527682000	11.557244000	14.921969000
6	5.728081000	5.263733000	16.383593000
1	5.156095000	5.943225000	17.013749000
6	5.853580000	3.897463000	16.680278000
1	5.355198000	3.491457000	17.566370000
6	6.610278000	3.058046000	15.854909000
1	6.706596000	1.994306000	16.095164000
6	7.253505000	3.569012000	14.717289000
8	5.677434000	7.010594000	12.041646000
1	5.389191000	6.123792000	12.331032000

2. Fe^{III} (OH), Spin 3/2

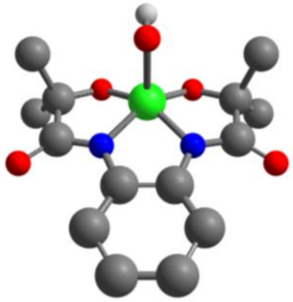

26	7.101674000	7.363160000	13.111634000
1	7.998687000	2.935242000	14.205550000
8	6.990725000	9.196009000	13.617199000
8	8.617715000	7.498253000	11.967084000
7	6.287426000	7.051564000	14.827177000
8	5.124449000	8.155564000	16.532158000
7	7.723464000	5.561860000	13.385592000
8	9.163847000	3.960289000	12.469537000
6	8.659324000	5.104198000	12.517235000
6	9.030227000	6.236188000	11.507791000
6	10.555408000	6.243757000	11.307976000
1	10.817167000	6.978500000	10.529347000
1	10.919997000	5.245440000	11.020471000
1	11.043340000	6.545504000	12.246965000
6	8.313578000	5.896975000	10.177897000
1	7.227802000	5.971185000	10.347511000
1	8.573806000	4.885279000	9.823028000
1	8.604384000	6.638843000	9.414431000

6	7.175578000	4.895268000	14.478335000
6	6.357872000	5.743391000	15.299059000
6	5.746593000	8.130007000	15.446975000
6	5.986089000	9.404500000	14.577181000
6	4.644408000	9.723761000	13.874225000
1	4.754912000	10.659835000	13.300406000
1	3.820345000	9.833845000	14.599592000
1	4.425223000	8.903753000	13.172237000
6	6.398187000	10.564144000	15.499705000
1	7.380524000	10.344339000	15.944209000
1	5.663540000	10.704035000	16.307764000
1	6.484964000	11.488801000	14.906261000
6	5.756145000	5.219356000	16.454748000
1	5.151298000	5.888366000	17.064742000
6	5.950457000	3.871470000	16.794527000
1	5.471709000	3.469123000	17.692980000
6	6.746990000	3.045155000	15.994747000
1	6.892952000	1.994674000	16.265839000
6	7.364136000	3.551565000	14.840341000
8	5.611852000	7.190292000	11.815037000
1	5.801838000	7.926044000	11.204752000

3. $Fe^{III}(OH)$ ligand oxidized complex, S = 1

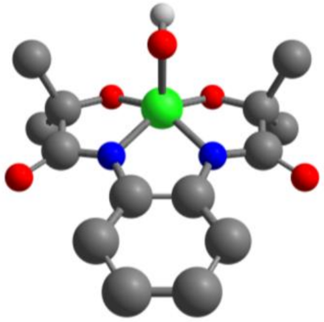

26	7.063714000	7.365428000	13.068536000
1	7.954328000	2.921805000	14.166314000
8	6.891031000	9.165164000	13.536136000
8	8.513432000	7.475640000	11.897747000
7	6.303091000	7.060112000	14.813982000
8	5.190923000	8.157751000	16.551722000
7	7.725964000	5.582343000	13.382834000
8	9.200679000	3.990341000	12.514970000
6	8.682668000	5.113258000	12.514802000
6	9.036088000	6.218128000	11.478031000
6	10.558017000	6.341718000	11.368166000
1	10.815823000	7.071167000	10.586841000

1	10.997686000	5.364448000	11.123974000
1	10.973246000	6.689842000	12.323848000
6	8.403309000	5.817134000	10.134156000
1	7.310155000	5.784694000	10.241770000
1	8.763209000	4.828006000	9.814049000
1	8.663847000	6.565209000	9.370396000
6	7.157270000	4.895822000	14.461695000
6	6.343348000	5.741286000	15.280395000
6	5.762188000	8.148608000	15.454911000
6	5.955961000	9.420911000	14.580510000
6	4.588773000	9.782698000	13.974235000
1	4.690767000	10.692354000	13.363639000
1	3.845330000	9.955984000	14.766472000
1	4.246160000	8.959482000	13.332066000
6	6.502126000	10.555314000	15.451577000
1	7.507536000	10.297069000	15.811281000
1	5.843414000	10.712047000	16.317198000
1	6.567336000	11.480384000	14.860776000
6	5.713267000	5.218802000	16.419089000
1	5.106196000	5.880503000	17.032491000
6	5.891543000	3.868904000	16.737404000
1	5.400273000	3.459588000	17.622827000
6	6.687830000	3.041534000	15.936050000
1	6.815556000	1.989078000	16.198477000
6	7.324843000	3.544705000	14.797445000
8	5.647330000	7.077790000	11.965993000
1	5.694376000	7.784482000	11.289433000

4. Fe^{IV}(OH)(PhINTs through O), S= 2

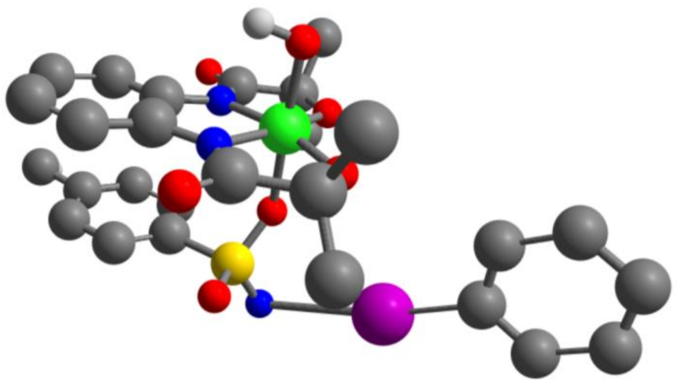

26	7.580115000	9.808713000	12.650615000
1	10.487075000	4.578197000	16.392261000
8	6.618470000	11.335030000	12.796962000
8	8.858267000	10.083095000	11.400483000

7	6.512303000	9.202529000	14.082612000
8	4.590514000	9.743768000	15.264315000
7	8.480224000	8.165673000	12.968855000
8	10.306932000	6.924202000	12.257788000
6	9.590097000	7.924003000	12.200788000
6	9.845972000	9.093652000	11.213932000
6	11.223736000	9.707369000	11.501082000
1	11.450376000	10.469509000	10.742903000
1	11.997015000	8.926744000	11.478868000
1	11.211060000	10.193026000	12.484739000
6	9.746036000	8.546489000	9.781773000
1	8.730368000	8.170910000	9.600620000
1	10.470541000	7.732975000	9.636828000
1	9.955628000	9.355914000	9.068774000
6	7.918466000	7.335560000	13.927857000
6	6.790479000	7.931810000	14.570888000
6	5.444642000	10.010730000	14.424580000
6	5.435278000	11.303258000	13.571185000
6	4.216945000	11.260365000	12.631839000
1	4.158289000	12.210085000	12.081826000
1	3.295879000	11.123222000	13.215642000
1	4.329057000	10.439530000	11.911522000
6	5.395390000	12.524727000	14.494492000
1	6.272703000	12.531623000	15.150979000
1	4.484139000	12.487097000	15.107793000
1	5.389676000	13.439244000	13.886526000
6	6.092755000	7.227205000	15.564333000
1	5.245749000	7.703027000	16.051221000
6	6.513601000	5.940855000	15.901128000
1	5.977755000	5.391066000	16.676693000
6	7.613656000	5.352272000	15.263000000
1	7.929253000	4.346079000	15.544329000
6	8.325145000	6.040112000	14.280293000
1	9.193325000	5.610777000	13.787360000
1	10.628385000	5.031195000	18.102167000
16	9.131952000	10.888759000	15.431079000
7	10.220972000	11.980150000	15.657482000
8	8.983610000	10.717636000	13.937715000
8	7.907085000	11.151296000	16.203868000
6	9.685690000	9.263705000	15.957432000
53	8.956973000	14.092705000	13.920013000
6	9.018215000	8.587444000	16.978398000
6	10.788002000	8.677019000	15.329163000
6	7.788418000	15.200374000	12.535495000

6	9.454503000	7.319163000	17.363405000
6	11.205402000	7.406922000	15.713064000
6	7.791880000	16.600012000	12.518710000
6	6.979318000	14.488002000	11.639309000
6	10.542954000	6.705200000	16.732582000
6	6.984905000	17.286531000	11.605930000
6	6.176391000	15.184296000	10.732042000
6	10.949852000	5.299635000	17.086105000
6	6.174461000	16.582656000	10.710562000
1	8.421348000	17.149934000	13.219326000
1	6.990142000	18.378745000	11.598440000
1	5.544913000	17.121053000	9.999841000
1	5.547612000	14.624050000	10.037008000
1	6.973625000	13.396550000	11.665631000
1	11.303305000	9.208210000	14.529572000
1	12.043502000	6.939386000	15.192116000
1	8.916064000	6.783068000	18.148122000
1	8.150404000	9.054087000	17.442677000
1	12.038996000	5.164200000	17.019667000
8	6.442399000	9.039527000	11.414180000
1	6.058600000	8.260646000	11.861118000

5. Fe^{IV}(OH)(PhINTs through O), S= 1

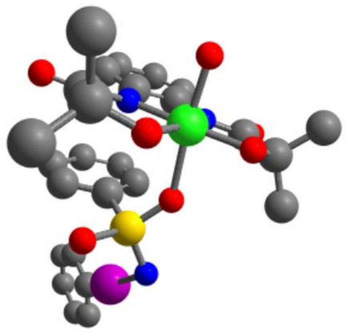

26	7.501025000	7.811906000	12.953768000
8	5.692309000	7.426646000	12.964557000
8	7.254166000	9.606242000	12.870864000
8	7.666292000	7.175831000	11.257252000
7	7.447628000	8.065531000	14.818275000
8	6.997898000	9.684496000	16.421367000
7	7.941856000	6.036746000	13.471873000
8	8.550694000	3.976000000	12.598211000
6	8.237355000	5.159707000	12.462229000
6	8.130957000	5.849755000	11.078563000

6	9.519277000	5.879566000	10.422338000
1	9.437676000	6.313535000	9.415807000
1	9.918082000	4.858305000	10.346614000
1	10.199782000	6.500253000	11.017129000
6	7.116947000	5.073562000	10.226126000
1	6.132380000	5.071959000	10.714193000
1	7.449680000	4.034456000	10.098399000
1	7.026629000	5.552064000	9.240951000
6	7.845396000	5.759461000	14.826141000
6	7.571053000	6.925837000	15.601552000
6	7.102034000	9.333521000	15.249904000
6	6.803269000	10.255397000	14.042389000
6	5.275303000	10.453746000	13.982754000
1	5.037822000	11.117574000	13.139370000
1	4.920723000	10.913564000	14.916108000
1	4.779632000	9.486596000	13.832234000
6	7.537567000	11.588876000	14.196585000
1	8.621561000	11.428869000	14.156703000
1	7.264028000	12.044687000	15.158520000
1	7.244417000	12.261867000	13.378706000
6	7.445676000	6.831846000	16.995194000
1	7.256238000	7.733776000	17.570215000
6	7.577929000	5.583372000	17.601531000
1	7.488468000	5.506089000	18.686273000
6	7.836787000	4.436206000	16.839085000
1	7.941993000	3.468612000	17.333103000
6	7.974325000	4.511560000	15.452941000
1	8.192401000	3.636745000	14.845958000
1	5.491684000	7.430962000	12.007788000
8	9.594077000	8.166638000	12.758970000
16	10.673239000	8.859096000	13.552705000
7	12.034775000	8.591892000	12.834580000
53	14.105973000	7.991034000	14.578556000
6	15.541763000	7.498307000	16.083698000
6	15.079428000	7.118388000	17.351202000
6	16.918411000	7.543489000	15.832500000
6	15.991231000	6.785809000	18.356516000
6	17.825609000	7.209719000	16.842841000
6	17.366613000	6.829879000	18.107444000
1	14.005416000	7.084356000	17.543484000
1	17.279881000	7.839630000	14.846651000
1	15.622468000	6.490506000	19.341170000
1	18.897923000	7.247536000	16.638496000
1	18.076702000	6.569766000	18.894336000

6	10.637020000	8.064786000	15.160360000
6	10.815465000	6.678987000	15.232470000
6	10.571681000	8.837302000	16.319776000
6	10.936898000	6.066805000	16.477138000
6	10.689594000	8.215417000	17.564758000
6	10.876081000	6.833762000	17.644632000
1	10.873602000	6.091508000	14.316433000
1	10.434084000	9.913798000	16.231991000
1	11.066337000	4.986167000	16.535802000
1	10.627840000	8.814311000	18.474781000
1	10.958967000	6.348527000	18.618524000
8	10.490001000	10.296294000	13.804025000

6. Fe^{IV}(OH)(PhINTs through N), S = 2

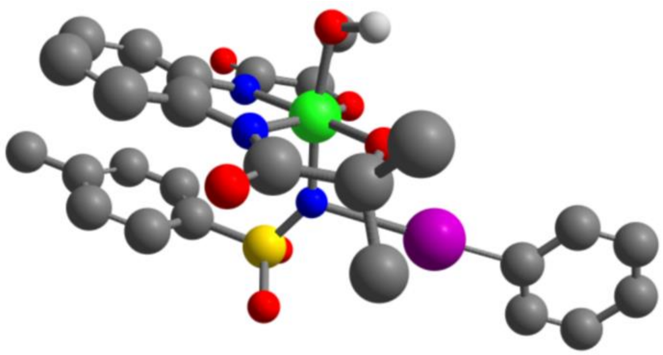

26	7.094729000	8.496760000	12.518176000
1	11.113178000	7.596125000	19.176031000
8	5.513738000	9.312776000	12.054006000
8	8.323417000	8.683325000	11.160712000
7	6.211439000	8.213376000	14.182080000
8	4.277326000	8.725983000	15.351728000
7	8.446100000	7.477930000	13.356846000
8	10.578900000	6.626526000	13.024559000
6	9.612048000	7.293092000	12.653531000
6	9.561300000	8.025935000	11.293363000
6	10.697718000	9.053706000	11.213988000
1	10.701540000	9.514137000	10.216582000
1	11.660788000	8.553969000	11.390849000
1	10.550107000	9.848192000	11.956914000
6	9.665252000	6.967388000	10.180293000
1	8.806018000	6.286795000	10.239880000
1	10.597962000	6.397656000	10.294737000
1	9.657880000	7.470483000	9.203611000

6	8.077661000	6.845859000	14.540668000
6	6.808693000	7.278570000	15.020141000
6	4.986428000	8.802633000	14.346388000
6	4.569325000	9.577805000	13.072528000
6	3.199130000	9.055038000	12.615229000
1	2.888114000	9.597896000	11.712020000
1	2.455817000	9.206111000	13.409680000
1	3.257611000	7.982098000	12.384801000
6	4.518293000	11.081256000	13.378493000
1	5.504704000	11.446828000	13.687652000
1	3.794628000	11.270799000	14.184075000
1	4.210894000	11.624036000	12.474063000
6	6.281330000	6.735073000	16.199143000
1	5.318026000	7.088214000	16.558003000
6	7.007009000	5.752083000	16.876860000
1	6.592947000	5.319709000	17.789766000
6	8.247779000	5.316770000	16.398876000
1	8.800838000	4.546208000	16.938400000
6	8.796901000	5.865580000	15.238174000
1	9.764699000	5.553809000	14.855177000
1	11.082943000	6.289469000	17.975000000
16	8.592011000	11.007131000	14.149108000
7	7.750418000	10.285572000	13.000728000
8	7.676250000	11.939435000	14.820500000
8	9.813776000	11.553780000	13.546964000
6	9.145156000	9.864881000	15.404254000
53	7.394585000	12.056662000	10.712619000
6	10.387600000	9.240654000	15.268833000
6	8.337051000	9.603763000	16.512125000
6	7.098863000	13.314208000	9.028290000
6	10.812300000	8.344123000	16.244546000
6	8.782630000	8.715586000	17.489136000
6	6.473443000	12.812921000	7.878808000
6	7.527692000	14.647927000	9.055956000
6	10.019632000	8.069146000	17.368823000
6	6.279985000	13.640516000	6.769399000
6	7.330811000	15.469929000	7.942969000
6	10.484668000	7.097038000	18.419823000
6	6.706961000	14.971435000	6.795503000
1	6.139734000	11.774836000	7.856017000
1	5.791572000	13.239329000	5.878783000
1	6.555006000	15.615385000	5.927658000
1	7.669329000	16.507747000	7.975276000
1	8.015611000	15.039108000	9.949426000

1	7.368515000	10.095110000	16.597159000
1	8.142227000	8.494738000	18.344881000
1	11.767773000	7.830724000	16.119519000
1	11.006989000	9.456464000	14.399835000
1	9.630770000	6.642826000	18.940451000
8	6.441882000	6.888556000	11.805339000

7. Fe^{IV}(O), S = 1

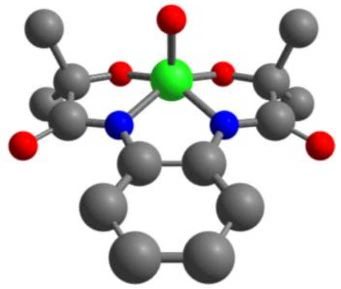

26	7.061281000	7.493028000	12.893579000
8	5.788696000	7.243319000	11.861614000
8	6.992577000	9.333675000	13.425926000
8	8.631120000	7.620747000	11.804926000
7	6.220913000	7.193507000	14.605716000
8	5.056748000	8.304247000	16.305095000
7	7.669581000	5.684282000	13.181125000
8	9.115946000	4.067915000	12.302192000
6	8.624386000	5.217600000	12.338480000
6	9.048574000	6.355941000	11.355348000
6	10.579629000	6.350721000	11.221341000
1	10.881860000	7.086608000	10.458939000
1	10.945056000	5.349790000	10.944550000
1	11.029864000	6.641901000	12.182226000
6	8.378864000	6.041848000	9.996887000
1	7.290972000	6.145274000	10.125852000
1	8.624925000	5.024539000	9.647949000
1	8.716465000	6.779985000	9.250215000
6	7.056393000	5.015696000	14.237437000
6	6.234084000	5.872985000	15.046762000
6	5.692050000	8.276371000	15.227842000
6	5.980115000	9.554505000	14.375958000
6	4.660959000	9.911499000	13.651762000
1	4.800308000	10.853097000	13.094717000
1	3.823021000	10.021701000	14.360916000

1	4.445557000	9.106893000	12.932508000
6	6.410718000	10.691386000	15.315993000
1	7.382683000	10.442095000	15.767674000
1	5.670778000	10.836968000	16.117951000
1	6.525574000	11.621102000	14.735786000
6	5.572328000	5.343589000	16.166685000
1	4.968183000	6.019256000	16.769958000
6	5.712129000	3.983733000	16.483514000
1	5.189291000	3.578346000	17.355531000
6	6.512031000	3.149183000	15.695413000
1	6.616469000	2.089727000	15.949860000
6	7.187334000	3.659048000	14.575962000
1	7.828117000	3.036532000	13.953638000

8. Fe^{IV}(OH), S = 1

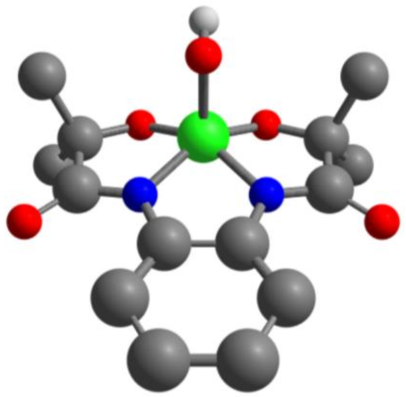

26	7.052920000	7.479979000	12.906006000
8	5.648981000	7.215876000	11.782355000
8	6.889393000	9.279717000	13.376484000
8	8.520054000	7.580242000	11.757767000
7	6.268969000	7.175372000	14.641201000
8	5.149585000	8.278093000	16.371208000
7	7.692734000	5.688215000	13.221128000
8	9.167882000	4.087403000	12.370198000
6	8.658444000	5.214098000	12.365717000
6	9.034075000	6.319346000	11.337475000
6	10.558140000	6.427848000	11.242449000
1	10.830589000	7.157447000	10.466303000
1	10.989800000	5.446942000	10.998740000
1	10.968020000	6.768064000	12.203257000
6	8.410512000	5.930531000	9.985464000
1	7.316101000	5.909713000	10.082298000

1	8.763435000	4.939110000	9.664853000
1	8.686724000	6.679100000	9.227728000
6	7.105753000	5.004049000	14.291504000
6	6.290987000	5.854830000	15.103856000
6	5.731324000	8.266846000	15.280041000
6	5.944532000	9.539629000	14.411137000
6	4.586737000	9.913475000	13.790962000
1	4.702582000	10.823662000	13.183692000
1	3.836347000	10.090586000	14.575701000
1	4.244845000	9.094427000	13.143125000
6	6.489389000	10.668066000	15.290834000
1	7.488962000	10.401907000	15.660828000
1	5.822184000	10.827404000	16.149414000
1	6.567552000	11.593902000	14.702876000
6	5.643178000	5.334974000	16.233785000
1	5.035804000	6.000792000	16.842381000
6	5.804861000	3.982485000	16.549815000
1	5.299835000	3.575276000	17.428412000
6	6.602225000	3.149955000	15.754899000
1	6.717060000	2.095610000	16.015582000
6	7.256736000	3.650388000	14.625106000
1	7.887577000	3.023542000	13.999266000
1	5.709908000	7.928225000	11.113065000

9. $Fe^{IV}(OH)(OH_2)$, S = 1

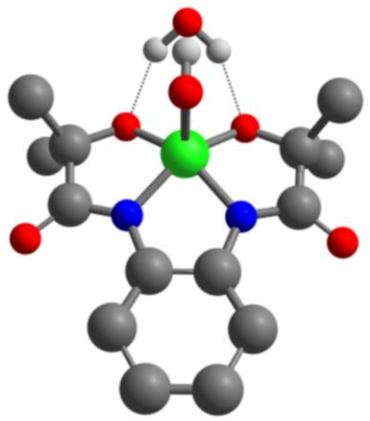

26	7.259472000	7.305295000	13.240912000
8	6.068822000	7.472023000	11.904993000
8	7.269022000	9.080735000	13.927004000
8	8.893677000	7.385736000	12.275825000
7	6.267903000	6.922413000	14.829651000
8	5.119167000	7.949032000	16.584831000

7	7.683400000	5.448420000	13.395885000
8	9.107591000	3.800919000	12.552123000
6	8.711641000	4.968445000	12.611558000
6	9.327129000	6.123748000	11.770647000
6	10.853034000	6.064474000	11.874871000
1	11.299245000	6.828774000	11.222632000
1	11.212552000	5.068849000	11.579681000
1	11.163294000	6.262314000	12.909965000
6	8.850525000	5.948921000	10.318912000
1	7.757612000	6.053428000	10.276298000
1	9.136063000	4.958703000	9.934620000
1	9.304081000	6.727665000	9.687742000
6	6.922705000	4.719904000	14.321557000
6	6.111484000	5.564909000	15.143473000
6	5.799652000	7.997277000	15.556138000
6	6.241651000	9.329506000	14.885430000
6	5.008637000	9.932118000	14.190621000
1	5.274840000	10.902825000	13.746091000
1	4.190756000	10.076614000	14.911647000
1	4.671217000	9.259122000	13.390215000
6	6.800961000	10.272375000	15.953750000
1	7.714998000	9.844913000	16.388065000
1	6.060780000	10.416149000	16.753253000
1	7.048158000	11.242258000	15.498977000
6	5.301841000	5.005983000	16.142114000
1	4.699568000	5.666577000	16.760998000
6	5.300883000	3.619135000	16.318227000
1	4.669783000	3.180032000	17.093706000
6	6.094765000	2.792153000	15.514002000
1	6.080389000	1.710768000	15.665181000
6	6.909247000	3.331365000	14.513742000
1	7.539366000	2.706921000	13.885118000
8	7.765590000	9.775593000	11.229937000
1	8.395973000	9.020472000	11.314070000
1	7.535088000	9.901222000	12.182250000
1	6.376828000	8.268673000	11.396546000

10. $Fe^{IV}(OH)(CH_3CN)$, S = 1

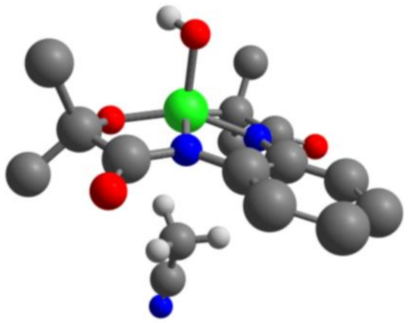

26	7.000342000	7.253536000	12.933983000
8	5.525124000	6.854596000	11.986071000
8	6.847034000	9.056101000	13.255891000
8	8.306718000	7.426875000	11.653860000
7	6.423800000	7.048333000	14.718226000
8	5.454435000	8.243391000	16.469883000
7	7.770653000	5.564871000	13.266503000
8	9.277109000	4.037254000	12.352612000
6	8.677813000	5.118211000	12.340664000
6	8.853435000	6.187478000	11.231067000
6	10.345532000	6.384432000	10.944699000
1	10.473026000	7.083775000	10.106275000
1	10.812685000	5.420863000	10.699121000
1	10.847944000	6.805356000	11.826575000
6	8.108153000	5.688238000	9.979903000
1	7.038169000	5.583999000	10.207062000
1	8.503548000	4.714524000	9.655385000
1	8.230537000	6.420903000	9.168358000
6	7.356231000	4.921311000	14.432117000
6	6.577467000	5.776409000	15.268811000
6	5.915660000	8.167563000	15.325902000
6	5.997337000	9.369511000	14.349542000
6	4.573907000	9.654320000	13.838198000
1	4.599976000	10.513837000	13.152052000
1	3.898552000	9.877011000	14.677173000
1	4.195597000	8.776732000	13.295812000
6	6.577549000	10.582876000	15.082638000
1	7.620093000	10.388575000	15.369673000
1	5.995133000	10.790875000	15.990763000
1	6.558182000	11.459780000	14.420046000

6	6.097202000	5.308716000	16.500554000
1	5.515038000	5.980113000	17.127617000
6	6.391036000	3.998245000	16.889265000
1	6.018031000	3.629100000	17.846753000
6	7.155187000	3.159375000	16.068216000
1	7.375602000	2.139080000	16.388590000
6	7.644113000	3.610709000	14.838573000
1	8.248501000	2.979592000	14.191100000
1	5.514618000	7.534882000	11.283316000
7	11.761598000	9.079313000	16.064423000
6	10.874249000	8.519561000	15.561190000
6	9.767692000	7.820240000	14.931102000
1	9.008270000	7.547513000	15.676289000
1	10.118457000	6.897507000	14.450491000
1	9.288759000	8.447798000	14.164784000

11. Fe^{III}(OMe), S = ½

26	7.160346000	7.407571000	13.069284000
1	7.996512000	2.970992000	14.165422000
8	6.987109000	9.242308000	13.553651000
8	8.627822000	7.506357000	11.833064000
7	6.272401000	7.092180000	14.761100000
8	5.117763000	8.195405000	16.479293000
7	7.766169000	5.600075000	13.350651000
8	9.221258000	3.980178000	12.481659000
6	8.712343000	5.125141000	12.504354000
6	9.101345000	6.233029000	11.470022000
6	10.634747000	6.281590000	11.353126000
1	10.918358000	6.986589000	10.554786000
1	11.043715000	5.282543000	11.137169000
1	11.057754000	6.644804000	12.301611000
6	8.476489000	5.799581000	10.122786000
1	7.382839000	5.788310000	10.232656000
1	8.821458000	4.795549000	9.824670000
1	8.747770000	6.531523000	9.344016000
6	7.173625000	4.932482000	14.423253000
6	6.328883000	5.778919000	15.221458000
6	5.741083000	8.164914000	15.391978000
6	5.998504000	9.449421000	14.536785000
6	4.651792000	9.802766000	13.865730000
1	4.776147000	10.714368000	13.258096000
1	3.860618000	9.963425000	14.617015000
1	4.363879000	8.971374000	13.206554000

6	6.447572000	10.587087000	15.468094000
1	7.438904000	10.347142000	15.880497000
1	5.736256000	10.714744000	16.298306000
1	6.528507000	11.523588000	14.892574000
6	5.684486000	5.246554000	16.351060000
1	5.063645000	5.914752000	16.945872000
6	5.860963000	3.895878000	16.686938000
1	5.350948000	3.489195000	17.566179000
6	6.681381000	3.071784000	15.908806000
1	6.814282000	2.018840000	16.177214000
6	7.341174000	3.584749000	14.781341000
8	5.642159000	7.063630000	11.941782000
6	5.540410000	7.907442000	10.835918000
1	4.781145000	7.504347000	10.122652000
1	5.221346000	8.941775000	11.104155000
1	6.496898000	8.013284000	10.280495000

12. Fe^{III}(OMe), S = 3/2

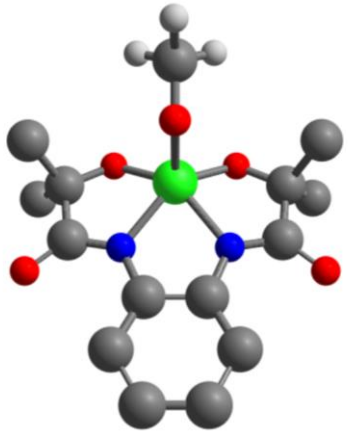

26	7.114544000	7.354687000	13.065830000
1	8.009435000	2.936837000	14.212318000
8	6.973852000	9.189860000	13.548452000
8	8.632547000	7.475370000	11.926816000
7	6.277052000	7.053218000	14.774739000
8	5.140671000	8.169362000	16.490006000
7	7.734952000	5.555153000	13.363753000
8	9.222731000	3.958458000	12.517361000
6	8.700862000	5.095242000	12.529588000
6	9.080976000	6.211019000	11.508093000
6	10.610427000	6.244124000	11.349499000
1	10.879032000	6.965625000	10.561126000
1	11.002077000	5.246907000	11.096143000
1	11.066085000	6.577468000	12.293872000
6	8.409933000	5.824372000	10.168742000

1	7.319096000	5.849341000	10.308776000
1	8.718791000	4.818733000	9.837334000
1	8.684738000	6.565836000	9.400433000
6	7.175423000	4.896222000	14.456360000
6	6.346131000	5.748753000	15.259422000
6	5.749525000	8.136788000	15.397749000
6	5.980998000	9.404795000	14.519302000
6	4.627712000	9.716203000	13.836711000
1	4.733278000	10.630655000	13.230094000
1	3.823452000	9.856019000	14.578393000
1	4.376661000	8.877883000	13.170057000
6	6.402859000	10.572397000	15.426743000
1	7.393406000	10.359424000	15.855721000
1	5.681272000	10.713468000	16.246104000
1	6.475537000	11.493469000	14.826205000
6	5.732150000	5.233703000	16.412588000
1	5.120485000	5.906981000	17.010754000
6	5.925921000	3.889536000	16.766429000
1	5.438067000	3.493982000	17.662910000
6	6.734207000	3.058560000	15.983596000
1	6.880334000	2.011159000	16.265972000
6	7.363840000	3.556441000	14.832391000
8	5.650557000	7.177127000	11.741154000
6	5.608335000	8.097978000	10.708556000
1	4.791068000	7.857543000	9.979645000
1	5.424615000	9.142401000	11.059149000
1	6.557169000	8.143090000	10.123184000

13. Fe^{III}(OMe), S = 5/2

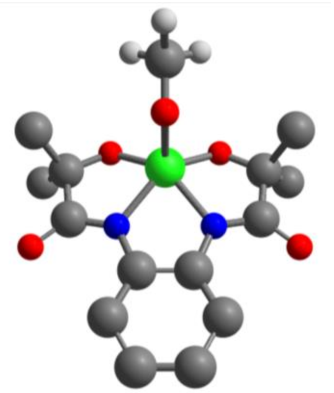

26	7.086626000	7.498347000	12.881116000
1	8.035808000	2.969811000	14.215753000
8	6.845218000	9.298962000	13.551498000
8	8.669485000	7.411126000	11.771091000

7	6.279801000	7.086714000	14.781786000
8	5.128455000	8.163930000	16.519824000
7	7.762489000	5.560652000	13.346772000
8	9.218654000	3.917981000	12.517300000
6	8.703828000	5.061779000	12.522633000
6	9.131276000	6.131683000	11.454584000
6	10.668548000	6.143887000	11.366209000
1	10.983268000	6.819705000	10.554085000
1	11.060386000	5.131499000	11.186661000
1	11.081771000	6.523629000	12.312622000
6	8.519957000	5.677390000	10.107422000
1	7.424115000	5.724707000	10.189239000
1	8.826089000	4.649051000	9.856062000
1	8.842507000	6.366170000	9.309044000
6	7.207242000	4.931078000	14.447377000
6	6.374963000	5.789507000	15.254813000
6	5.731414000	8.138471000	15.420282000
6	5.912020000	9.452854000	14.579103000
6	4.524577000	9.780607000	13.976385000
1	4.581442000	10.733142000	13.423692000
1	3.753656000	9.853501000	14.760548000
1	4.256160000	8.980905000	13.270496000
6	6.364397000	10.580951000	15.523974000
1	7.375838000	10.357495000	15.895010000
1	5.682226000	10.672688000	16.382401000
1	6.400970000	11.531907000	14.967537000
6	5.770199000	5.266745000	16.413004000
1	5.158187000	5.940613000	17.010798000
6	5.968779000	3.927208000	16.776728000
1	5.488182000	3.537135000	17.680071000
6	6.775182000	3.094841000	15.993631000
1	6.929485000	2.049332000	16.280424000
6	7.393240000	3.591795000	14.837229000
8	5.629206000	7.263519000	11.628152000
6	5.532252000	8.161208000	10.575552000
1	4.664935000	7.914028000	9.914694000
1	5.387401000	9.212942000	10.918184000
1	6.440570000	8.166688000	9.928288000

14. Fe^{IV}(OMe)(PhINTs, bound through O atom), S = 2

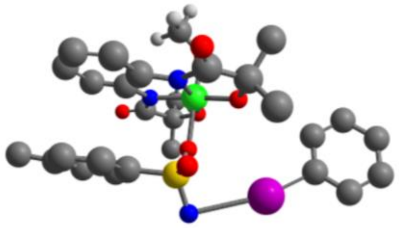

26	7.449705000	9.748105000	12.652691000
1	10.508272000	4.556455000	16.522942000
8	6.528141000	11.299626000	12.829327000
8	8.706595000	9.996962000	11.364771000
7	6.434815000	9.179035000	14.135408000
8	4.559613000	9.754323000	15.373587000
7	8.337407000	8.099204000	12.954541000
8	10.168238000	6.855759000	12.252481000
6	9.449222000	7.852878000	12.186395000
6	9.697836000	9.013595000	11.187950000
6	11.076941000	9.631140000	11.463387000
1	11.300330000	10.381360000	10.692324000
1	11.851794000	8.851755000	11.451366000
1	11.064773000	10.133314000	12.438331000
6	9.600960000	8.449182000	9.761320000
1	8.588396000	8.063457000	9.582239000
1	10.329934000	7.638399000	9.623441000
1	9.803060000	9.251609000	9.038504000
6	7.834192000	7.308576000	13.981295000
6	6.740997000	7.928749000	14.657463000
6	5.389650000	10.002495000	14.503613000
6	5.370608000	11.289268000	13.641672000
6	4.117355000	11.262524000	12.748432000
1	4.055829000	12.208174000	12.191689000
1	3.216848000	11.148125000	13.368033000
1	4.183806000	10.434101000	12.031887000
6	5.380607000	12.515086000	14.560256000
1	6.287824000	12.518904000	15.174954000
1	4.498895000	12.485789000	15.215801000
1	5.352916000	13.428322000	13.950513000
6	6.096179000	7.263453000	15.710886000
1	5.272206000	7.756389000	16.219685000
6	6.537756000	5.991786000	16.076338000
1	6.044020000	5.472556000	16.899602000

6	7.607369000	5.380905000	15.408883000
1	7.942215000	4.388309000	15.714940000
6	8.264691000	6.030399000	14.363867000
1	9.108230000	5.583037000	13.845034000
1	10.844682000	5.096129000	18.178990000
16	9.127253000	10.836589000	15.380751000
7	10.222314000	11.933733000	15.542384000
8	8.919548000	10.630698000	13.900095000
8	7.933097000	11.113470000	16.195635000
6	9.710544000	9.226790000	15.924284000
53	8.934600000	14.015772000	13.826015000
6	9.086288000	8.573777000	16.987059000
6	10.794551000	8.631719000	15.273176000
6	7.743405000	15.128475000	12.463547000
6	9.548026000	7.321145000	17.391699000
6	11.236877000	7.375879000	15.677219000
6	7.784616000	16.526912000	12.416570000
6	6.882404000	14.421384000	11.612744000
6	10.619348000	6.698315000	16.739781000
6	6.963890000	17.217646000	11.519317000
6	6.066241000	15.121657000	10.720387000
6	11.052021000	5.307395000	17.120368000
6	6.102193000	16.518790000	10.668948000
1	8.454707000	17.073004000	13.081735000
1	6.999005000	18.308950000	11.488288000
1	5.461833000	17.060232000	9.970222000
1	5.397228000	14.565427000	10.060441000
1	6.847892000	13.331059000	11.663158000
1	11.278061000	9.144737000	14.442412000
1	12.061441000	6.901470000	15.140997000
1	9.043410000	6.803573000	18.210609000
1	8.231770000	9.046667000	17.469238000
1	12.125550000	5.154474000	16.940039000
8	6.239456000	9.072444000	11.430594000
6	5.698164000	7.790392000	11.574875000
1	5.096307000	7.671003000	12.496166000
1	5.029961000	7.631159000	10.708548000
1	6.465521000	6.993219000	11.568011000

15. Fe^{IV}(OMe)(PhINTs bound through N atom), S = 2

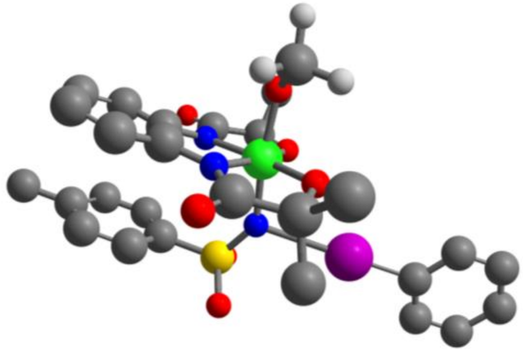

26	7.087704000	8.567312000	12.344401000
1	10.855721000	7.836836000	19.225539000
8	5.481599000	9.366647000	11.905598000
8	8.305831000	8.722031000	10.973128000
7	6.200546000	8.227892000	13.986039000
8	4.308932000	8.774128000	15.214366000
7	8.499401000	7.609712000	13.214091000
8	10.645682000	6.800860000	12.883820000
6	9.653521000	7.431697000	12.506659000
6	9.563940000	8.098831000	11.113701000
6	10.675978000	9.140434000	10.946607000
1	10.638907000	9.553704000	9.929240000
1	11.651053000	8.660090000	11.111294000
1	10.545100000	9.961312000	11.662096000
6	9.663636000	6.986461000	10.052833000
1	8.823562000	6.289665000	10.167942000
1	10.612821000	6.446224000	10.173485000
1	9.620009000	7.438085000	9.052182000
6	8.130544000	6.956804000	14.386764000
6	6.830382000	7.318184000	14.836910000
6	4.995242000	8.853568000	14.194072000
6	4.600442000	9.698051000	12.958600000
6	3.162604000	9.349072000	12.545123000
1	2.887057000	9.942056000	11.662171000
1	2.477673000	9.583199000	13.371070000
1	3.069986000	8.283805000	12.300666000
6	4.691964000	11.191227000	13.318221000
1	5.707976000	11.467894000	13.618706000
1	4.000467000	11.411826000	14.143850000
1	4.415733000	11.788803000	12.438832000
6	6.297677000	6.741327000	15.996669000

1	5.310055000	7.047178000	16.332016000
6	7.051670000	5.790557000	16.690915000
1	6.635950000	5.332325000	17.590183000
6	8.323336000	5.423071000	16.241570000
1	8.901879000	4.678389000	16.791087000
6	8.876617000	6.008545000	15.099288000
1	9.870404000	5.751130000	14.742965000
1	10.859248000	6.474872000	18.087257000
16	8.654862000	11.048697000	13.930693000
7	7.749738000	10.336612000	12.819916000
8	7.813463000	12.081565000	14.548661000
8	9.908085000	11.472858000	13.296710000
6	9.122397000	9.947399000	15.253264000
53	7.375631000	12.121399000	10.534308000
6	10.375081000	9.330408000	15.232208000
6	8.234965000	9.720708000	16.306786000
6	7.044276000	13.400568000	8.874285000
6	10.733093000	8.479789000	16.273991000
6	8.611411000	8.873031000	17.346504000
6	6.391868000	12.914560000	7.733318000
6	7.471270000	14.734519000	8.911737000
6	9.860483000	8.239276000	17.345840000
6	6.169644000	13.758276000	6.641648000
6	7.245461000	15.572448000	7.816276000
6	10.255043000	7.311993000	18.464231000
6	6.594416000	15.089714000	6.677249000
1	6.059244000	11.876344000	7.703490000
1	5.660026000	13.369349000	5.757524000
1	6.419529000	15.746300000	5.823322000
1	7.582332000	16.610535000	7.855879000
1	7.979518000	15.113533000	9.799058000
1	7.258480000	10.203942000	16.303370000
1	7.908984000	8.674340000	18.157687000
1	11.699541000	7.972466000	16.241692000
1	11.049927000	9.513778000	14.397882000
1	9.369011000	6.896184000	18.962566000
8	6.542748000	6.935881000	11.582631000
6	5.217881000	6.521206000	11.651133000
1	4.842376000	6.445315000	12.692569000
1	4.546364000	7.212216000	11.104062000
1	5.145521000	5.518308000	11.189867000

16. Fe^{III} (OMe) ligand oxidized complex, S = 2

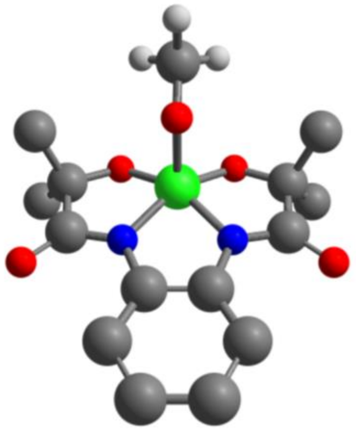

26	7.167689000	7.398544000	13.064542000
1	8.005772000	2.944397000	14.197603000
8	6.892994000	9.160831000	13.506765000
8	8.591030000	7.439767000	11.901719000
7	6.286972000	7.063161000	14.744257000
8	5.085247000	8.138091000	16.425061000
7	7.734895000	5.581768000	13.355047000
8	9.166054000	3.956201000	12.497781000
6	8.688776000	5.095573000	12.499482000
6	9.101508000	6.188505000	11.476820000
6	10.627965000	6.268409000	11.399841000
1	10.922873000	6.996164000	10.630090000
1	11.044951000	5.281696000	11.154175000
1	11.034523000	6.595539000	12.366775000
6	8.483652000	5.808300000	10.119442000
1	7.389277000	5.789303000	10.213956000
1	8.837482000	4.818314000	9.796777000
1	8.764478000	6.559452000	9.366738000
6	7.176937000	4.911106000	14.449640000
6	6.350608000	5.756535000	15.242551000
6	5.712932000	8.143506000	15.360964000
6	5.940076000	9.428404000	14.519767000
6	4.592906000	9.808077000	13.879860000
1	4.721064000	10.715729000	13.272219000
1	3.835391000	9.991472000	14.655584000
1	4.255705000	8.989081000	13.230148000
6	6.467099000	10.544679000	15.425458000
1	7.456590000	10.271536000	15.817257000
1	5.780034000	10.699304000	16.269042000

1	6.561994000	11.476853000	14.849961000
6	5.723968000	5.251273000	16.389722000
1	5.102146000	5.915613000	16.984480000
6	5.917450000	3.909590000	16.737549000
1	5.424610000	3.513019000	17.627752000
6	6.729001000	3.079145000	15.958620000
1	6.870369000	2.033450000	16.239964000
6	7.366063000	3.571148000	14.813787000
8	5.692283000	7.118628000	11.804848000
6	5.550954000	8.017342000	10.748248000
1	4.689075000	7.725906000	10.111112000
1	5.375171000	9.057449000	11.097673000
1	6.452240000	8.053325000	10.098994000

References

(1) Sur, S. K. Measurement of magnetic susceptibility and magnetic moment of paramagnetic molecules in solution by high-field Fourier transform NMR spectroscopy. *J. Magn. Reson.* **1989**, *82*, 169-173.

(2) Bain, G. A.; Berry, J. F. Diamagnetic corrections and Pascal's constants. *J. Chem. Educ.* **2008**, *85*, 532-536.

(3) Sheldrick, G. M. A short history of SHELX. *Acta Crystallogr., Sect. A Found. Crystallogr.* **2008**, *A64*, 112-122.

(4) Macrae, C. F.; Bruno, I. J.; Chisholm, J. A.; Edgington, P. R.; McCabe, P.; Pidcock, E.; Rodriguez-Monge, L.; Taylor, R.; van de Streek, J.; Wood, P. A. Mercury CSD 2.0 - new features for the visualization and investigation of crystal structures. *J. Appl. Crystallogr.* **2008**, *41*, 466-470.

(5) Gupta, M.; Kumar, Y.; Tayal, A.; Pandey, N.; Caliebe, W.; Stahn, J. X-ray absorption spectroscopy study of cobalt mononitride thin films. *SN Appl. Sci.* **2020**, *2*, 41.

(6) Ravel, B.; Newville, M. ATHENA, ARTEMIS, HEPHAESTUS: data analysis for x-ray absorption spectroscopy using IFEFFIT. *J. Synchrotron Radiat.* **2005**, *12*, 537-541.

(7) Rehr, J. J.; Albers, R. C. Theoretical approaches to x-ray absorption fine structure. *Rev. Mod. Phys.* **2000**, *72*, 621-654.

(8) Koningsberger, D. C.; Prins, R.; Editors. *X-ray Absorption: Principles, Applications, Techniques of EXAFS SEXAFS, and XANES*; 1988.

(9) Neese, F. The ORCA program system. *Wiley Interdiscip. Rev. Comput. Mol. Sci.* **2012**, *2*, 73-78.

(10) Becke, A. D. Density-functional exchange-energy approximation with correct asymptotic behavior. *Phys. Rev. A Gen. Phys.* **1988**, *38*, 3098-3100.

(11) Perdew. Density-functional approximation for the correlation energy of the inhomogeneous electron gas. *Phys Rev B Condens Matter* **1986**, *33*, 8822-8824.

(12) Weigend, F.; Ahlrichs, R. Balanced basis sets of split valence, triple zeta valence and quadruple zeta valence quality for H to Rn: Design and assessment of accuracy. *Phys. Chem. Chem. Phys.* **2005**, *7*, 3297-3305.
(13) Grimme, S.; Antony, J.; Ehrlich, S.; Krieg, H. A consistent and accurate ab initio parametrization of density functional dispersion correction (DFT-D) for the 94 elements H-Pu. *J. Chem. Phys.* **2010**, *132*, 154104/154101-154104/154119.
(14) Grimme, S.; Ehrlich, S.; Goerigk, L. Effect of the damping function in dispersion corrected density functional theory. *J. Comput. Chem.* **2011**, *32*, 1456-1465.
(15) Barone, V.; Cossi, M. Quantum Calculation of Molecular Energies and Energy Gradients in Solution by a Conductor Solvent Model. *J. Phys. Chem. A* **1998**, *102*, 1995-2001.
(16) Kossmann, S.; Neese, F. Efficient Structure Optimization with Second-Order Many-Body Perturbation Theory: The RIJCOSX-MP2 Method. *J. Chem. Theory Comput.* **2010**, *6*, 2325-2338.
(17) DeBeer George, S.; Petrenko, T.; Neese, F. Prediction of Iron K-Edge Absorption Spectra Using Time-Dependent Density Functional Theory. *J. Phys. Chem. A* **2008**, *112*, 12936-12943.
(18) Gotico, P.; Moonshiram, D.; Liu, C.; Zhang, X.; Guillot, R.; Quaranta, A.; Halime, Z.; Leibl, W.; Aukauloo, A. Spectroscopic characterisation of a bio-inspired Ni-based proton reduction catalyst bearing a pentadentate N2S3 ligand with improved photocatalytic activity. *Chem. - Eur. J.* **2020**, *26*, 2859-2868.
(19) Roemelt, M.; Beckwith, M. A.; Duboc, C.; Collomb, M.-N.; Neese, F.; DeBeer, S. Manganese K-Edge X-Ray Absorption Spectroscopy as a Probe of the Metal-Ligand Interactions in Coordination Compounds. *Inorg. Chem.* **2012**, *51*, 680-687.
(20) Iglesias, S.; Gamonal, A.; Abudulimu, A.; Picon, A.; Carrasco, E.; Ecija, D.; Liu, C.; Luer, L.; Zhang, X.; Costa, J. S.; Moonshiram, D. Tracking the Light-Induced Excited-State Dynamics and Structural Configurations of an Extraordinarily Long-Lived Metastable State at Room Temperature. *Chem. - Eur. J.* **2020**, *26*, 10801-10810.
(21) Moonshiram, D.; Garrido-Barros, P.; Gimbert-Surinach, C.; Picon, A.; Liu, C.; Zhang, X.; Karnahl, M.; Llobet, A. Elucidating the Nature of the Excited State of a Heteroleptic Copper Photosensitizer by using Time-Resolved X-ray Absorption Spectroscopy. *Chem. - Eur. J.* **2018**, *24*, 6464-6472.
(22) Rentschler, M.; Iglesias, S.; Schmid, M.-A.; Liu, C.; Tschierlei, S.; Frey, W.; Zhang, X.; Karnahl, M.; Moonshiram, D. The Coordination Behaviour of CuI Photosensitizers Bearing Multidentate Ligands Investigated by X-ray Absorption Spectroscopy. *Chem. - Eur. J.* **2020**, *26*, 9527-9536.
(23) Moonshiram, D.; Guda, A.; Kohler, L.; Picon, A.; Guda, S.; Lehmann, C. S.; Zhang, X.; Southworth, S. H.; Mulfort, K. L. Mechanistic Evaluation of a Nickel Proton Reduction Catalyst Using Time-Resolved X-ray Absorption Spectroscopy. *J. Phys. Chem. C* **2016**, *120*, 20049-20057.
(24) Moonshiram, D.; Gimbert-Surinach, C.; Guda, A.; Picon, A.; Lehmann, C. S.; Zhang, X.; Doumy, G.; March, A. M.; Benet-Buchholz, J.; Soldatov, A.; Llobet, A.; Southworth, S. H. Tracking the Structural and Electronic Configurations of a Cobalt Proton Reduction Catalyst in Water. *J. Am. Chem. Soc.* **2016**, *138*, 10586-10596.
(25) Wojdyr, M. Fityk: a general-purpose peak fitting program. *J. Appl. Crystallogr.* **2010**, *43*, 1126-1128.
(26) Pattanayak, S.; Jasniewski, A. J.; Rana, A.; Draksharapu, A.; Singh, K. K.; Weitz, A.; Hendrich, M.; Que, L.; Dey, A.; Sen Gupta, S. Spectroscopic and Reactivity Comparisons of a Pair of bTAML Complexes with FeV=O and FeIV=O Units. *Inorg. Chem.* **2017**, *56*, 6352-6361.

(27) Griffith, J. S.; Orgel, L. E. Ligand-field theory. *Quarterly Reviews, Chemical Society* **1957**, *11*, 381-393.
(28) Hansch, C.; Leo, A.; Taft, R. W. A survey of Hammett substituent constants and resonance and field parameters. *Chem. Rev.* **1991**, *91*, 165-195.
(29) Fukuzumi, S.; Shimoosako, K.; Suenobu, T.; Watanabe, Y. Mechanisms of Hydrogen-, Oxygen-, and Electron-Transfer Reactions of Cumylperoxyl Radical. *J. Am. Chem. Soc.* **2003**, *125*, 9074-9082.
